# Supplementary material for: The repressor Capicua is a barrier to lung tumor development driven by Kras/Trp53 mutations
Source: EMBO Mol Med. 2025 Nov 11;17(12):3377–406. doi: 10.1038/s44321-025-00326-z (PMC12686060; doi:10.1038/s44321-025-00326-z)
Supplement: Supplementary file 1 — Appendix [file 44321_2025_326_MOESM1_ESM.pdf]

APPENDIX

The repressor Capicua is a barrier to lung tumor development driven by *Kras/Trp53* mutations

Irene Ballesteros-González, Iván Hernández-Navas, Oksana Brehey, Carmen G. Lechuga, Marina Salmón, Morena Scotece, Ricardo Velasco-Vicente, Alejandra A. Flores-Gómez, Antonio Cebriá, Lucía Simón-Carrasco, Gerardo Jiménez, Monica Musteanu, Carmen Guerra, Orlando Domínguez, Eduardo Caleiras, Carmen Blanco-Aparicio, Tirso Pons, Irene Ferrer, Luis Paz-Ares, Raul Torres-Ruiz, Sandra Rodríguez-Perales, Mariano Barbacid, and Matthias Drosten

Table of contents

Appendix Figure S1……………………………………………………………..………2

Appendix Figure S2……………………………………………………………..…….3

Appendix Figure S3…………………………………………………………….……..4

Appendix Figure S4……………………………………………………………...…….5

Appendix Figure S5……………………………………………………………...……6

Fig. S1

A

Sample ID	*KRAS*	*TP53*	*CIC*
TCGA-69-7980-01A	G12A	A161V	E236*
TCGA-55-8302-01A	G12C	E343*	A263T
TCGA-86-8056-01A	G12D	E171K	R1412L

B

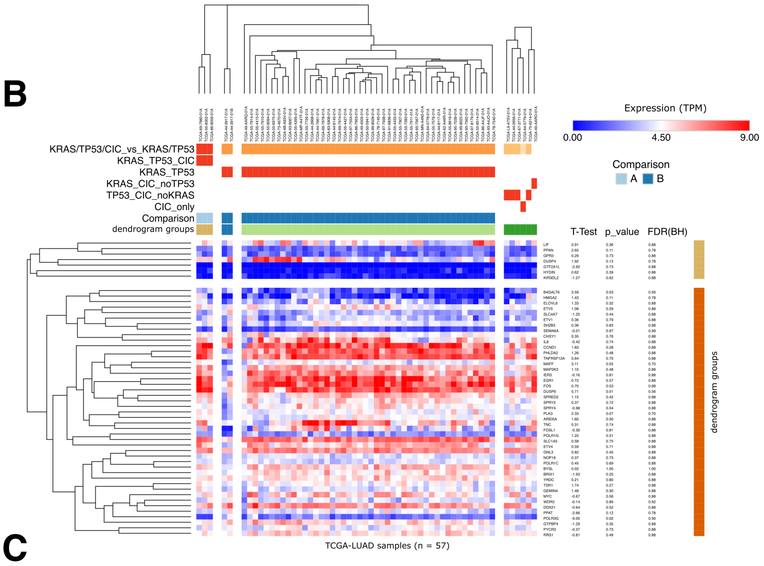

C

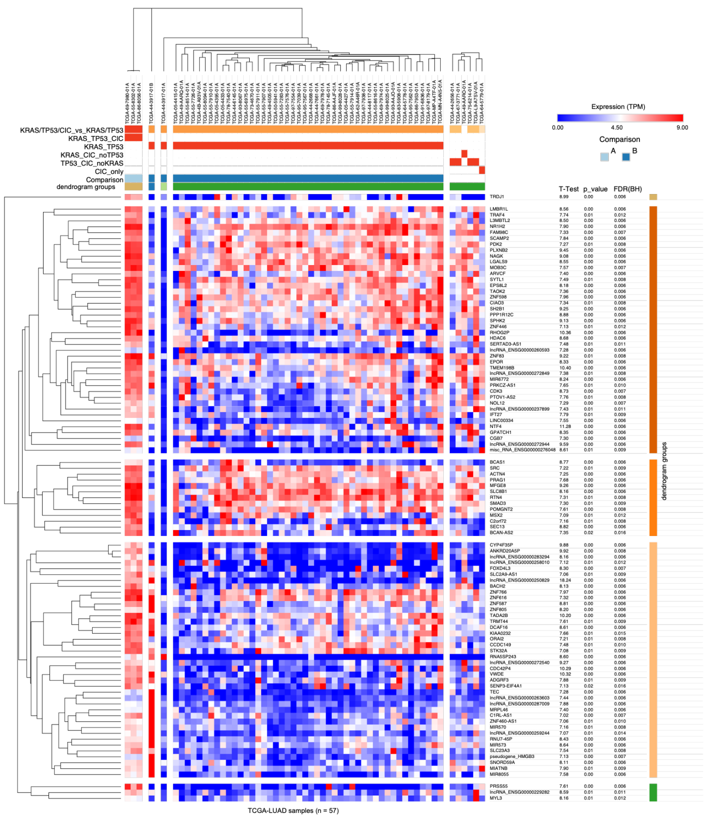

Appendix Figure S1: Expression profiling of *KRAS*/*TP53*/*CIC* mutant human LUAD. (A) Sample IDs and *KRAS*/*TP53*/*CIC* mutations in LUAD samples from the TCGA database. (B) Heatmap of expression levels of MAPK pathway-regulated genes in *KRAS*/*TP53* vs. *KRAS*/*TP53*/*CIC* mutant LUAD samples from the TCGA database. (C) Heatmap of expression levels of differentially expressed genes in *KRAS*/*TP53* vs. *KRAS*/*TP53*/*CIC* mutant LUAD samples from the TCGA database.

Fig. S2

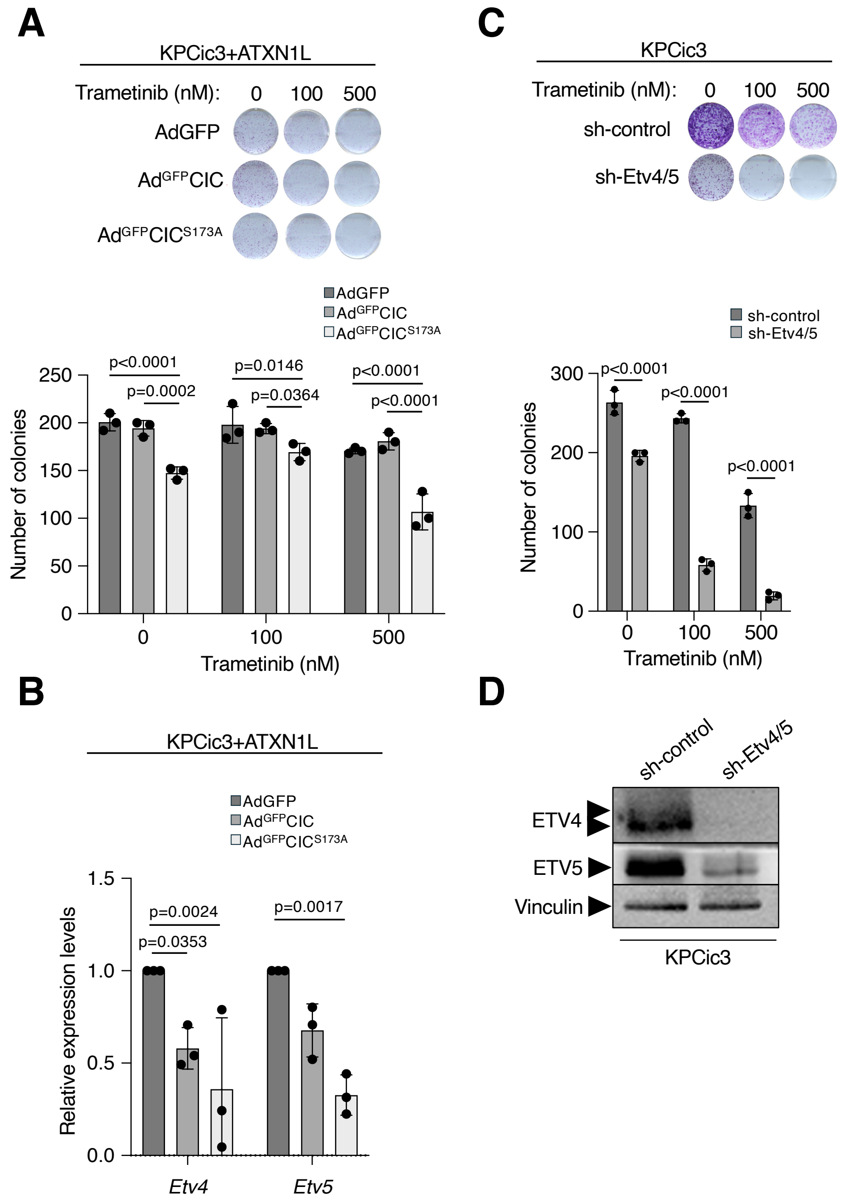

Appendix Figure S2: Validation of a third KPCic cell line (A) Representative images and quantification of colony formation assays in a third KPCic cell line stably expressing HA-ATXN1L after infection with Ad-GFP (dark gray bars), Ad-^{GFP}CIC (gray bars) or Ad-^{GFP}CIC^{S173A} (light gray bars). Cells were continuously treated with the indicated concentrations of trametinib. Results are shown as mean ± SD. Statistics, two-way ANOVA with Tukey's multiple comparison test. (B) qRT-PCR of *Etv4* and *Etv5* expression levels in a third KPCic cell line stably expressing HA-ATXN1L 72h after infection with Ad-GFP (dark gray bars), Ad-^{GFP}CIC (gray bars) or Ad-^{GFP}CIC^{S173A} (light gray bars). Results are shown as mean ± SD. Statistics, one-way ANOVA with Tukey's multiple comparison test. (C) Representative images and quantification of colony formation assays in a third KPCic cell line stably expressing a control shRNA (dark gray bars) or a combination of shRNAs targeting *Etv4* and *Etv5* (light gray bars). Cells were continuously treated with the indicated concentrations of trametinib. Results are shown as mean ± SD. Statistics, two-way ANOVA with Sidak's multiple comparison test. (D) Western blot analysis of ETV4 and ETV5 expression in a third KPCic cell line stably expressing a control shRNA or a combination of shRNAs targeting *Etv4* and *Etv5*. Vinculin expression served as a loading control.

Fig. S3

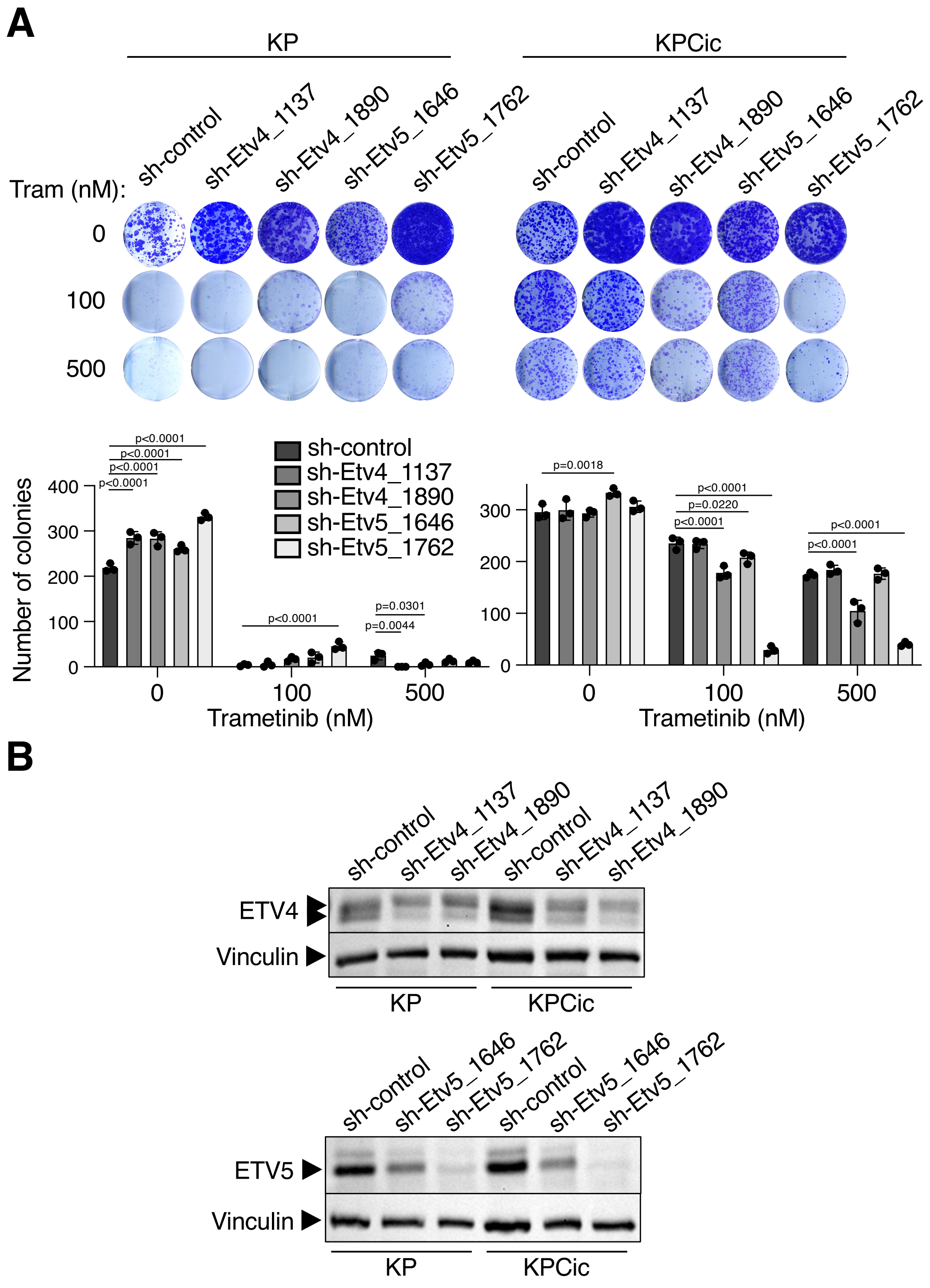

Appendix Figure S3: Silencing of *Etv4* or *Etv5* expression in KP and KPCic cells. (A) Representative images and quantification of colony formation assays in a KP and a KPCic cell line stably expressing a control shRNA, or two independent shRNAs targeting *Etv4* or *Etv5*. Cells were continuously treated with the indicated concentrations of trametinib. Results are shown as mean ± SD. Statistics, two-way ANOVA with Dunnett's multiple comparison test. (B) Western blot analysis of ETV4 and ETV5 expression in a KP and a KPCic cell line stably expressing a control shRNA, or two independent shRNAs targeting *Etv4* or *Etv5*. Vinculin expression served as a loading control.

Fig. S4

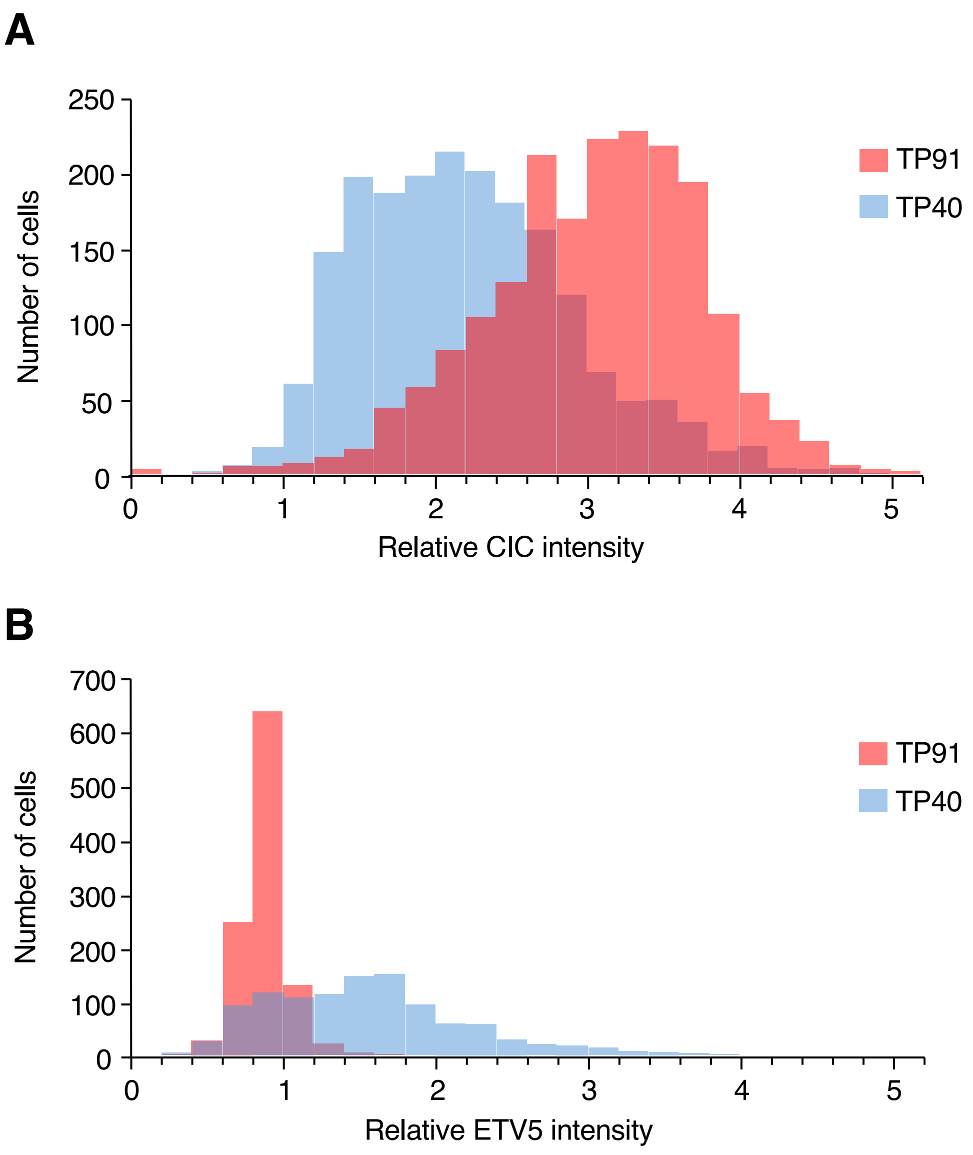

Appendix Figure S4: CIC and ETV5 expression in PDX tumors. (A) Relative intensities of CIC immunohistochemistry staining in nuclei from PDX tumors TP40 (blue) and TP91 (red). (B) Relative intensities of ETV5 immunohistochemistry staining in nuclei from PDX tumors TP40 (blue) and TP91 (red).

Fig. S5

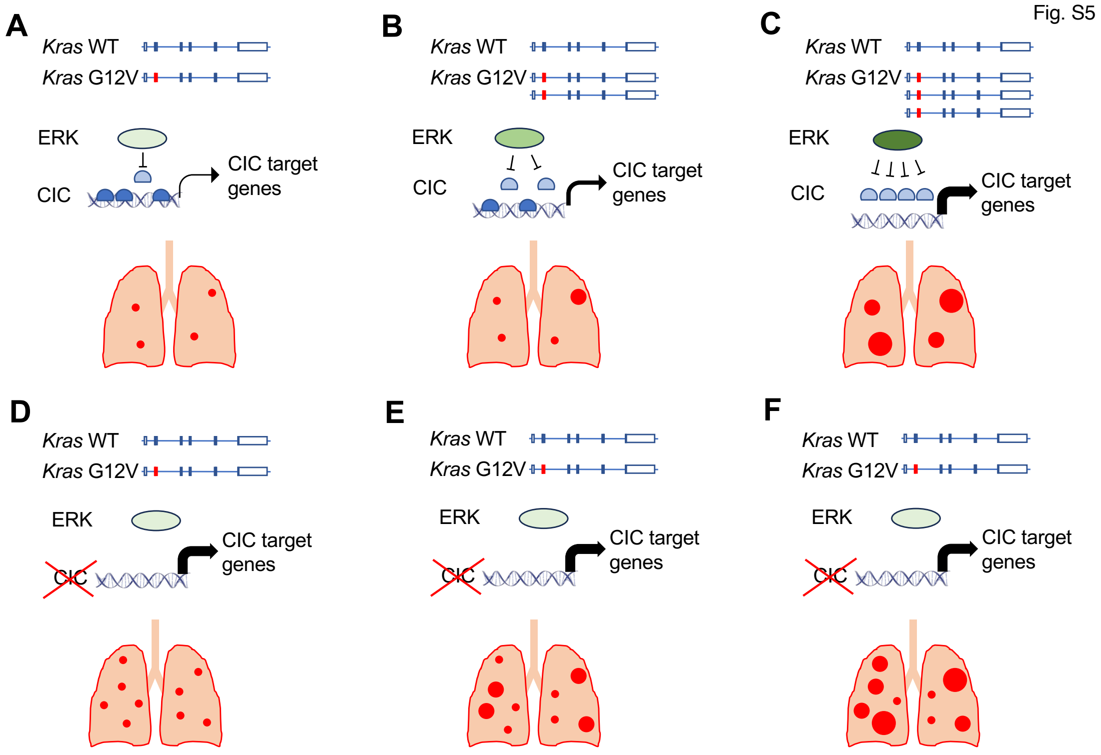

Appendix Figure S5: Model depicting the role of CIC in LUAD. (A) In CIC wild-type cells, a mutation in *Kras* has a minor effect on ERK activity and functional CIC inactivation by phosphorylation. (B) After some time, the tumor acquires additional copies of *Kras*, leading to enhanced ERK activity, functional CIC inactivation, and elevated expression of CIC targets. (C) Finally, after further gaining additional *Kras* copies, ERK activity becomes sufficient to completely inactivate CIC, allowing for tumor growth. (D-F) In the absence of CIC, tumor growth is efficient and does not require gaining additional *Kras* copies. Enhanced ERK activity is indicated as light to dark green. CIC bound to DNA and actively repressing its targets is shown in dark blue while inactive CIC is indicated in light blue. Coding and non-coding exons are represented by closed and open boxes, respectively. Exon 1 carrying the G12V mutation is highlighted in red.
