# Supplementary figures and images for: The repressor Capicua is a barrier to lung tumor development driven by Kras/Trp53 mutations
Source: EMBO Mol Med. 2025 Nov 11;17(12):3377–406. doi: 10.1038/s44321-025-00326-z (PMC12686060; doi:10.1038/s44321-025-00326-z)

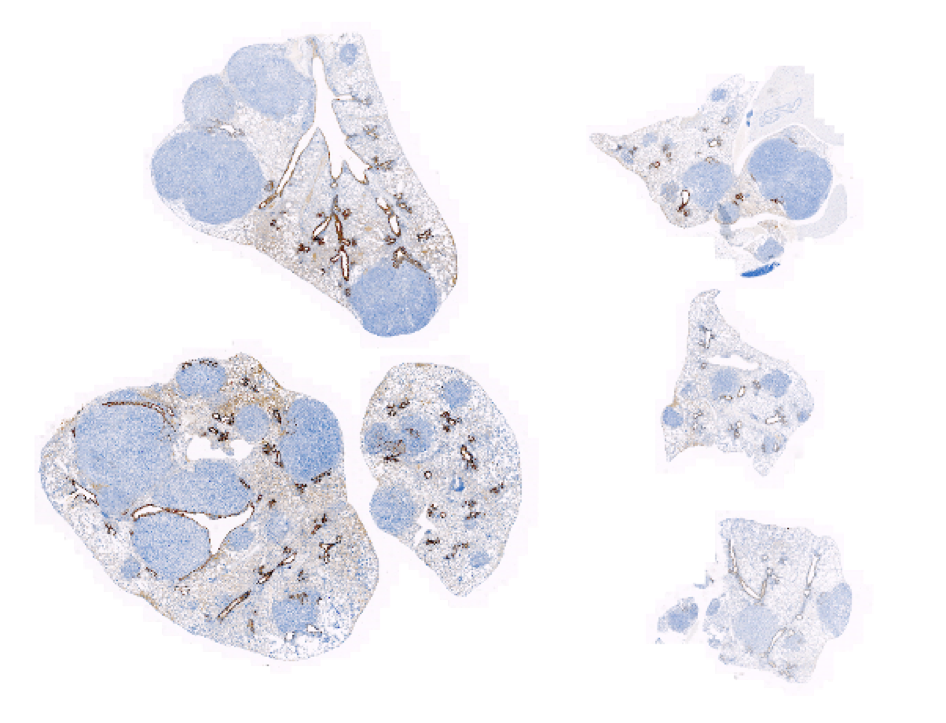

Supplement: Supplementary file 7 — Source data Fig. 1 [file 44321_2025_326_MOESM7_ESM.zip › Fig1/Fig1d/KPCic_CC10.tif]

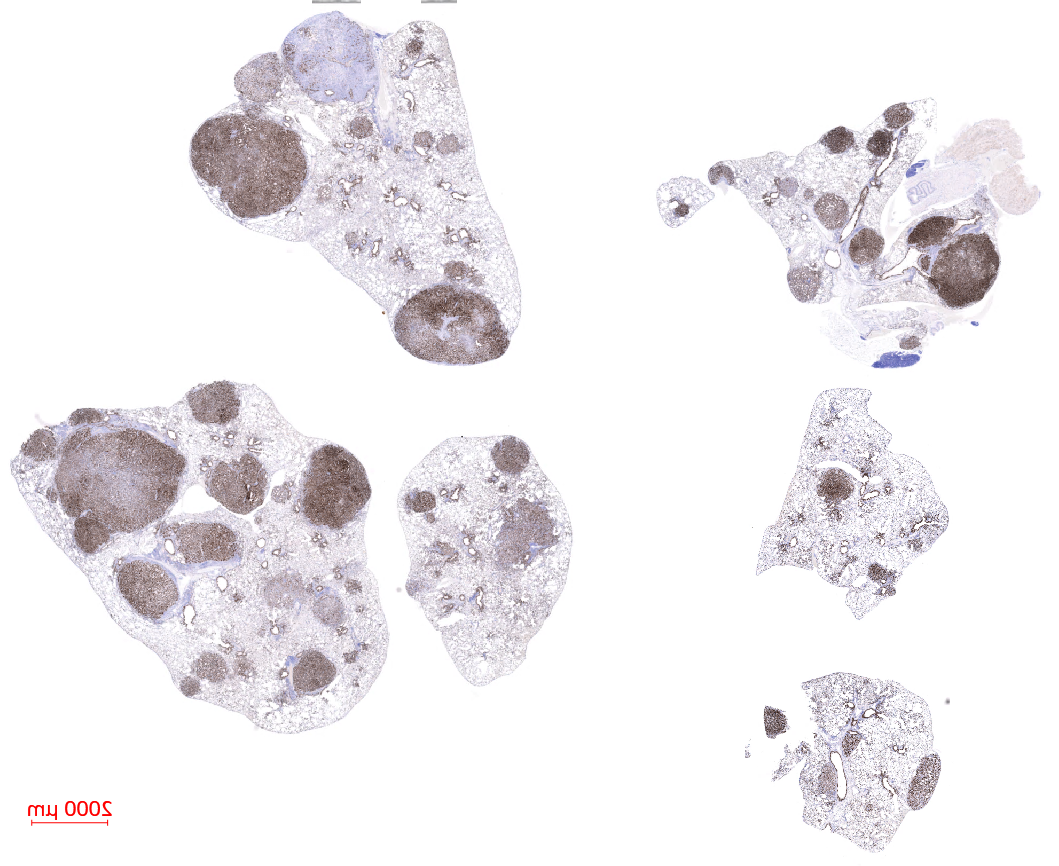

Supplement: Supplementary file 7 — Source data Fig. 1 [file 44321_2025_326_MOESM7_ESM.zip › Fig1/Fig1d/KPCic_TTF1.tiff]

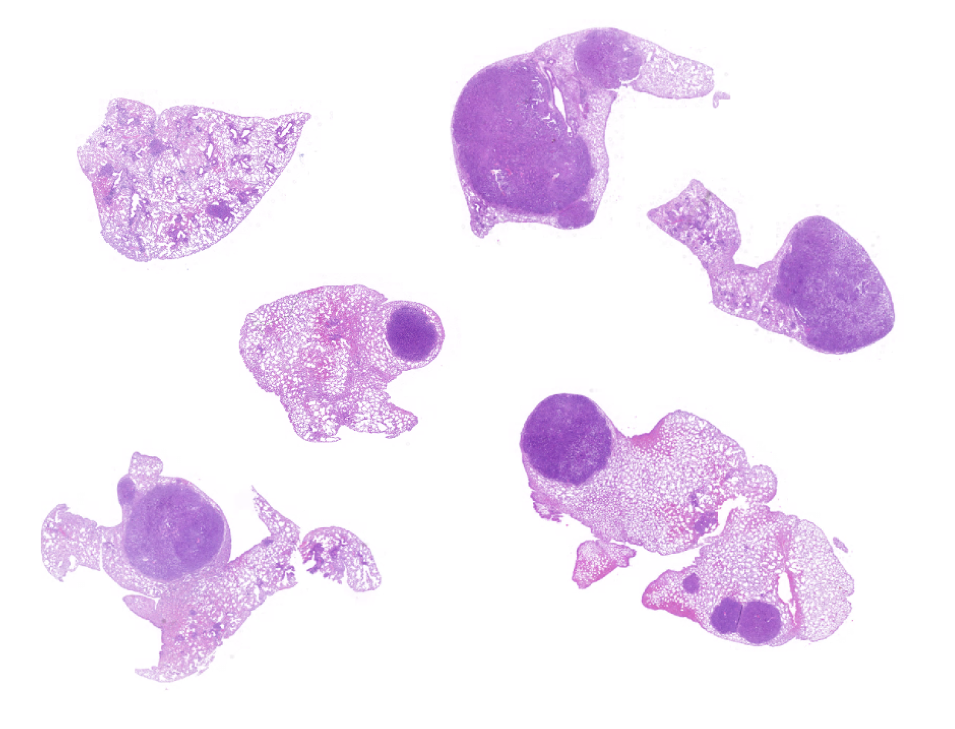

Supplement: Supplementary file 7 — Source data Fig. 1 [file 44321_2025_326_MOESM7_ESM.zip › Fig1/Fig1d/KP_HE.tif]

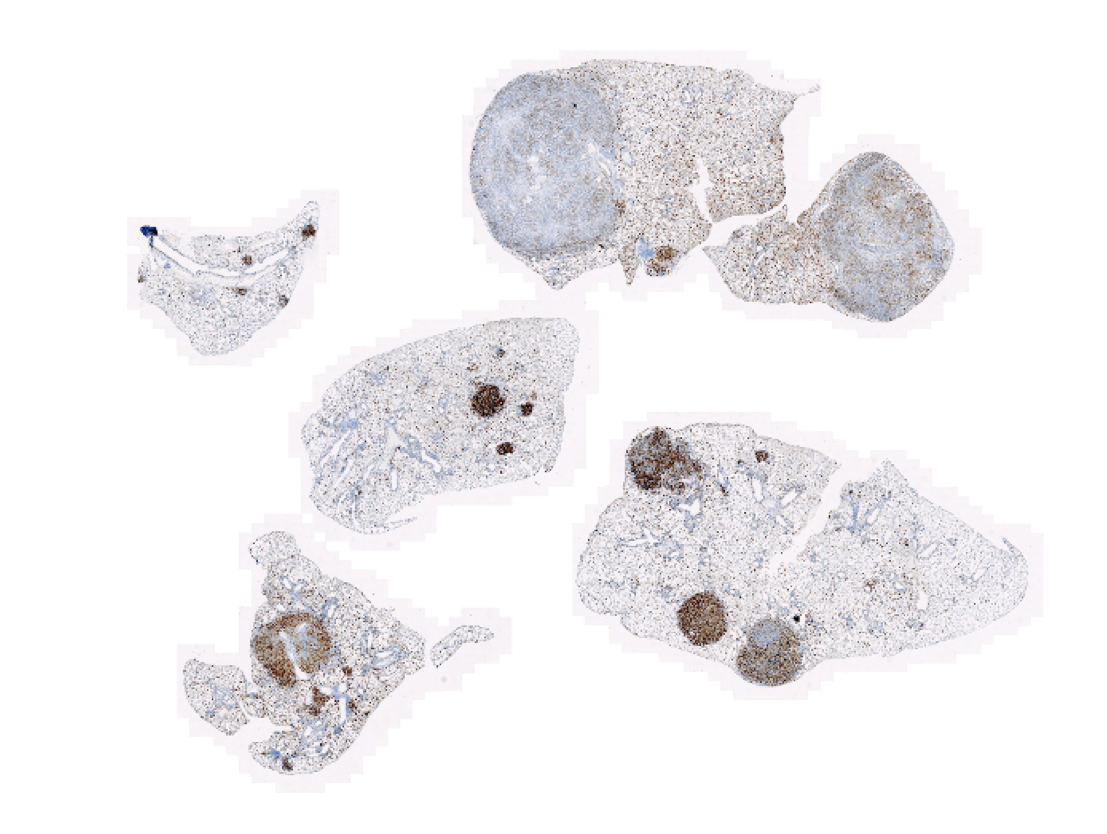

Supplement: Supplementary file 7 — Source data Fig. 1 [file 44321_2025_326_MOESM7_ESM.zip › Fig1/Fig1d/KP_SPC.tif]

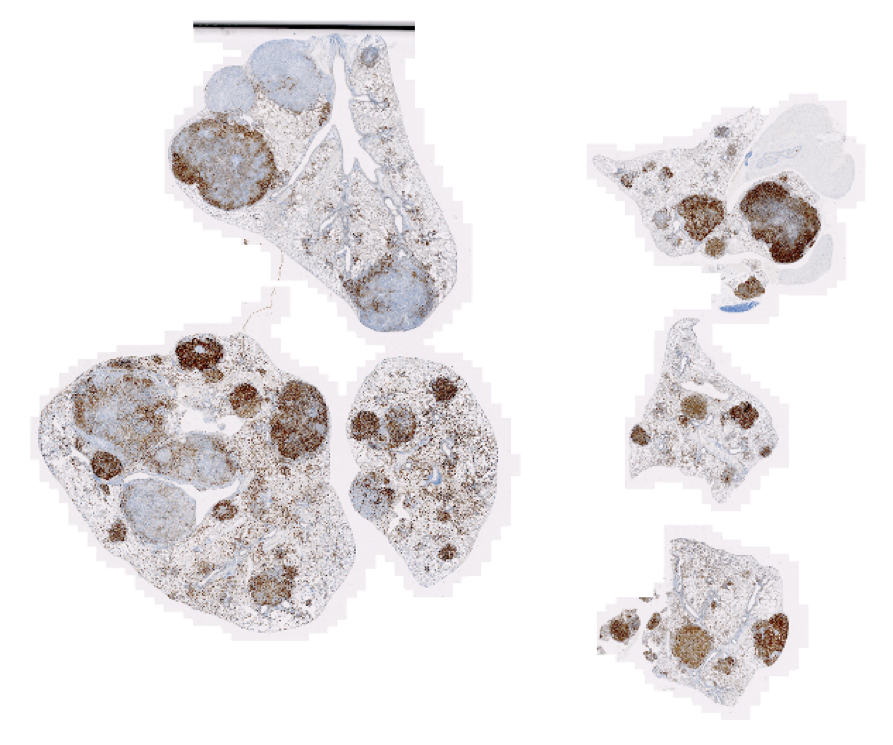

Supplement: Supplementary file 7 — Source data Fig. 1 [file 44321_2025_326_MOESM7_ESM.zip › Fig1/Fig1d/KPCic_SPC.tif]

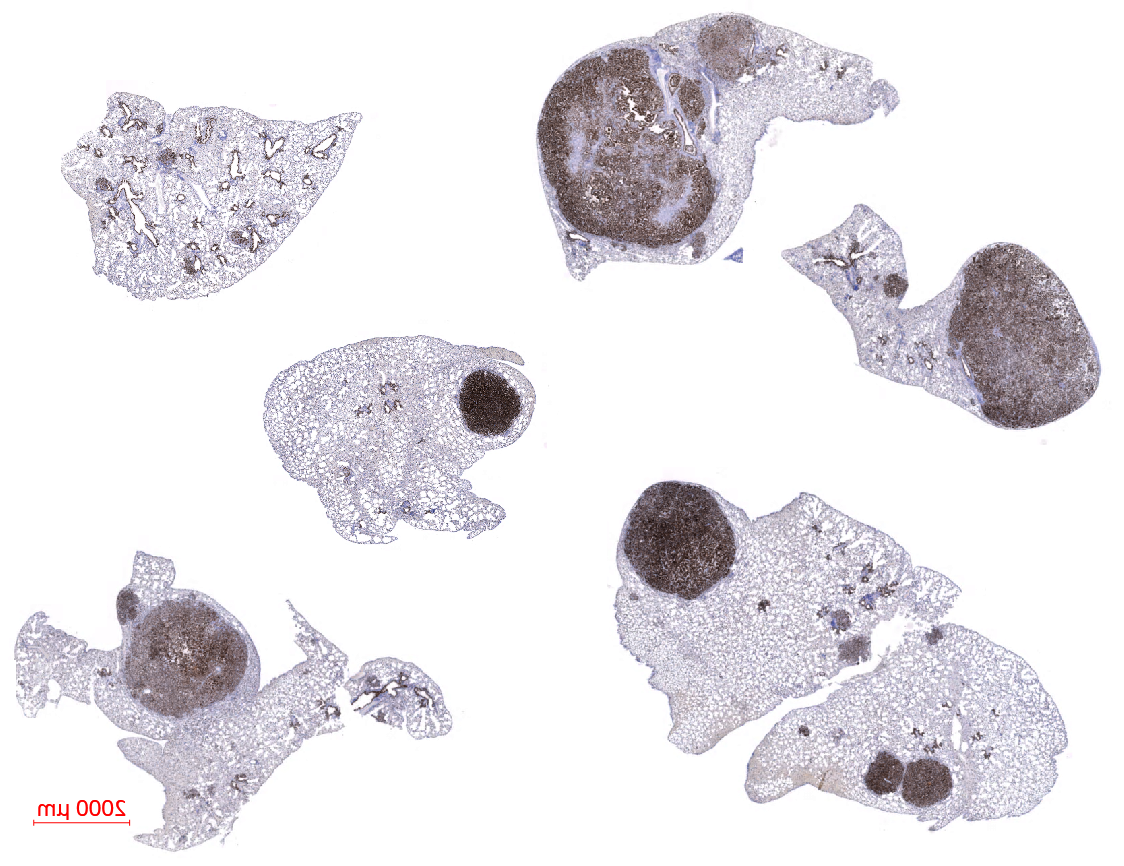

Supplement: Supplementary file 7 — Source data Fig. 1 [file 44321_2025_326_MOESM7_ESM.zip › Fig1/Fig1d/KP_TTF1.tiff]

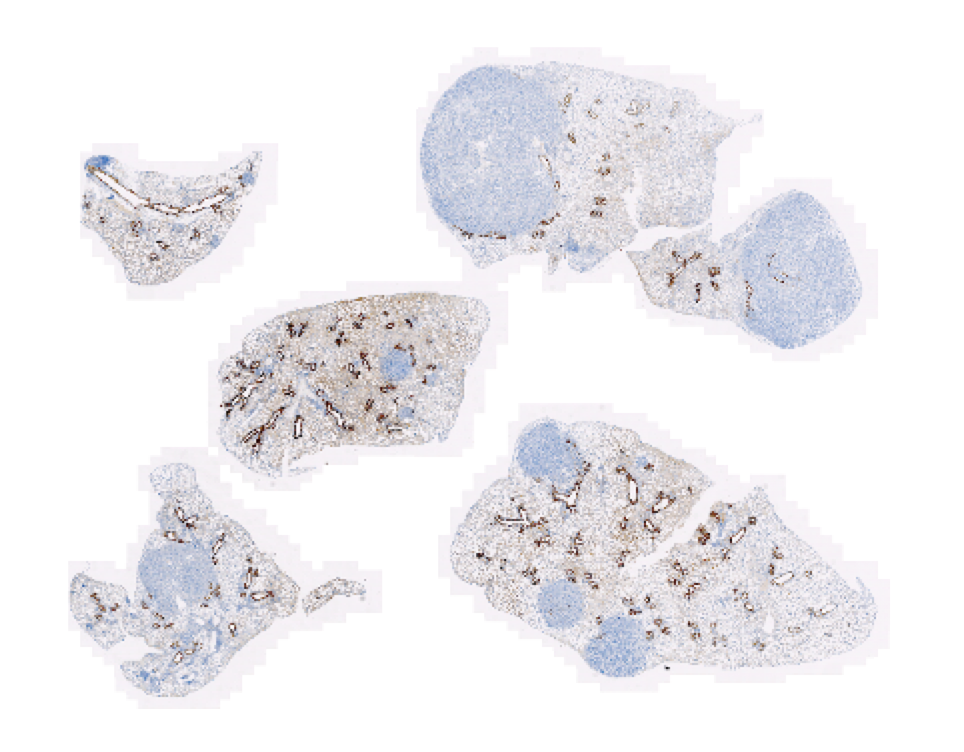

Supplement: Supplementary file 7 — Source data Fig. 1 [file 44321_2025_326_MOESM7_ESM.zip › Fig1/Fig1d/KP_CC10.tif]

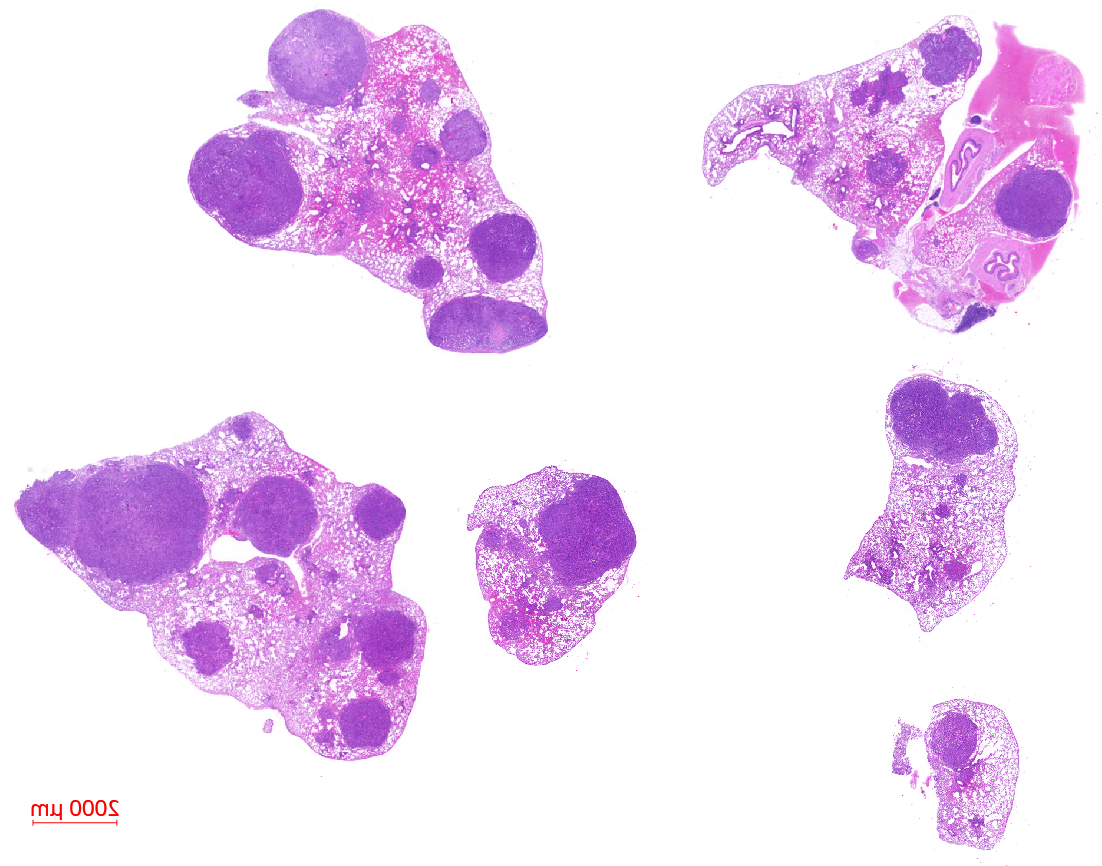

Supplement: Supplementary file 7 — Source data Fig. 1 [file 44321_2025_326_MOESM7_ESM.zip › Fig1/Fig1d/KPCic_HE.tiff]

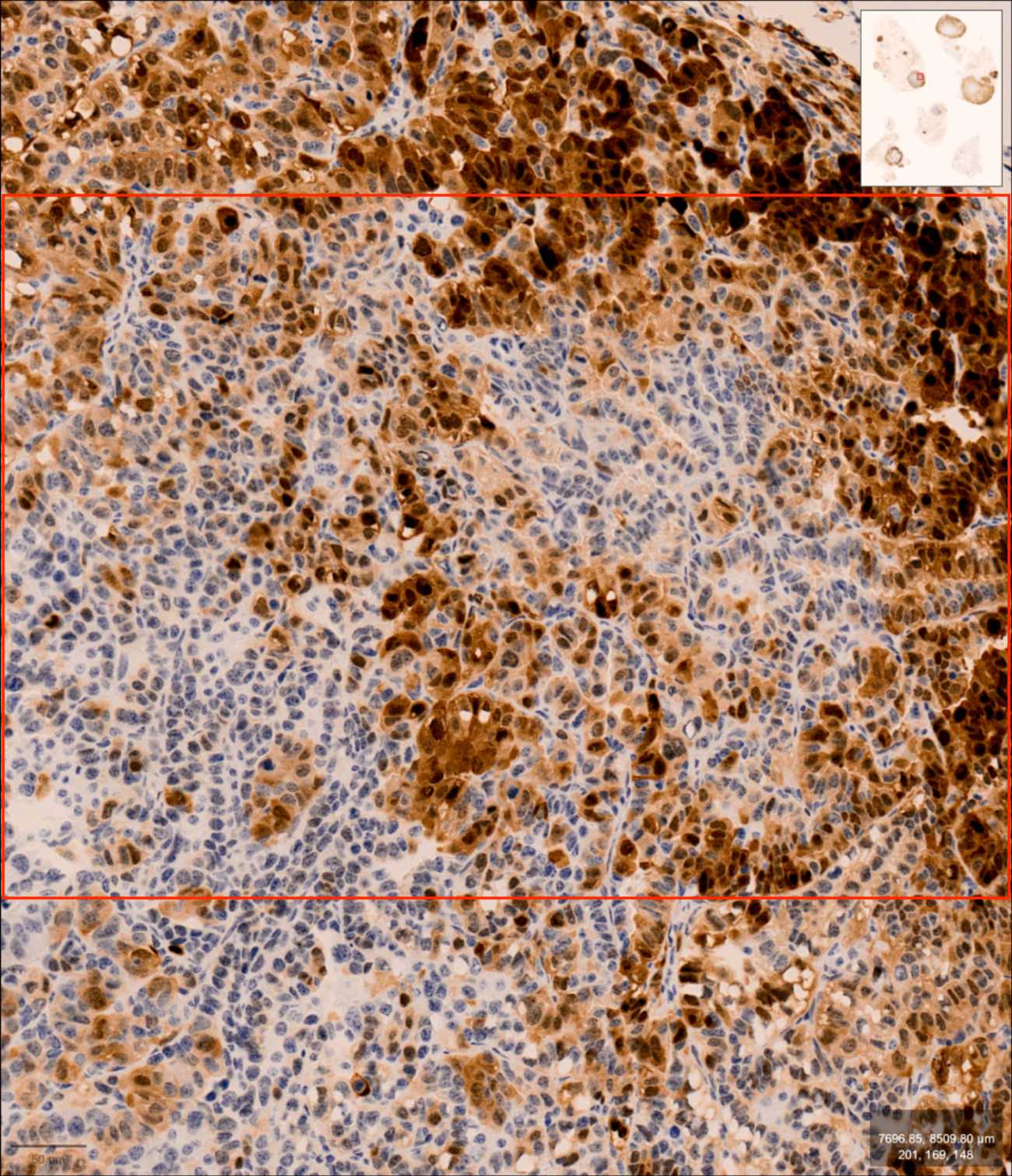

Supplement: Supplementary file 7 — Source data Fig. 1 [file 44321_2025_326_MOESM7_ESM.zip › Fig1/Fig1e/KP.tif]

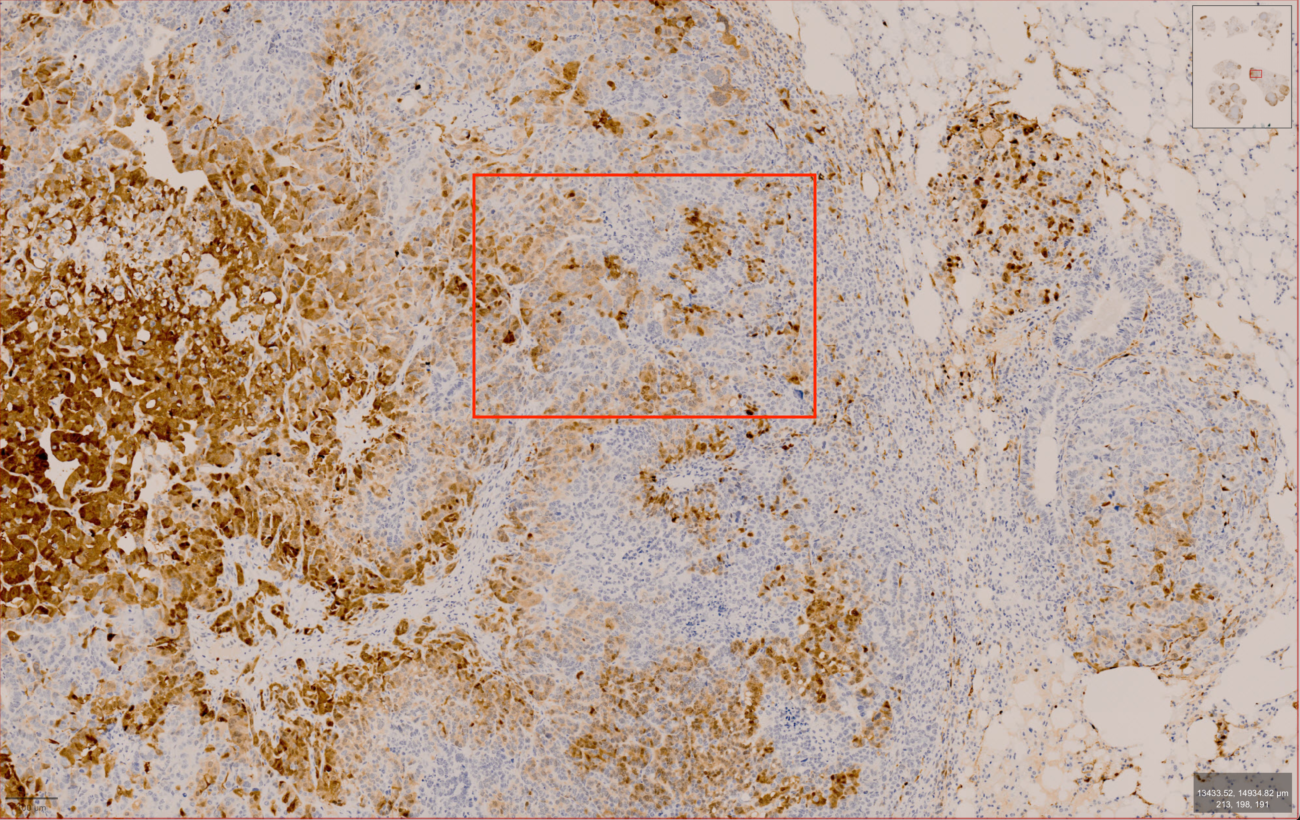

Supplement: Supplementary file 7 — Source data Fig. 1 [file 44321_2025_326_MOESM7_ESM.zip › Fig1/Fig1e/KPCic.tif]

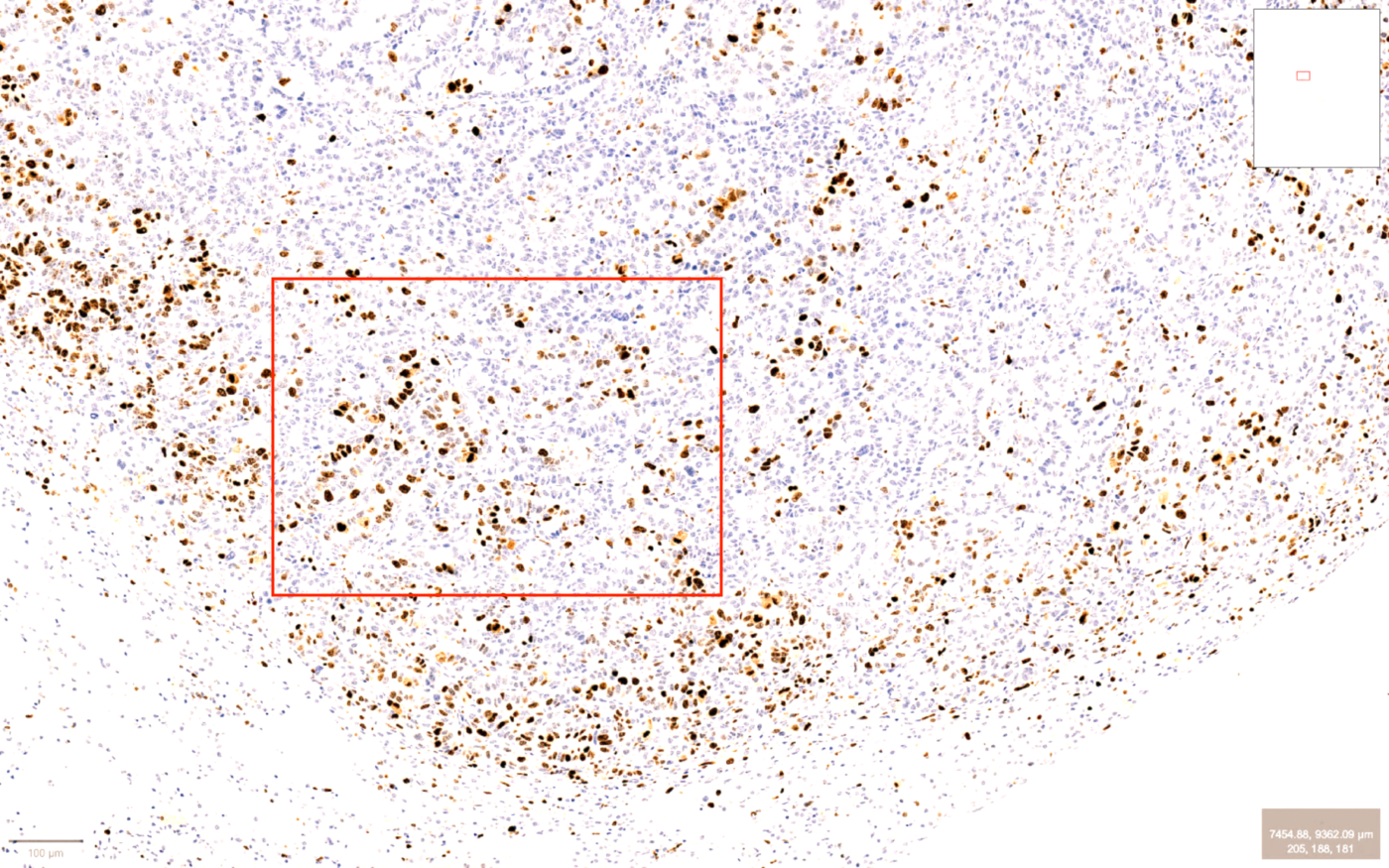

Supplement: Supplementary file 7 — Source data Fig. 1 [file 44321_2025_326_MOESM7_ESM.zip › Fig1/Fig1f/KP.tif]

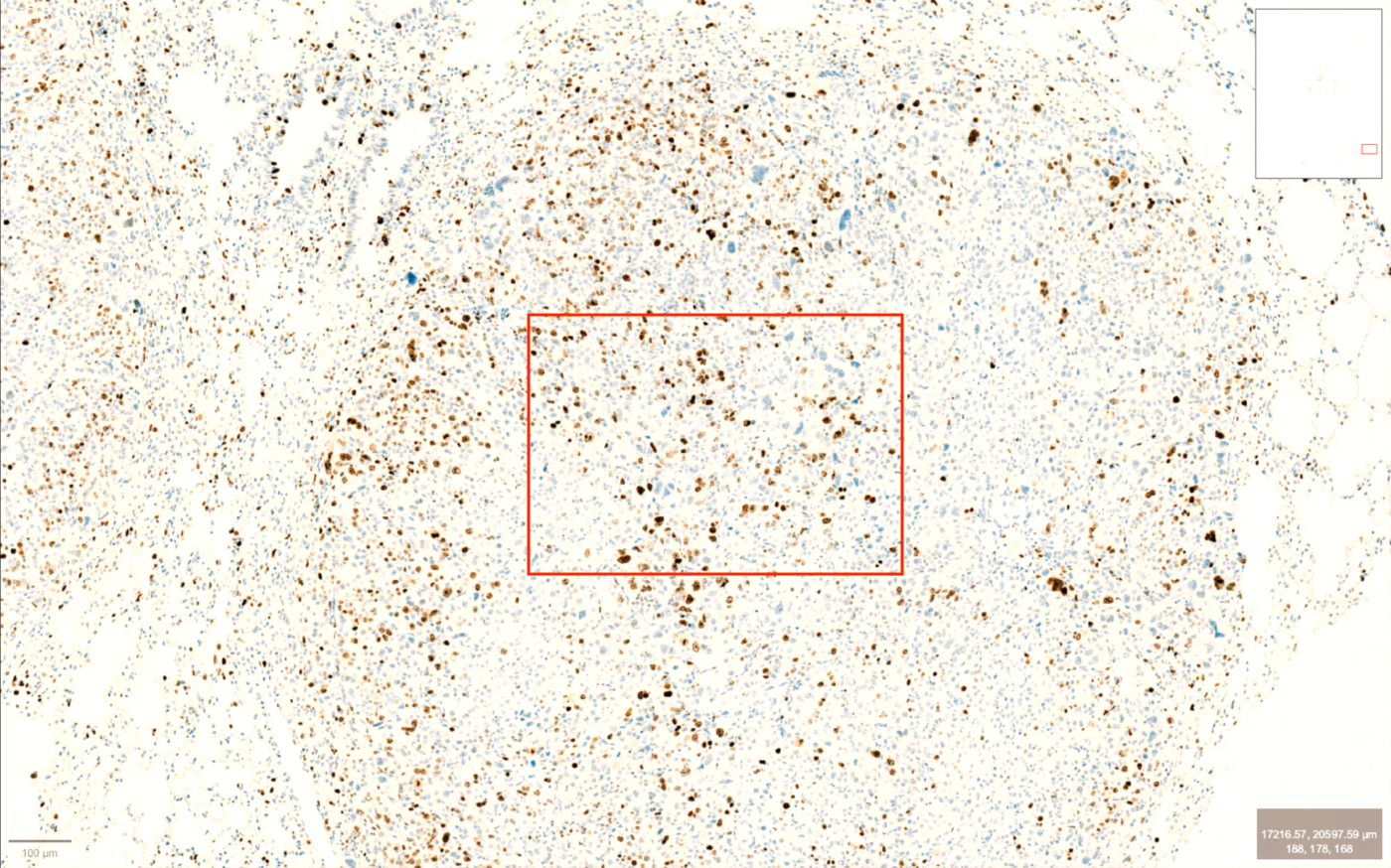

Supplement: Supplementary file 7 — Source data Fig. 1 [file 44321_2025_326_MOESM7_ESM.zip › Fig1/Fig1f/KPCic.tif]

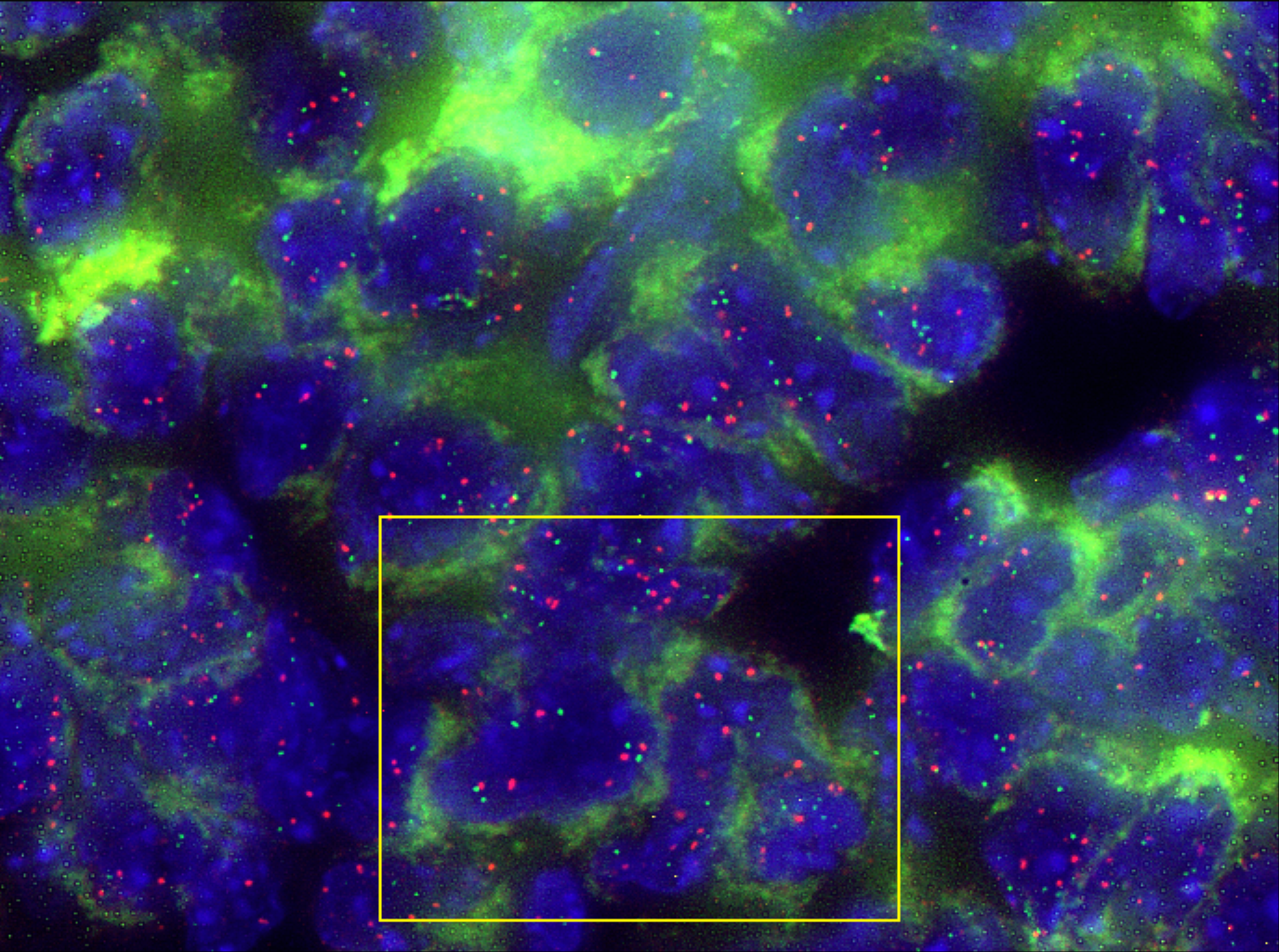

Supplement: Supplementary file 8 — Source data Fig. 2 [file 44321_2025_326_MOESM8_ESM.zip › Fig2/Fig2e/KP - FISH.tif]

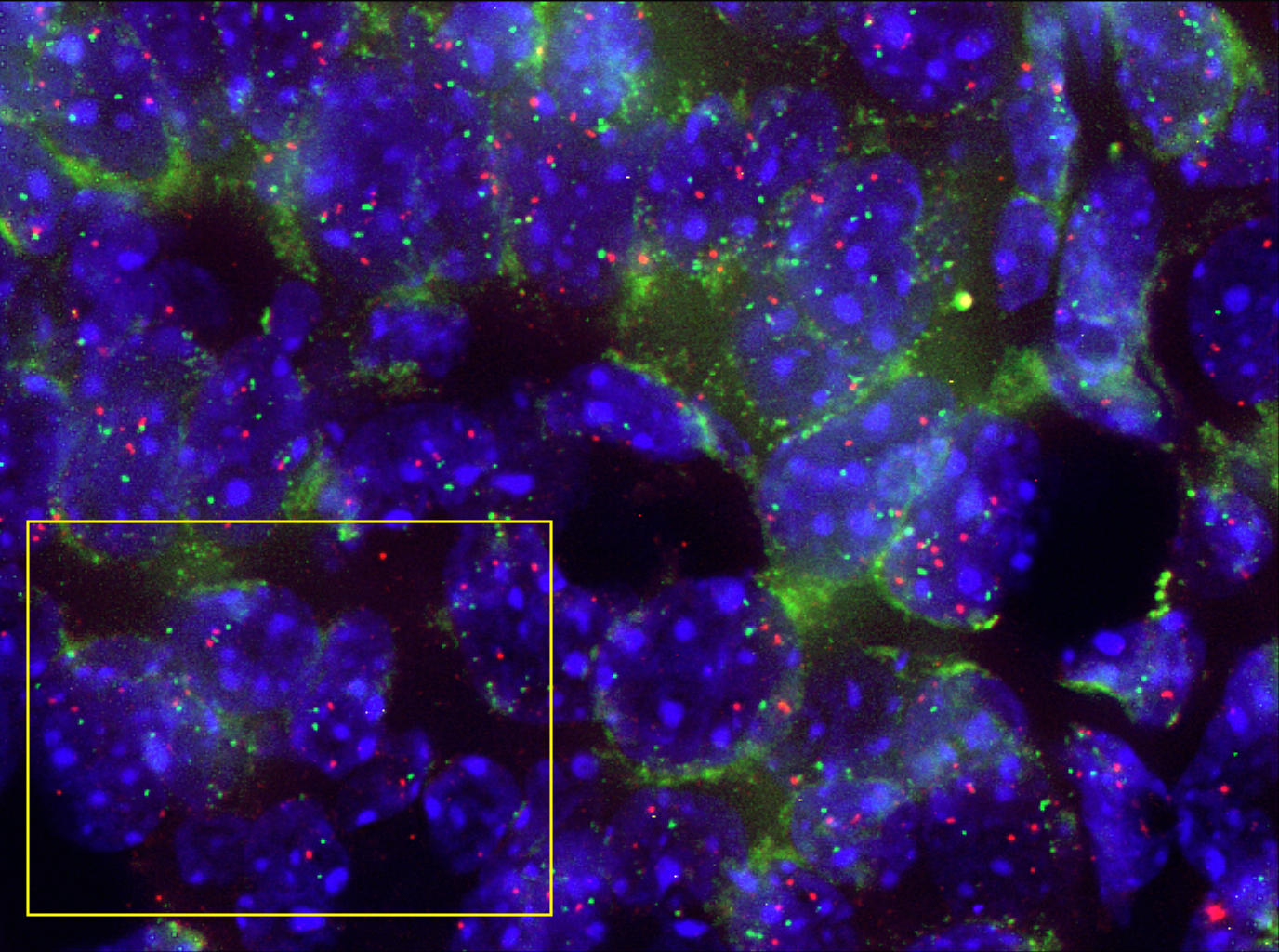

Supplement: Supplementary file 8 — Source data Fig. 2 [file 44321_2025_326_MOESM8_ESM.zip › Fig2/Fig2e/KPCic - FISH.tif]

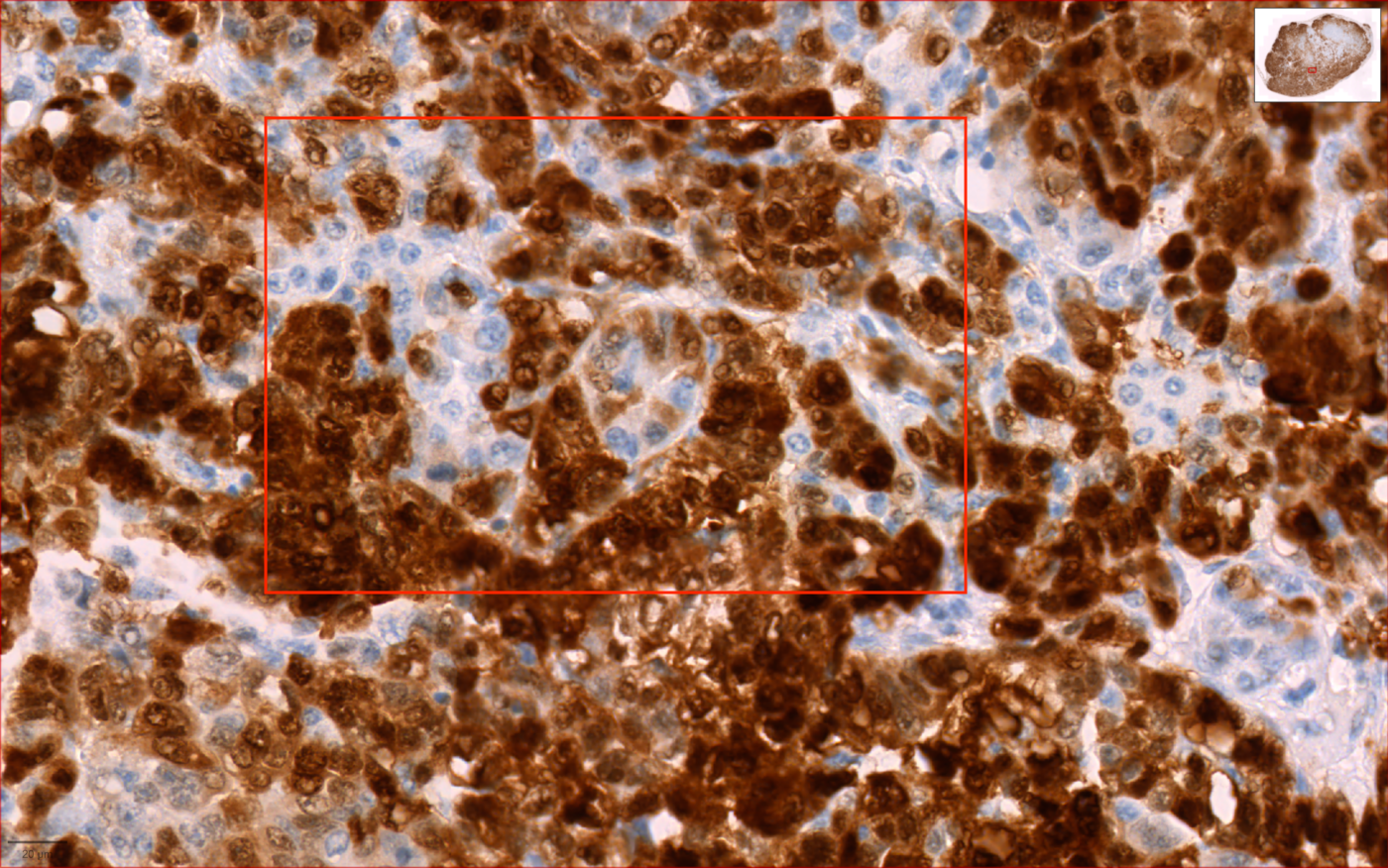

Supplement: Supplementary file 8 — Source data Fig. 2 [file 44321_2025_326_MOESM8_ESM.zip › Fig2/Fig2a/pERK - 7 months.tif]

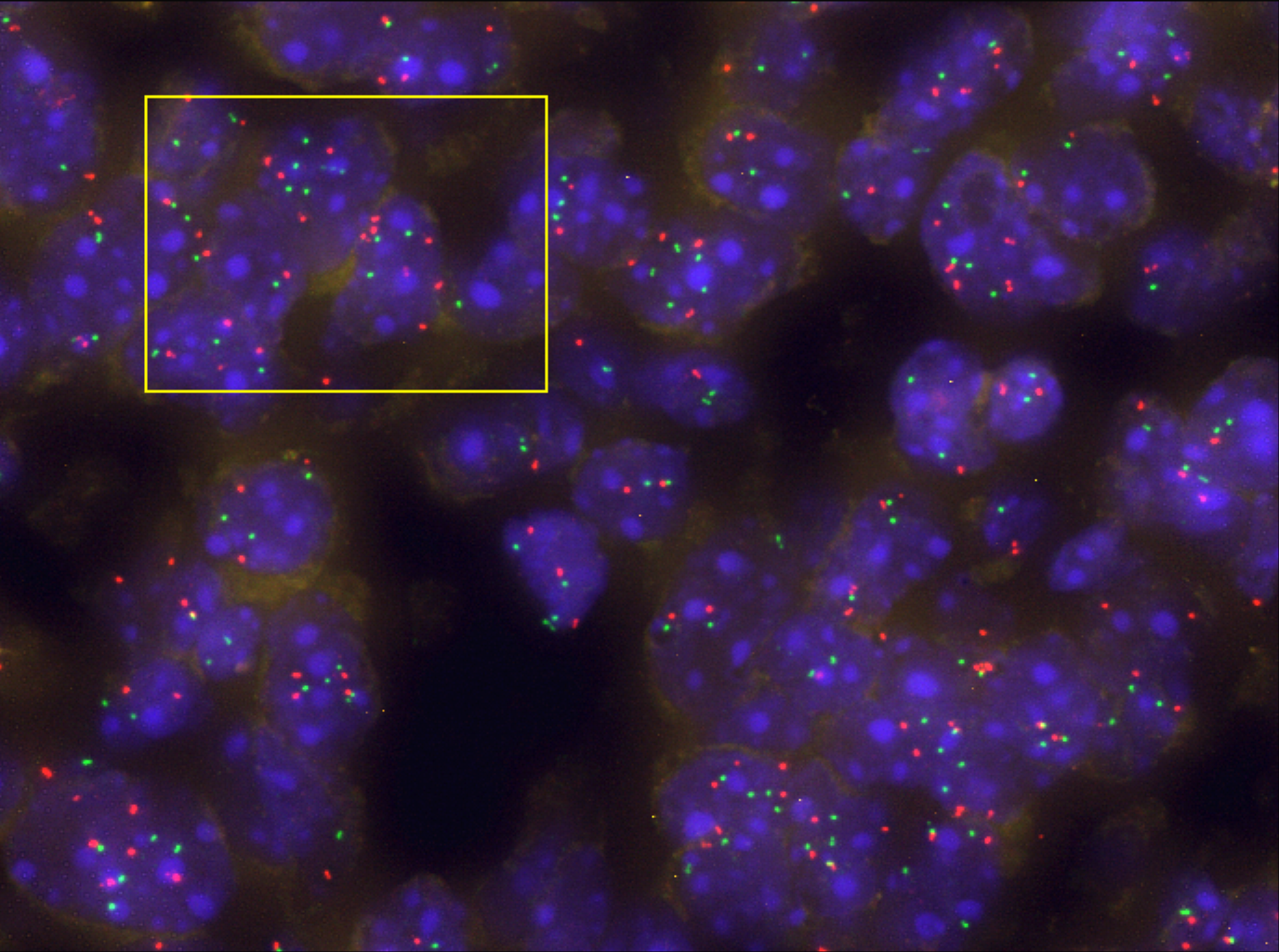

Supplement: Supplementary file 8 — Source data Fig. 2 [file 44321_2025_326_MOESM8_ESM.zip › Fig2/Fig2a/Kras FISH - 5 months.tif]

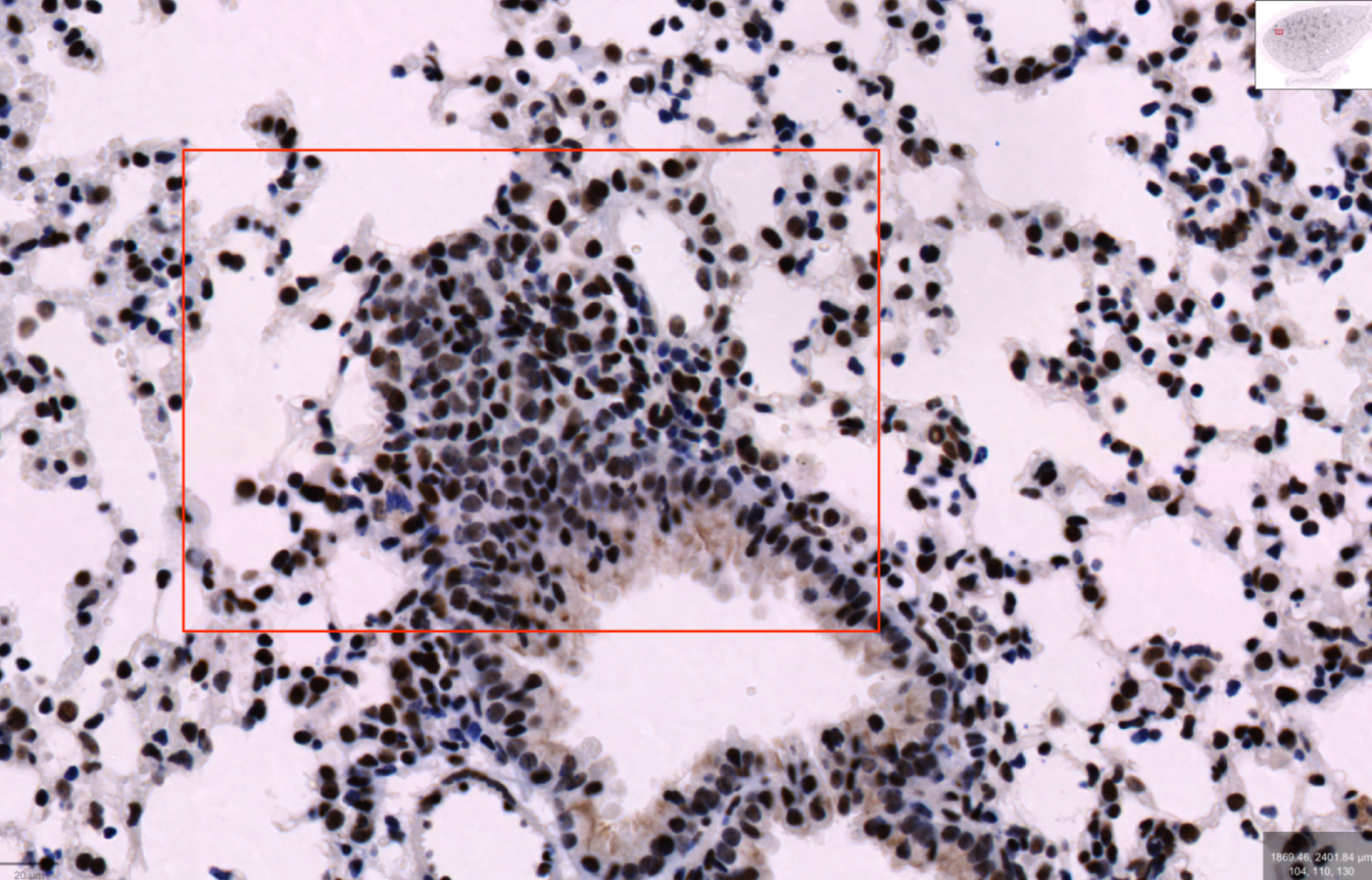

Supplement: Supplementary file 8 — Source data Fig. 2 [file 44321_2025_326_MOESM8_ESM.zip › Fig2/Fig2a/CIC - 1 month.tif]

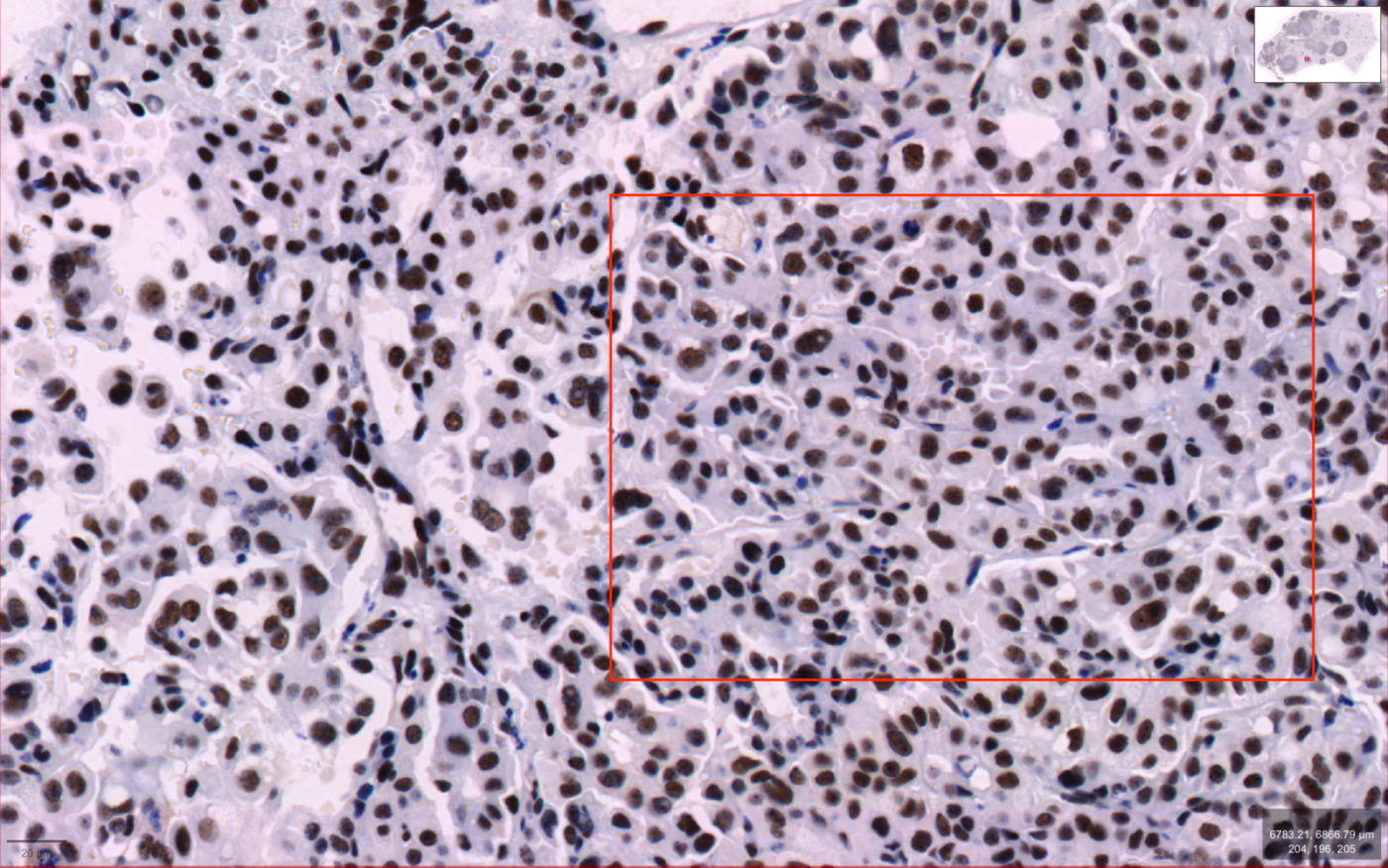

Supplement: Supplementary file 8 — Source data Fig. 2 [file 44321_2025_326_MOESM8_ESM.zip › Fig2/Fig2a/CIC - 3 months.tif]

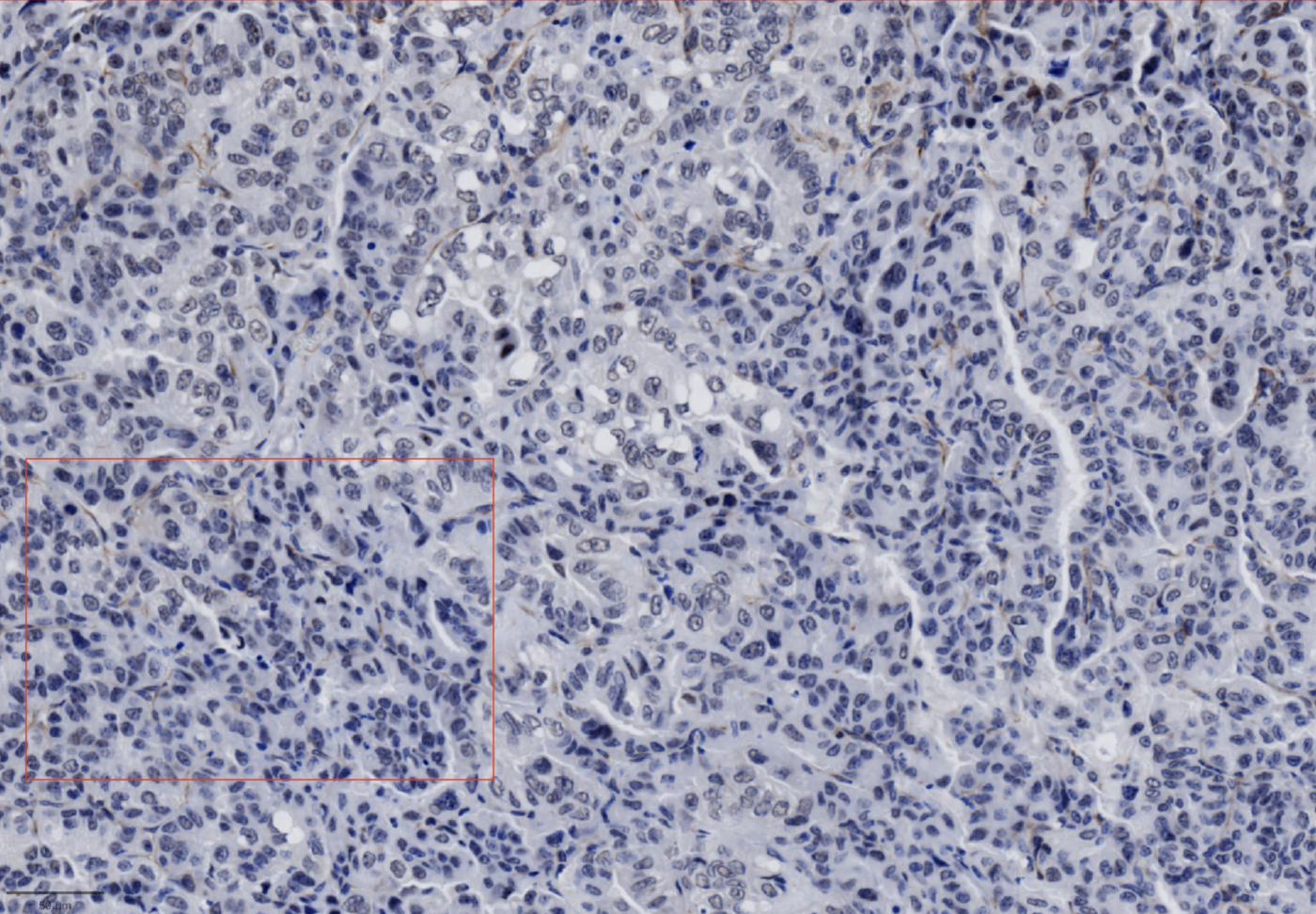

Supplement: Supplementary file 8 — Source data Fig. 2 [file 44321_2025_326_MOESM8_ESM.zip › Fig2/Fig2a/ETV5 - 3 months.tif]

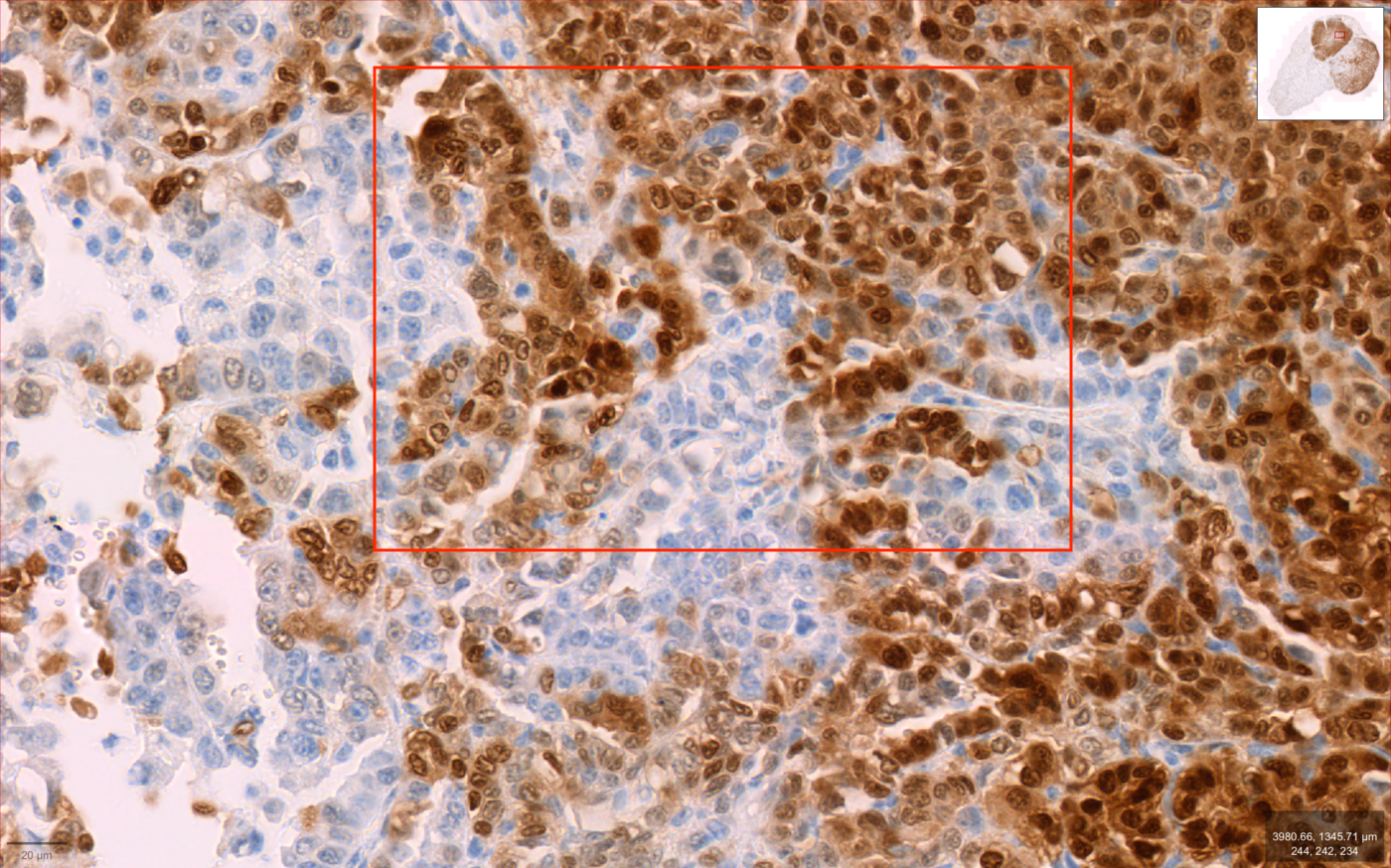

Supplement: Supplementary file 8 — Source data Fig. 2 [file 44321_2025_326_MOESM8_ESM.zip › Fig2/Fig2a/pERK - 5 months.tif]

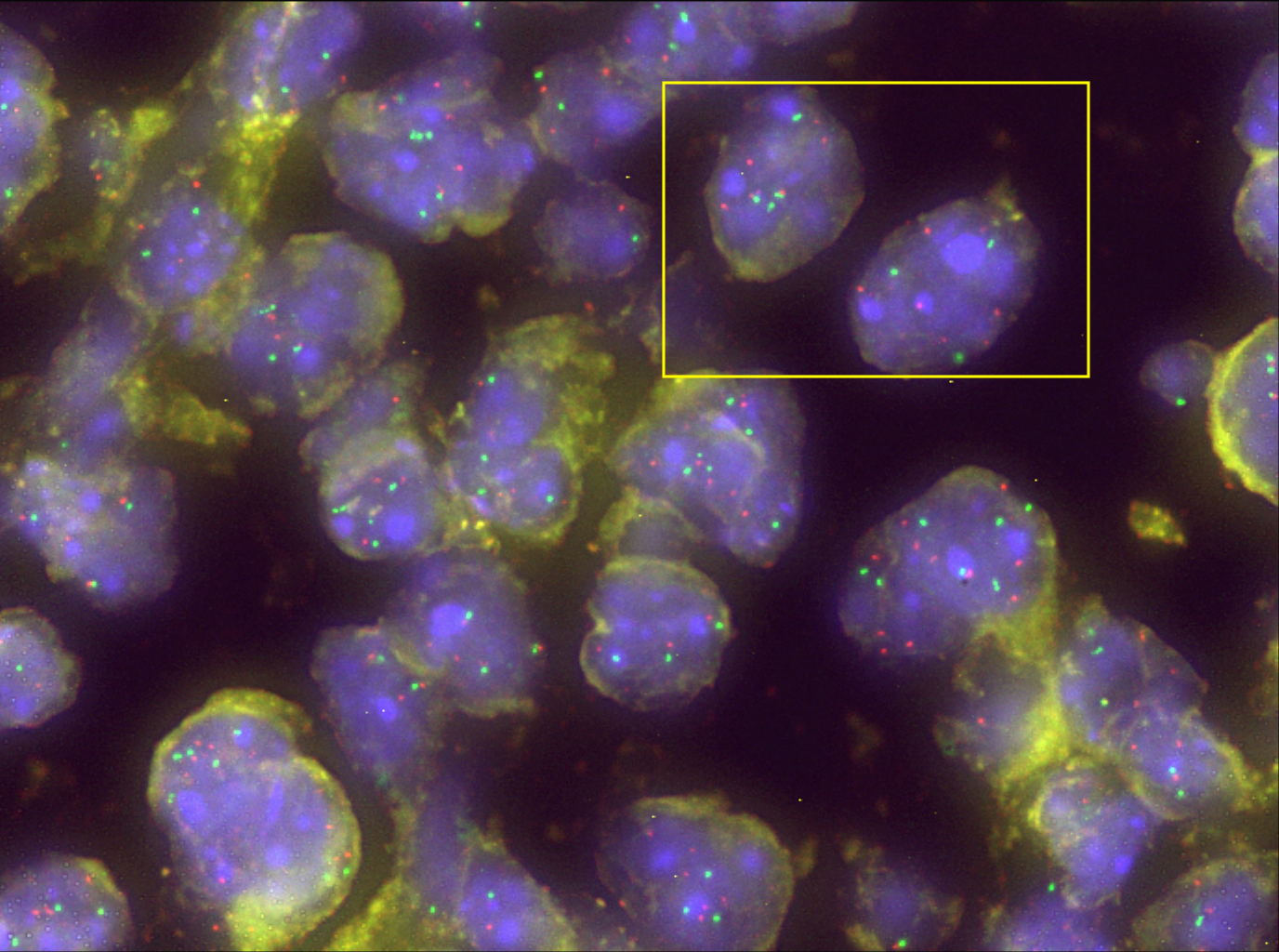

Supplement: Supplementary file 8 — Source data Fig. 2 [file 44321_2025_326_MOESM8_ESM.zip › Fig2/Fig2a/Kras FISH - 7 months.tif]

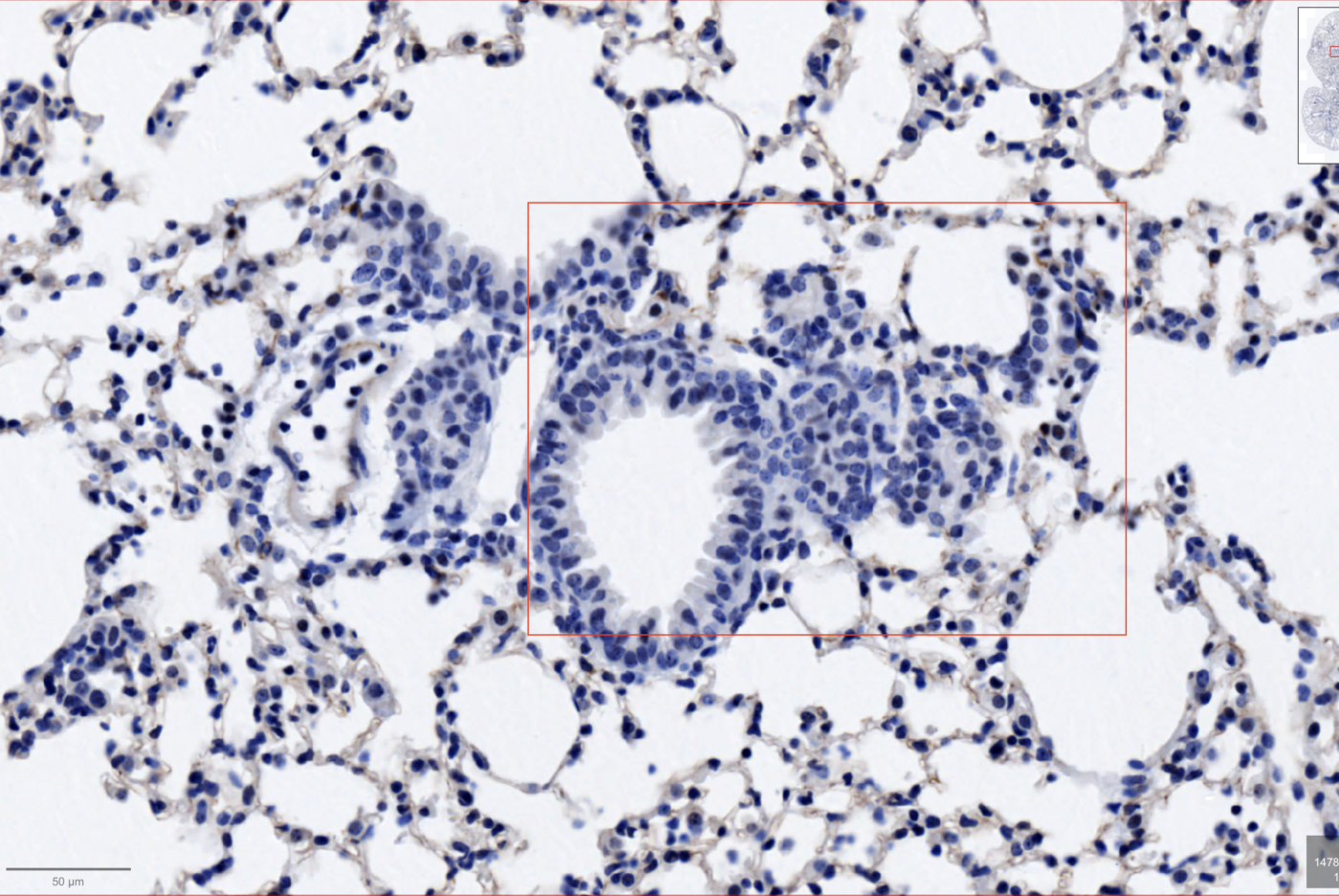

Supplement: Supplementary file 8 — Source data Fig. 2 [file 44321_2025_326_MOESM8_ESM.zip › Fig2/Fig2a/ETV5 - 1 month.tif]

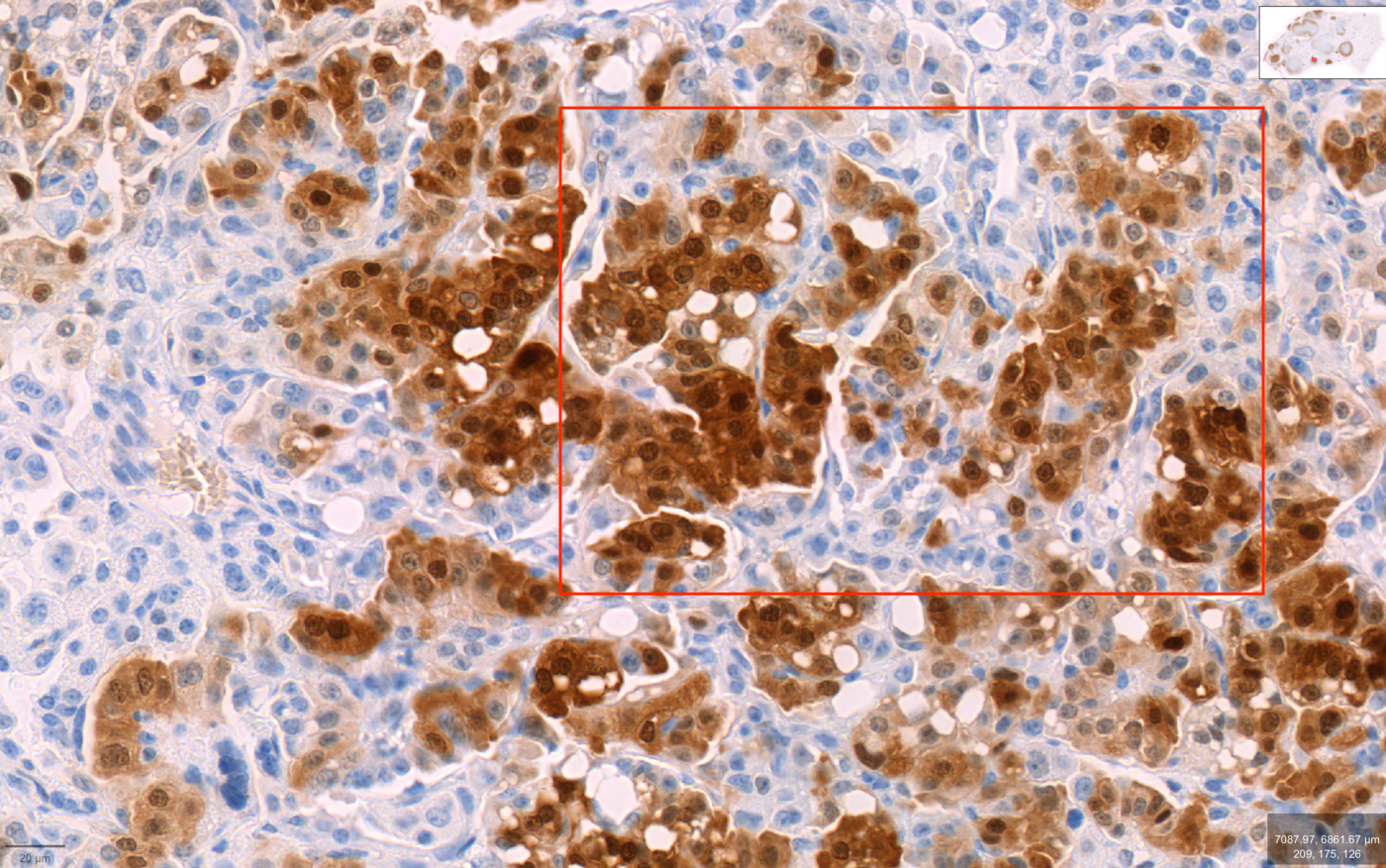

Supplement: Supplementary file 8 — Source data Fig. 2 [file 44321_2025_326_MOESM8_ESM.zip › Fig2/Fig2a/pERK - 3 months.tif]

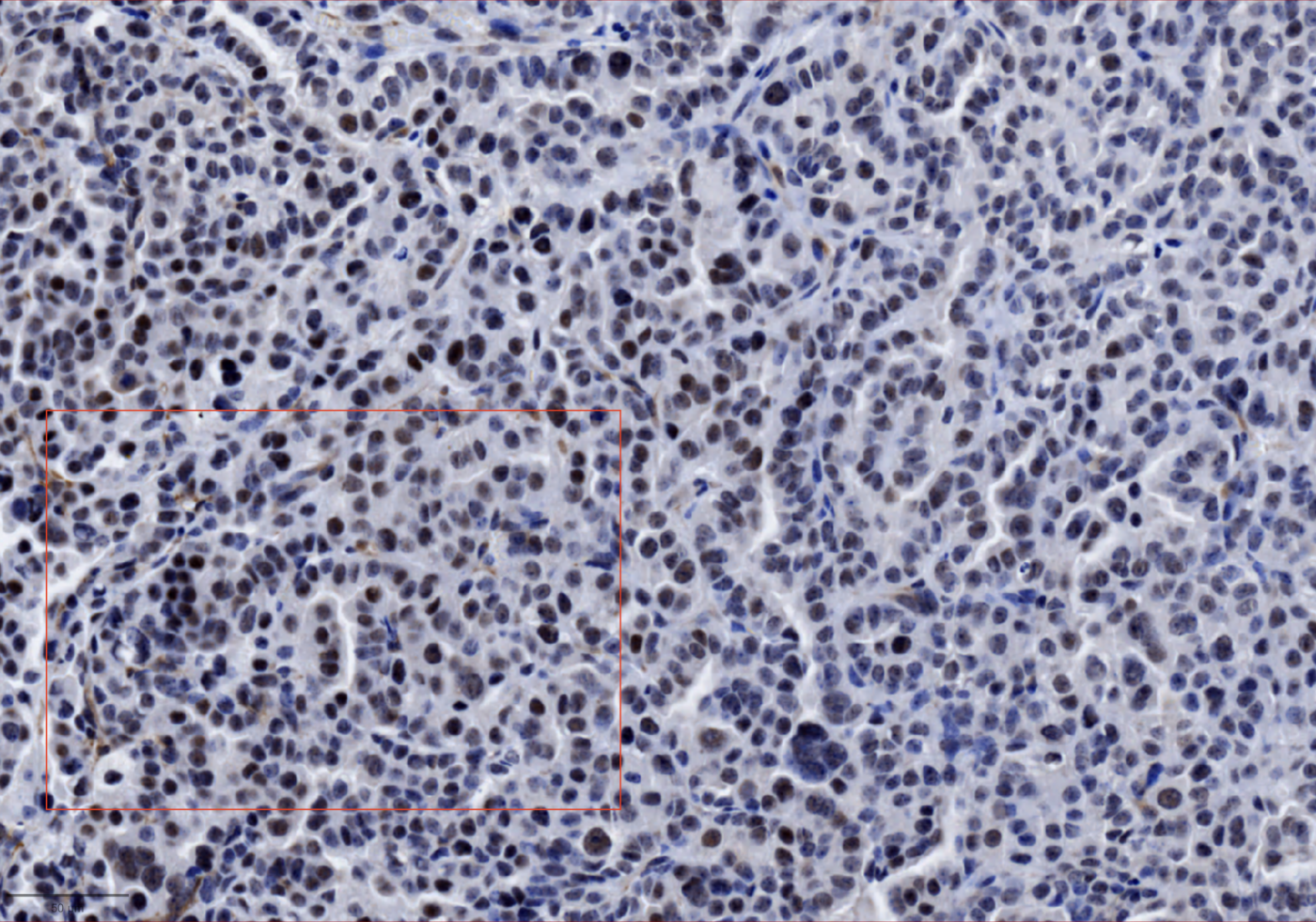

Supplement: Supplementary file 8 — Source data Fig. 2 [file 44321_2025_326_MOESM8_ESM.zip › Fig2/Fig2a/ETV5 - 5 months.tif]

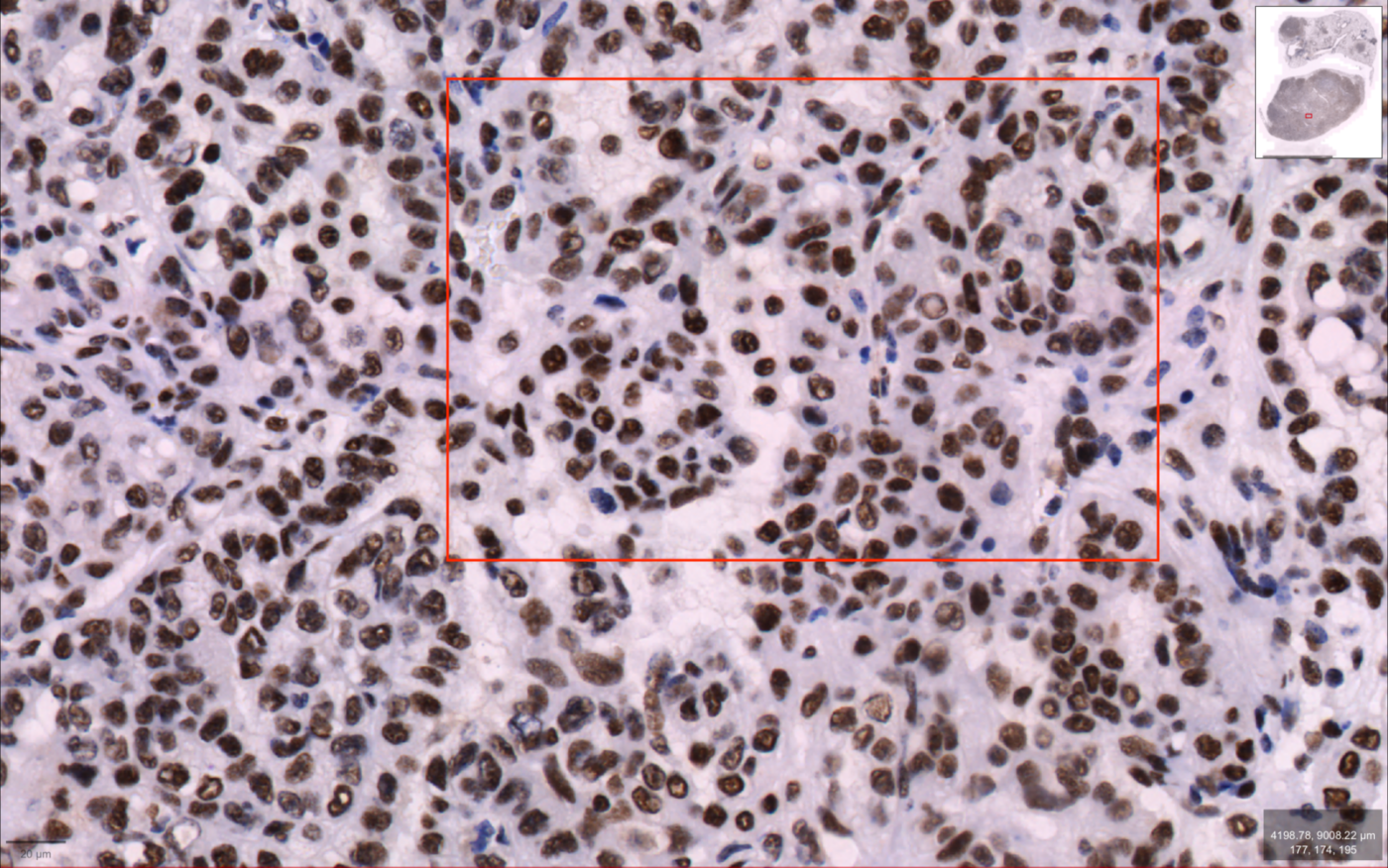

Supplement: Supplementary file 8 — Source data Fig. 2 [file 44321_2025_326_MOESM8_ESM.zip › Fig2/Fig2a/CIC - 7 months.tif]

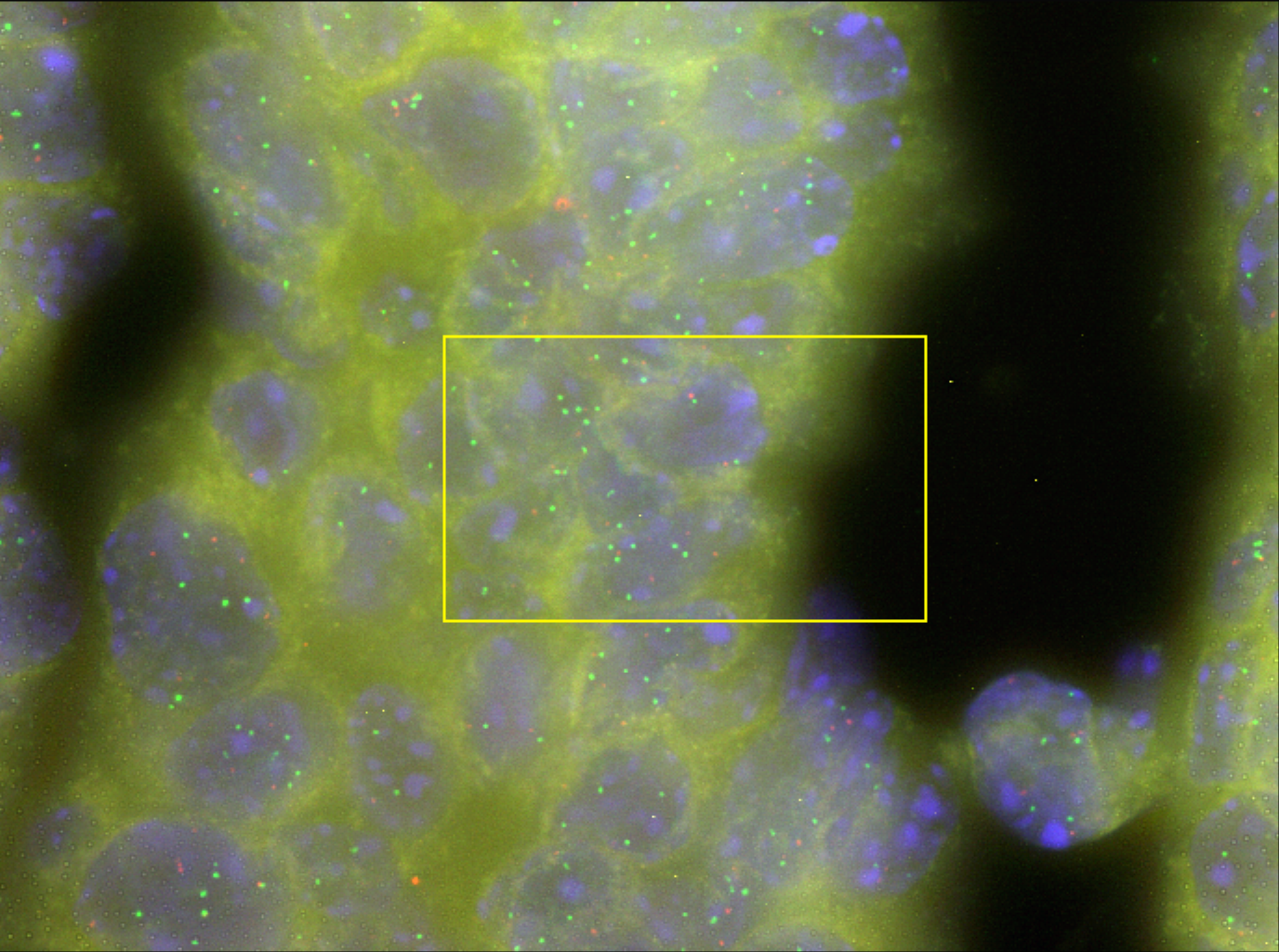

Supplement: Supplementary file 8 — Source data Fig. 2 [file 44321_2025_326_MOESM8_ESM.zip › Fig2/Fig2a/Kras FISH - 3 months.tif]

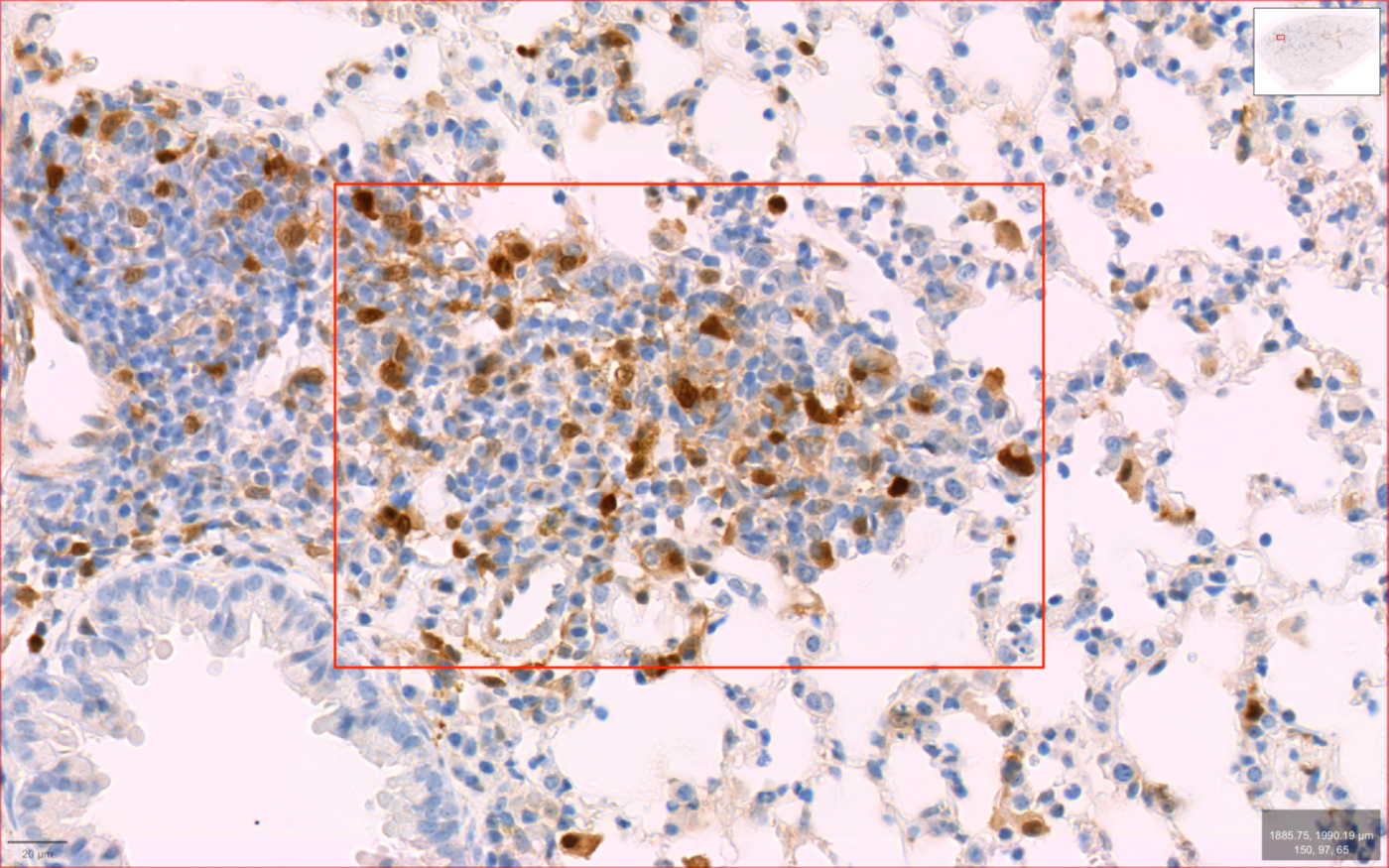

Supplement: Supplementary file 8 — Source data Fig. 2 [file 44321_2025_326_MOESM8_ESM.zip › Fig2/Fig2a/pERK - 1 month.tif]

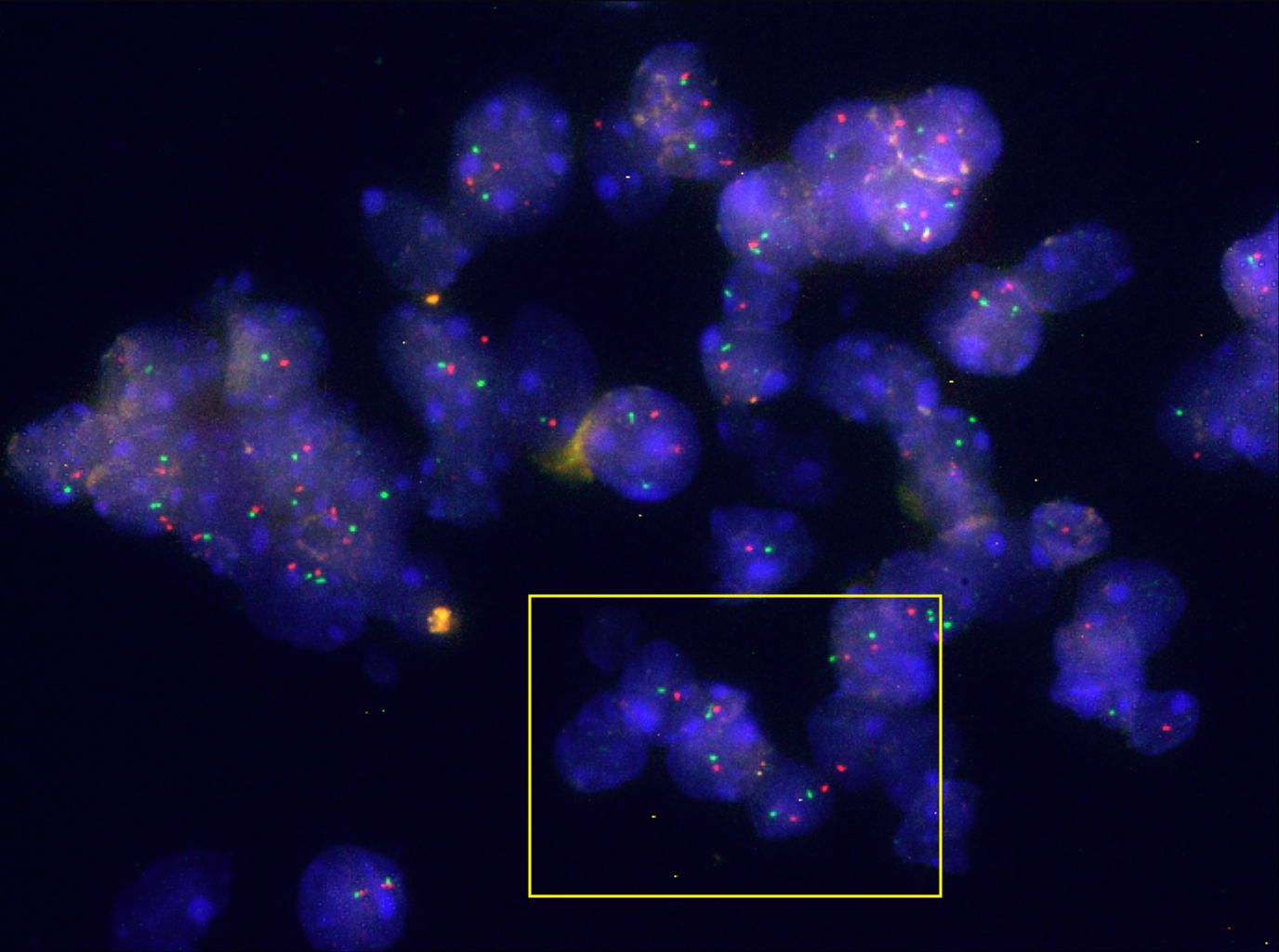

Supplement: Supplementary file 8 — Source data Fig. 2 [file 44321_2025_326_MOESM8_ESM.zip › Fig2/Fig2a/Kras FISH - 1 month.tif]

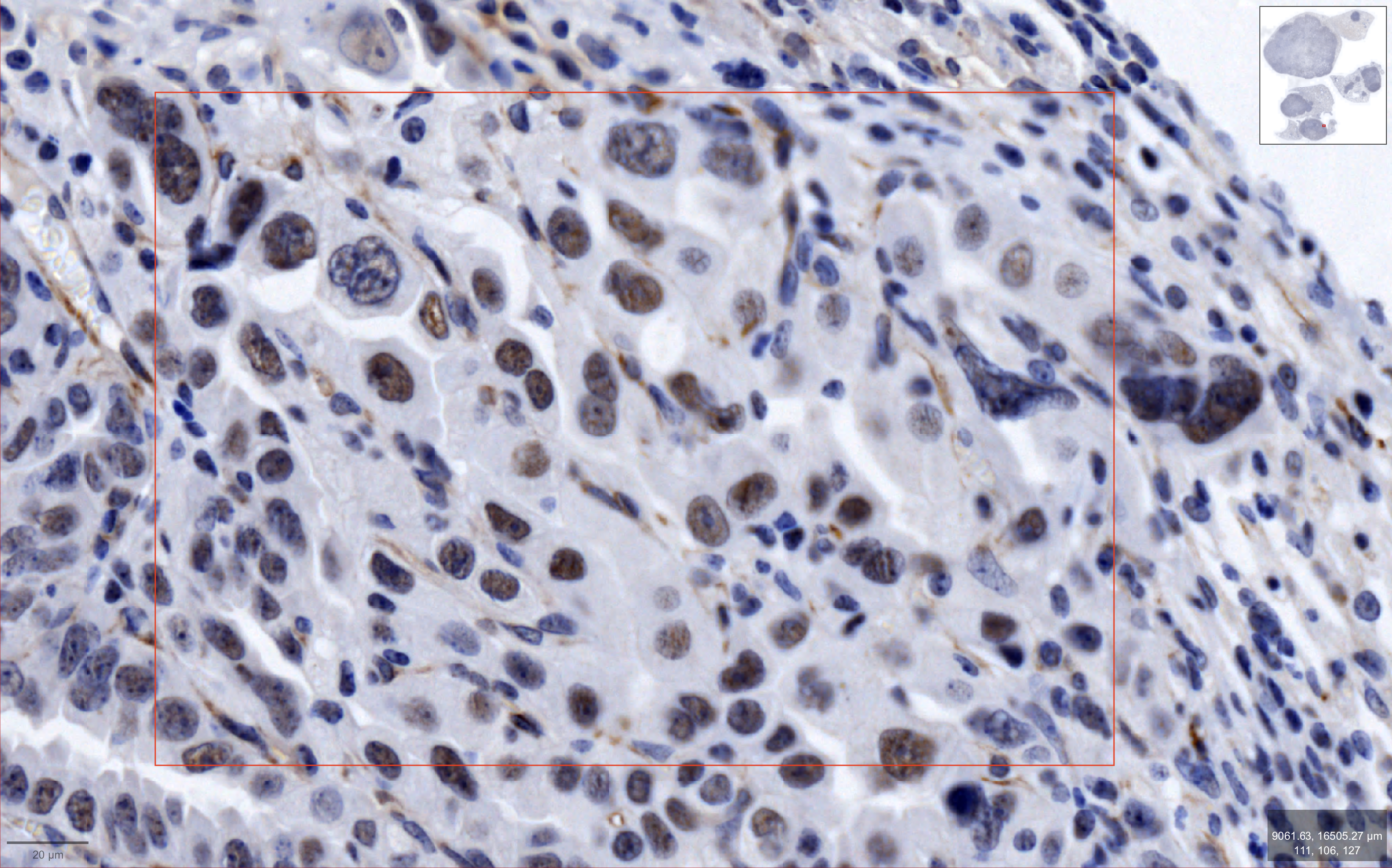

Supplement: Supplementary file 8 — Source data Fig. 2 [file 44321_2025_326_MOESM8_ESM.zip › Fig2/Fig2a/ETV5 - 7 months.tif]

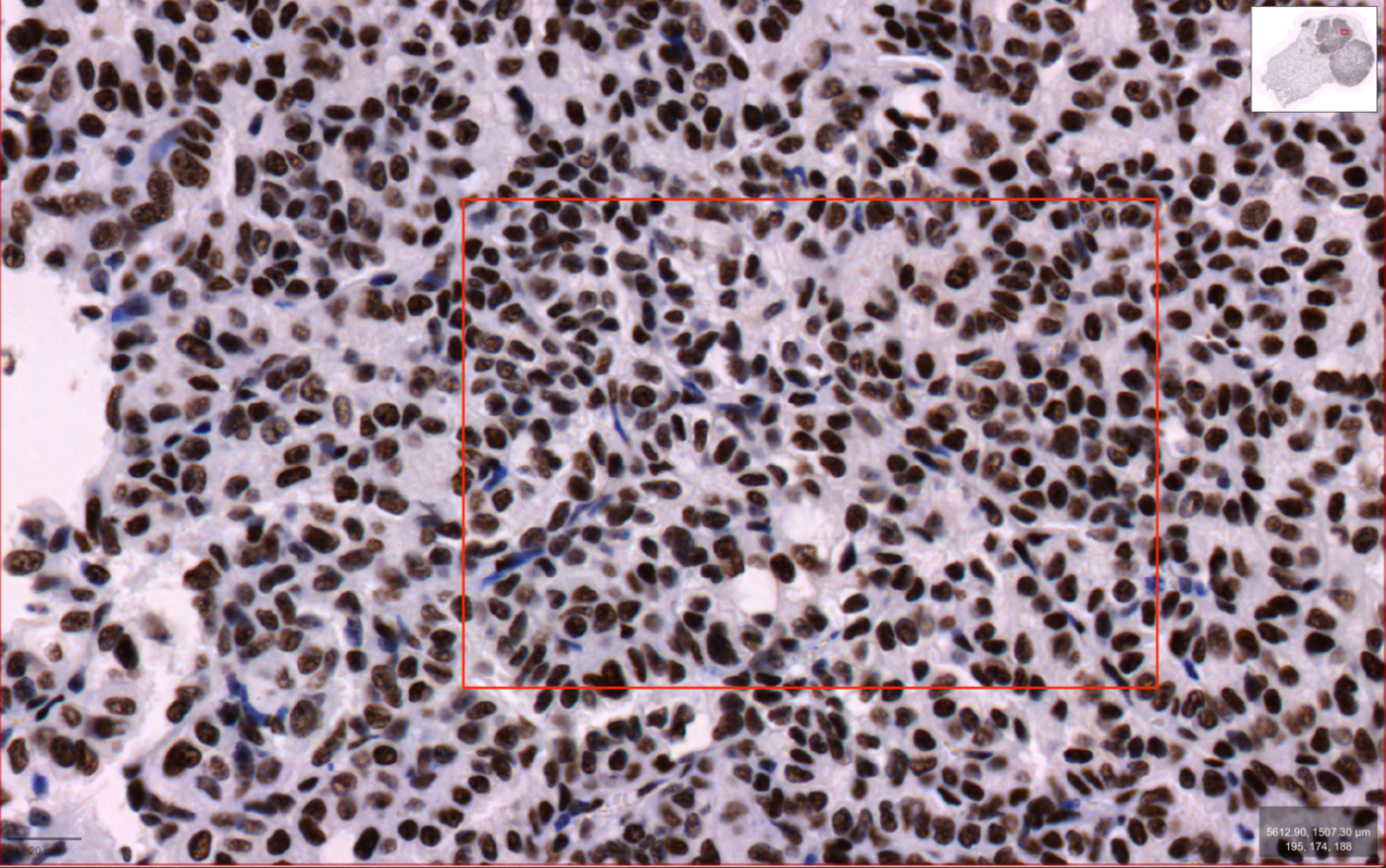

Supplement: Supplementary file 8 — Source data Fig. 2 [file 44321_2025_326_MOESM8_ESM.zip › Fig2/Fig2a/CIC - 5 months.tif]

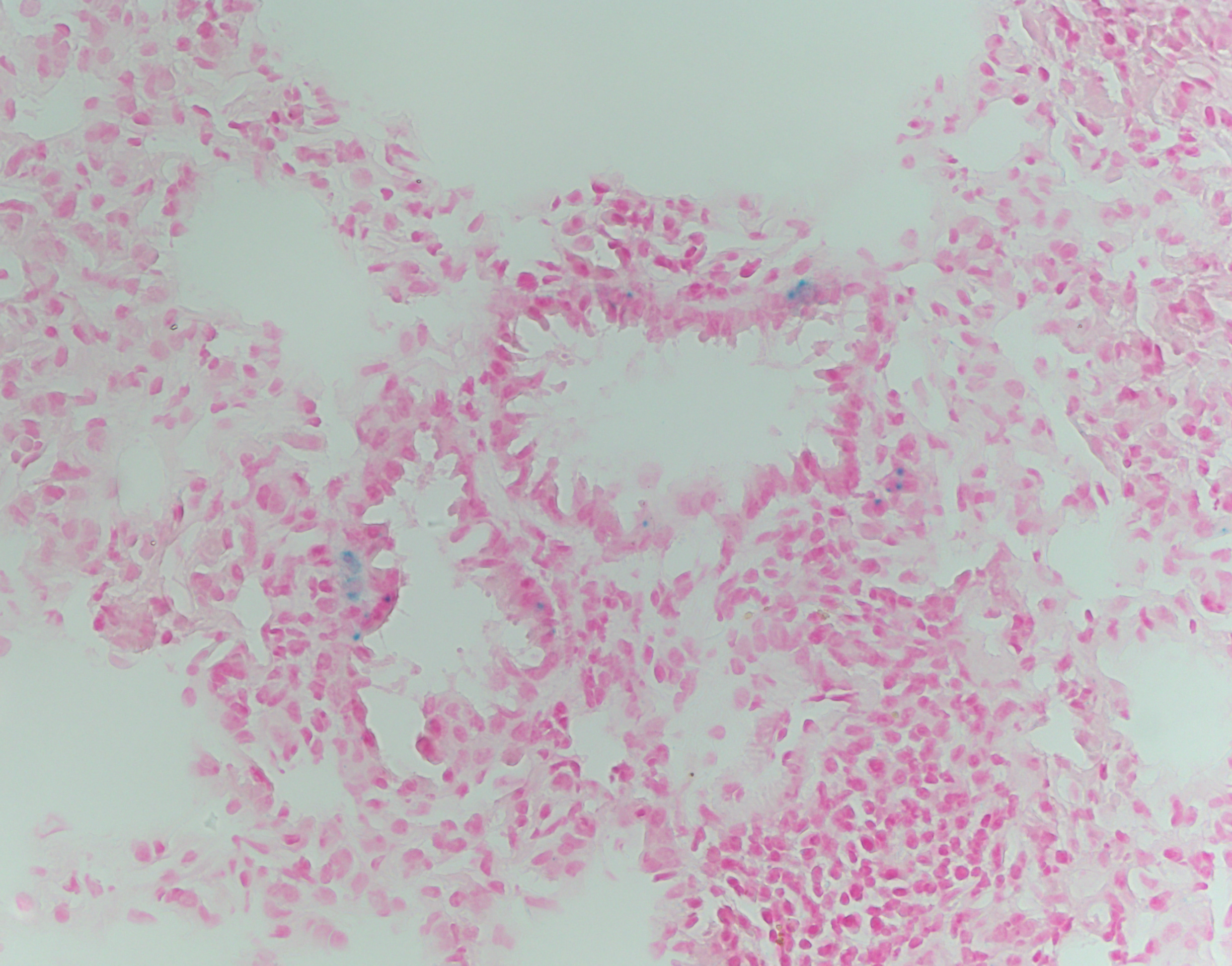

Supplement: Supplementary file 9 — Source data Fig. 3 [file 44321_2025_326_MOESM9_ESM.zip › Fig3/Fig3a/KP.tif]

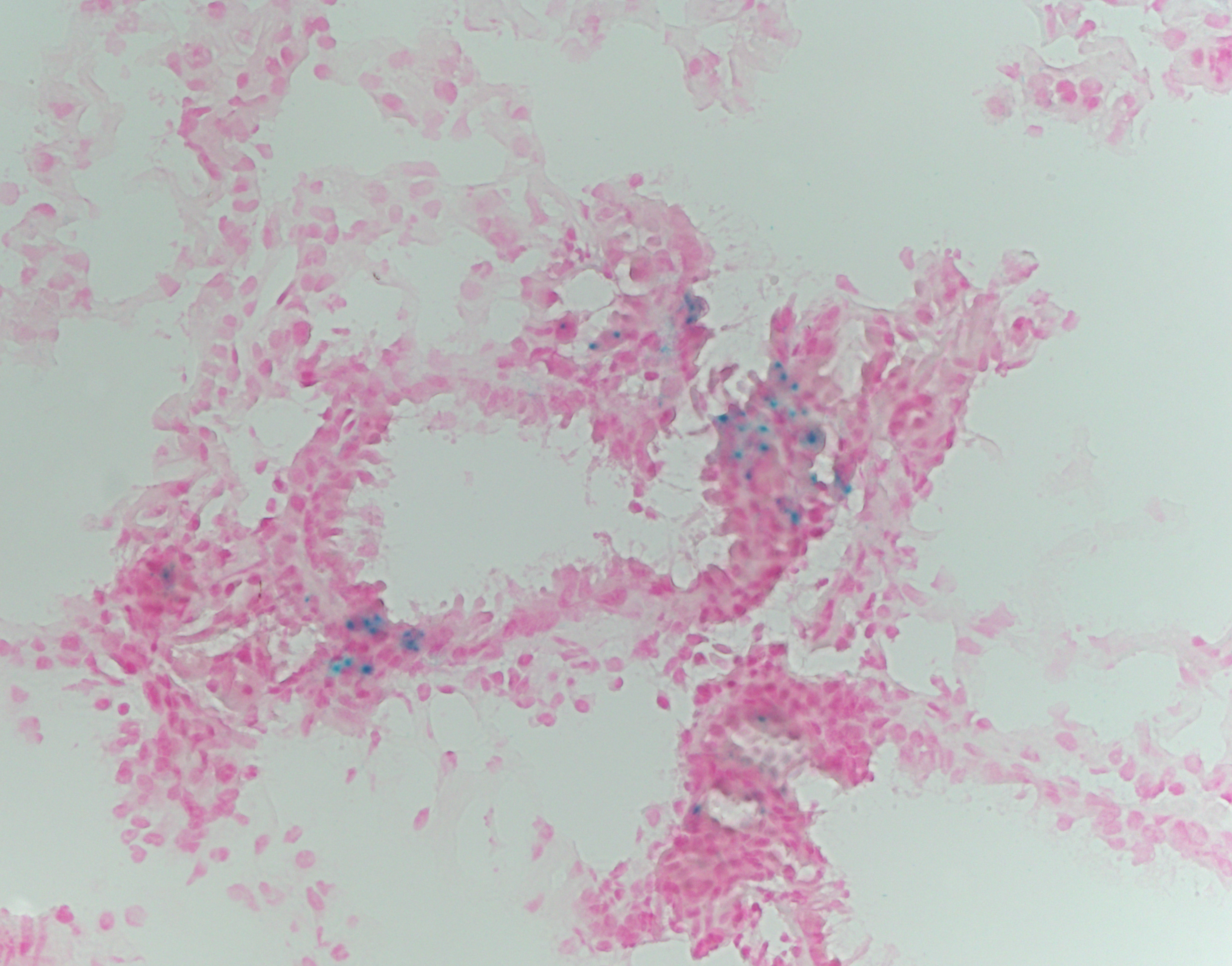

Supplement: Supplementary file 9 — Source data Fig. 3 [file 44321_2025_326_MOESM9_ESM.zip › Fig3/Fig3a/KPCic.tif]

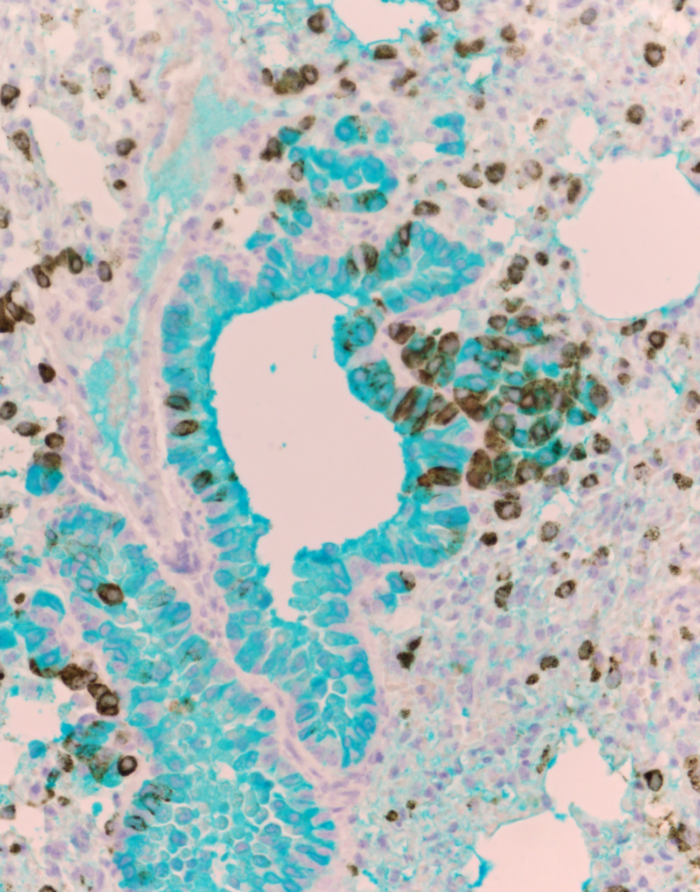

Supplement: Supplementary file 9 — Source data Fig. 3 [file 44321_2025_326_MOESM9_ESM.zip › Fig3/Fig3c/KP - 20 weeks.tif]

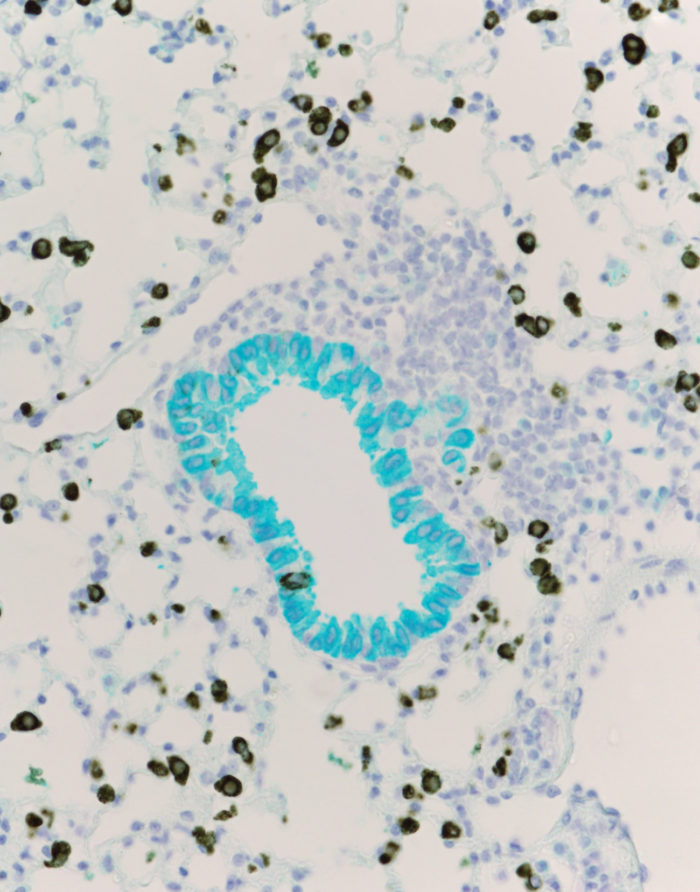

Supplement: Supplementary file 9 — Source data Fig. 3 [file 44321_2025_326_MOESM9_ESM.zip › Fig3/Fig3c/KP - 4 weeks.tif]

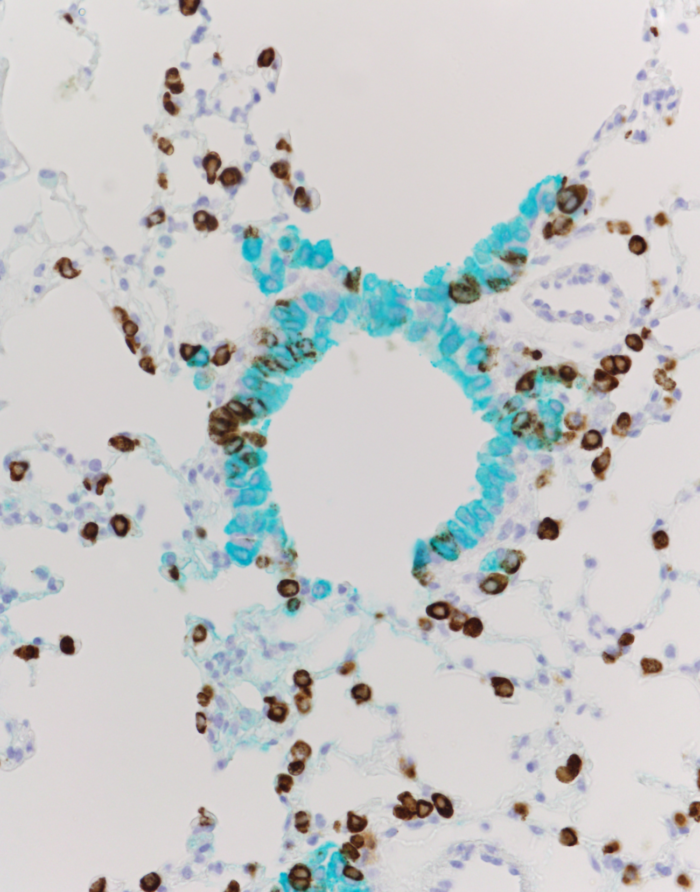

Supplement: Supplementary file 9 — Source data Fig. 3 [file 44321_2025_326_MOESM9_ESM.zip › Fig3/Fig3c/KPCic - 4 weeks.tif]

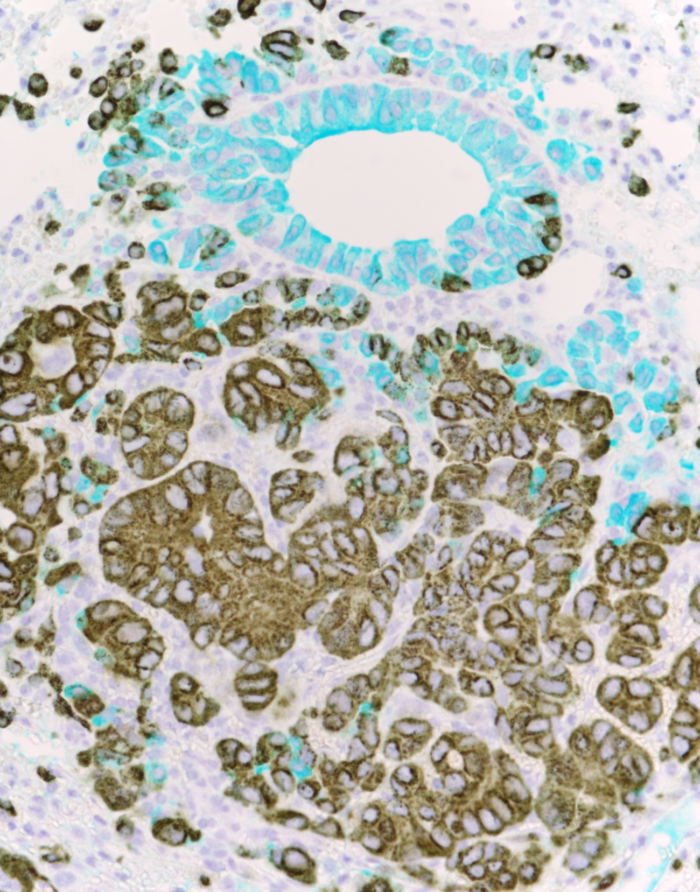

Supplement: Supplementary file 9 — Source data Fig. 3 [file 44321_2025_326_MOESM9_ESM.zip › Fig3/Fig3c/KPCic - 20 weeks.tif]

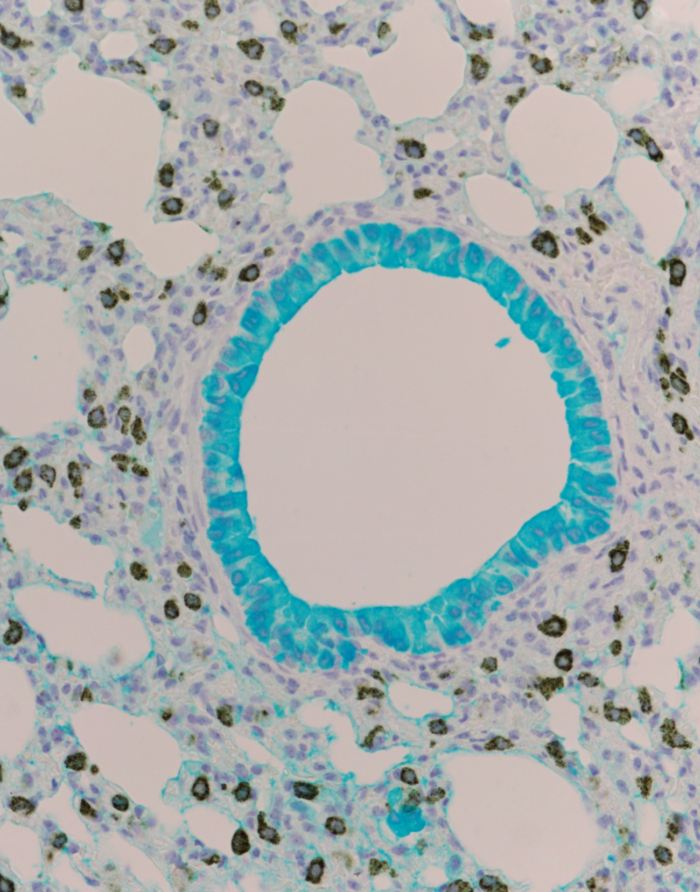

Supplement: Supplementary file 9 — Source data Fig. 3 [file 44321_2025_326_MOESM9_ESM.zip › Fig3/Fig3c/KP - not infected.tif]

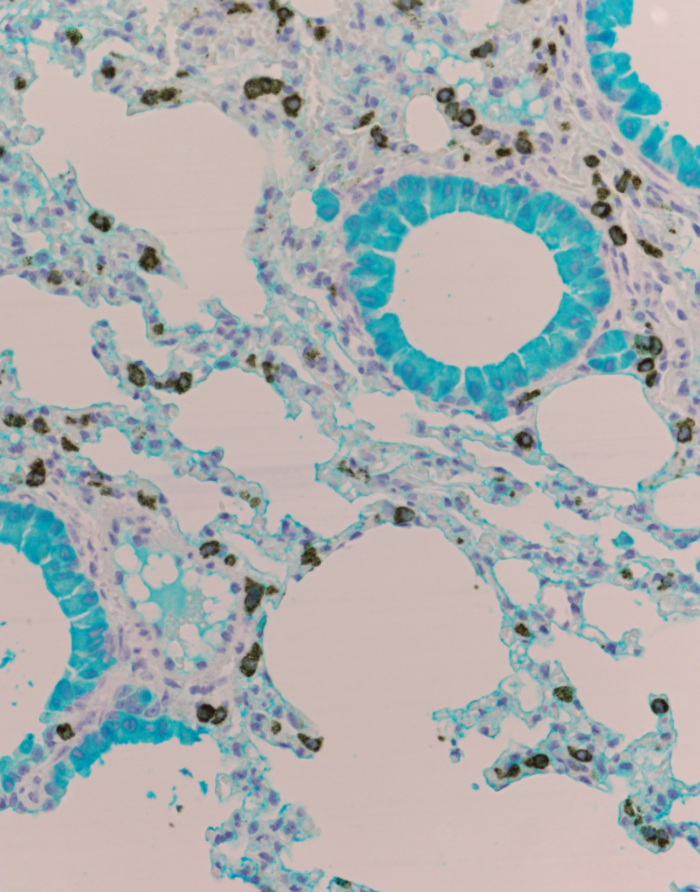

Supplement: Supplementary file 9 — Source data Fig. 3 [file 44321_2025_326_MOESM9_ESM.zip › Fig3/Fig3c/KPCic - not infected.tif]

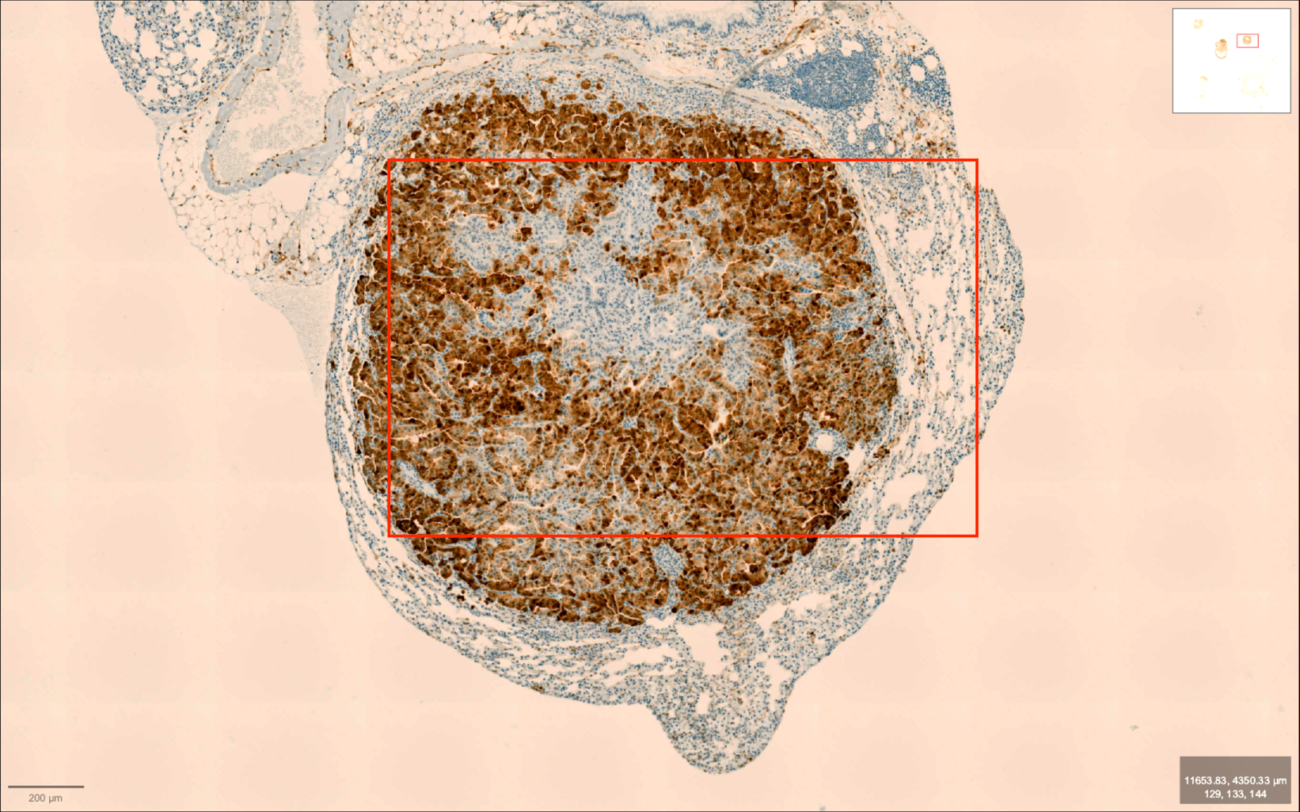

Supplement: Supplementary file 10 — Source data Fig. 4 [file 44321_2025_326_MOESM10_ESM.zip › Fig4/Fig4f/KP.tif]

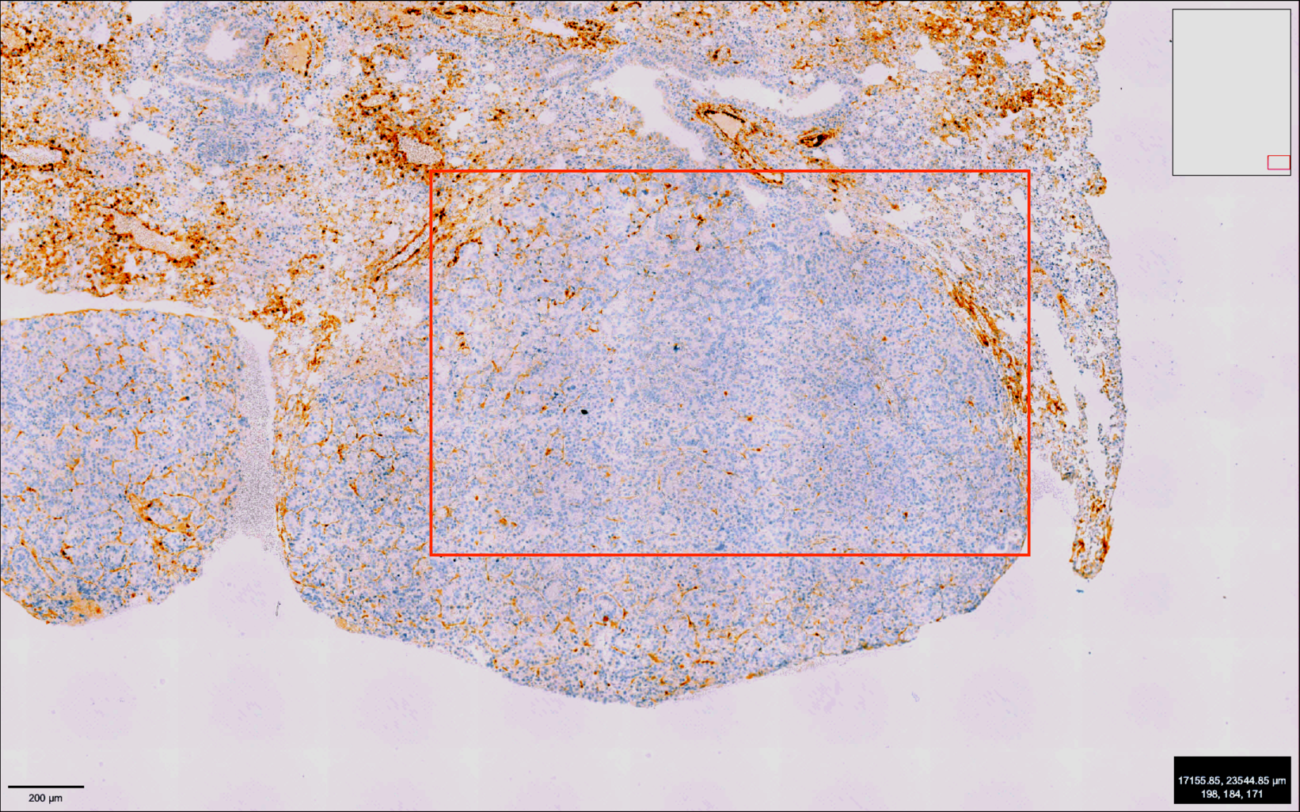

Supplement: Supplementary file 10 — Source data Fig. 4 [file 44321_2025_326_MOESM10_ESM.zip › Fig4/Fig4f/PCic.tif]

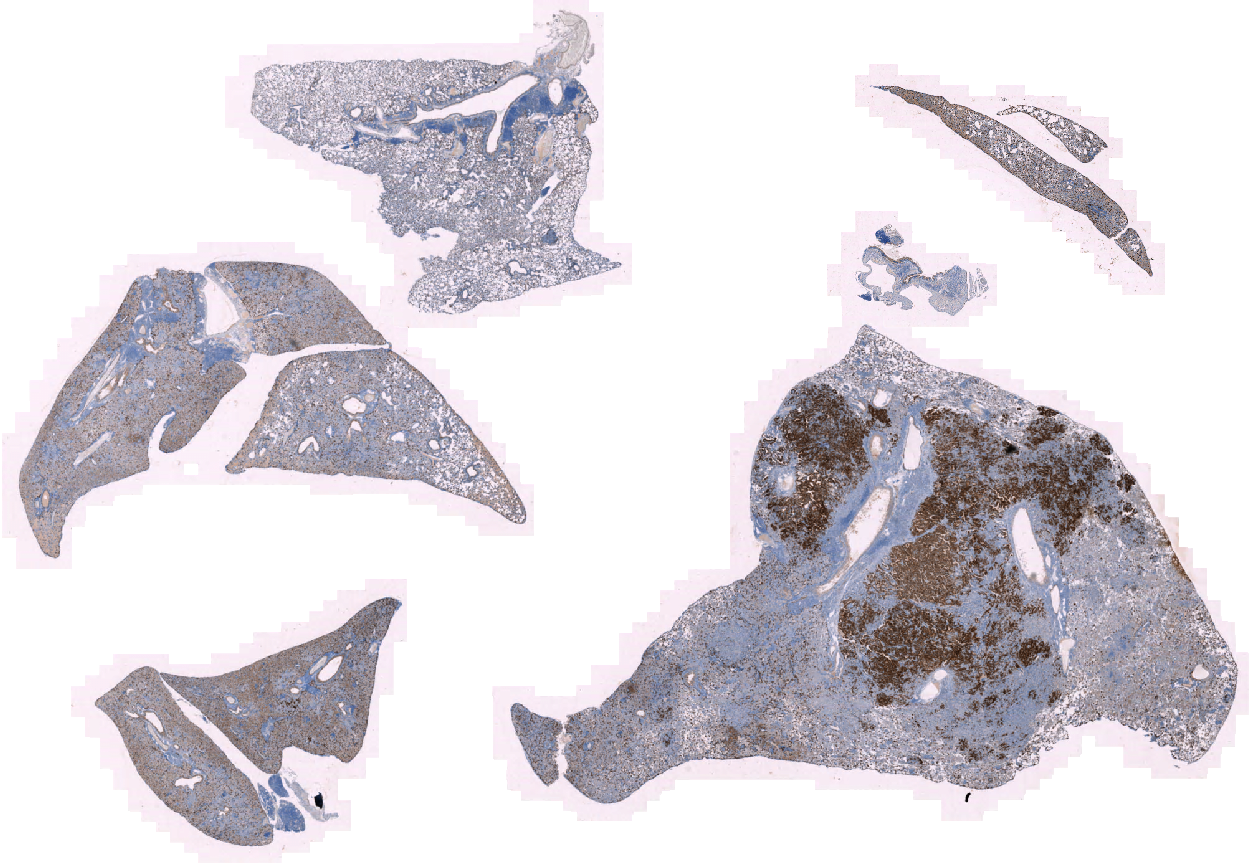

Supplement: Supplementary file 10 — Source data Fig. 4 [file 44321_2025_326_MOESM10_ESM.zip › Fig4/Fig4d/PCic SPC.tif]

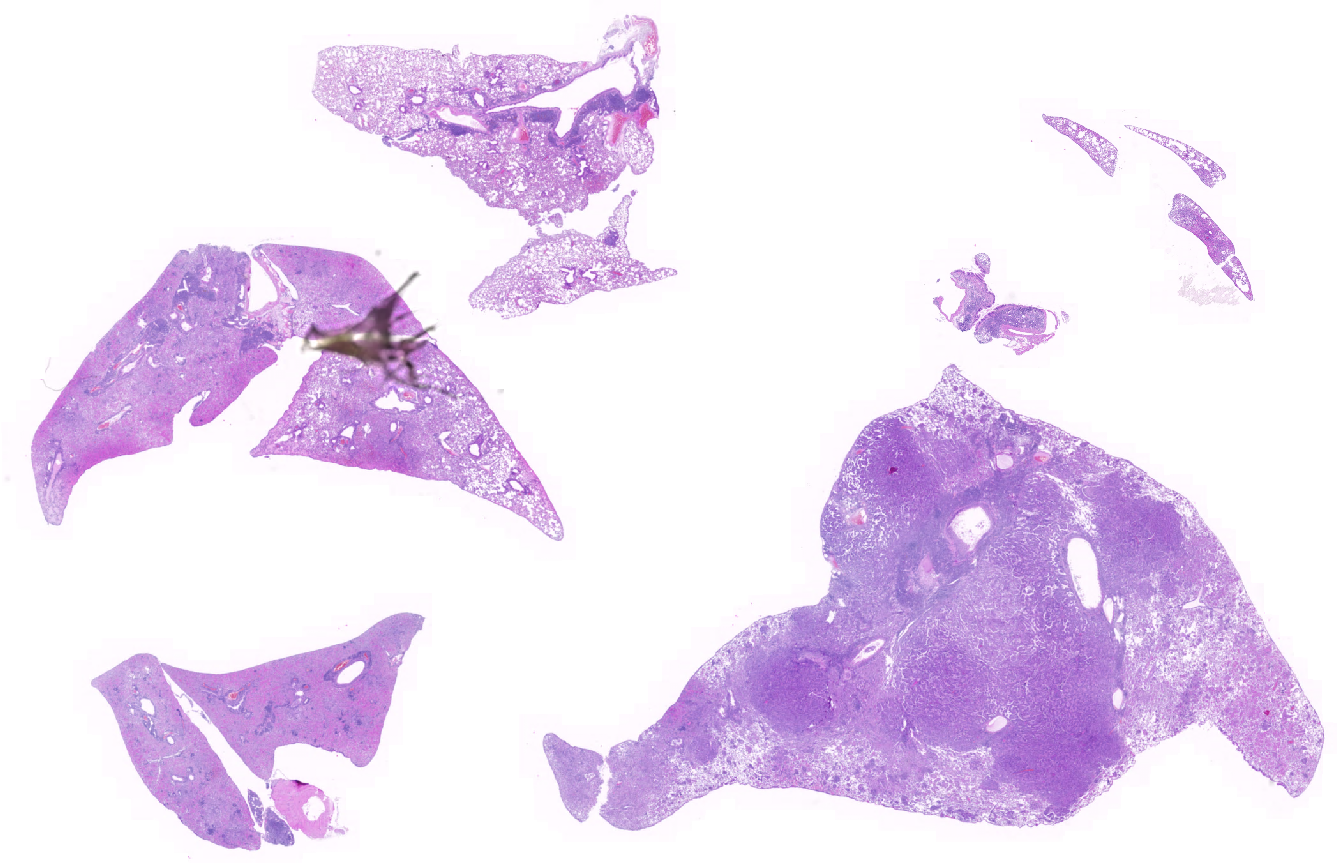

Supplement: Supplementary file 10 — Source data Fig. 4 [file 44321_2025_326_MOESM10_ESM.zip › Fig4/Fig4d/PCic HE.tif]

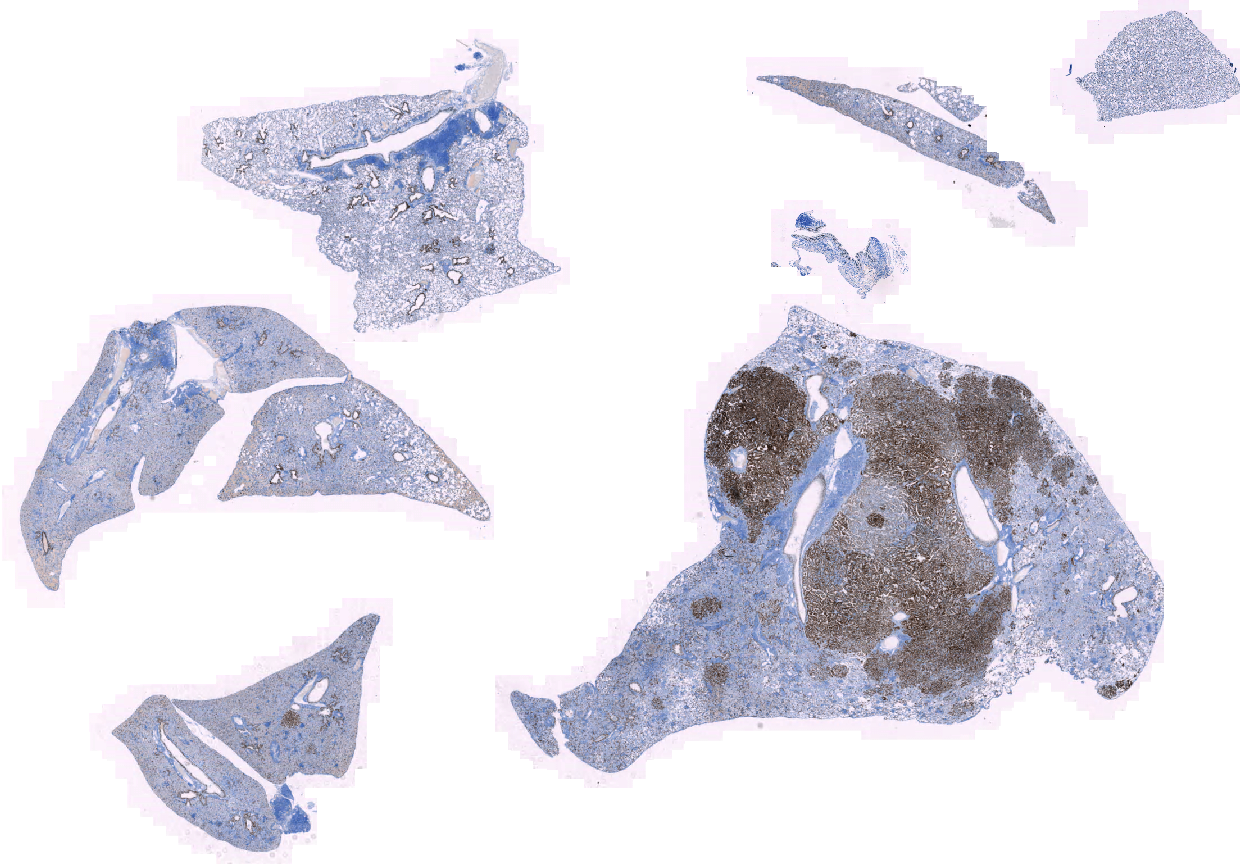

Supplement: Supplementary file 10 — Source data Fig. 4 [file 44321_2025_326_MOESM10_ESM.zip › Fig4/Fig4d/PCic TTF1.tif]

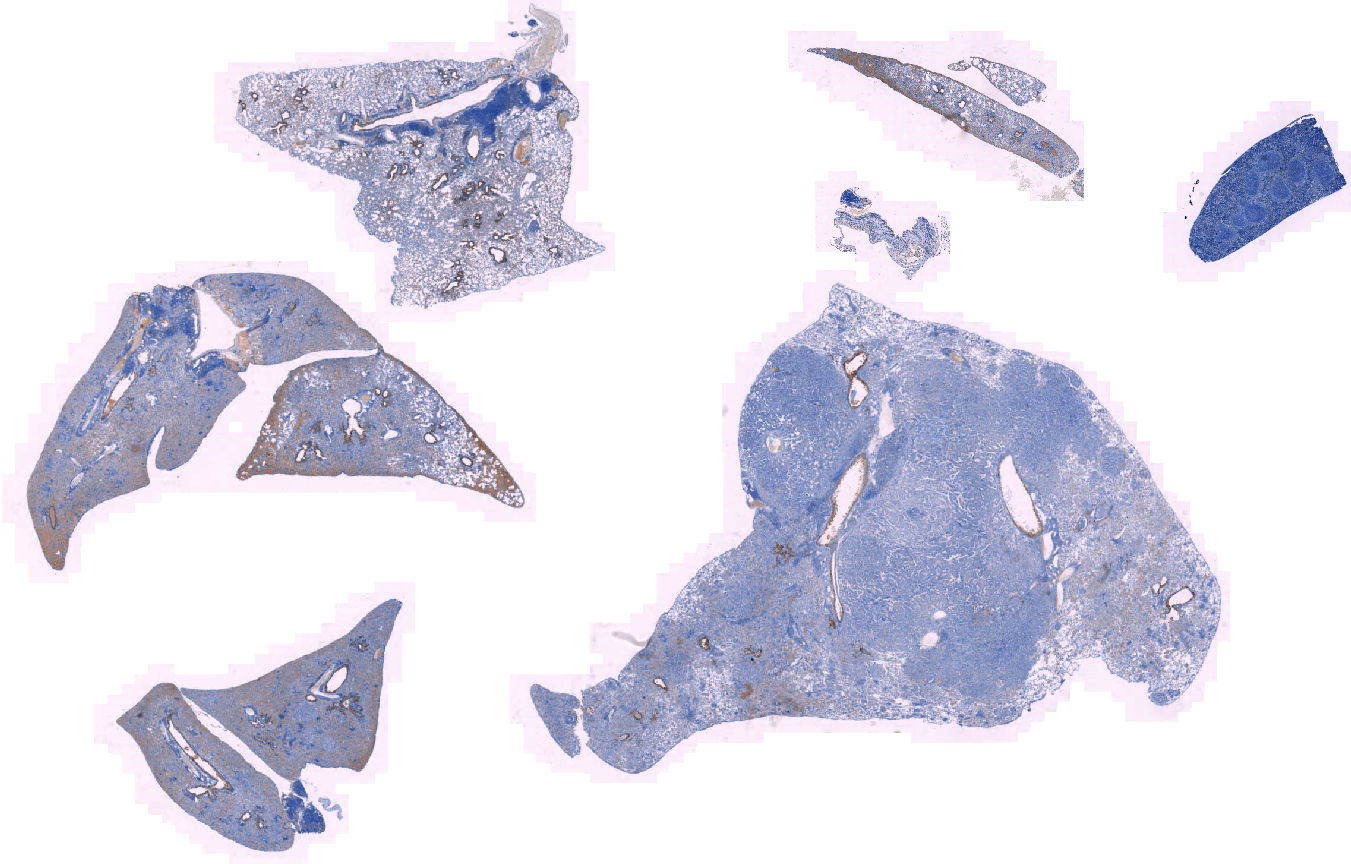

Supplement: Supplementary file 10 — Source data Fig. 4 [file 44321_2025_326_MOESM10_ESM.zip › Fig4/Fig4d/PCic CC10.tif]

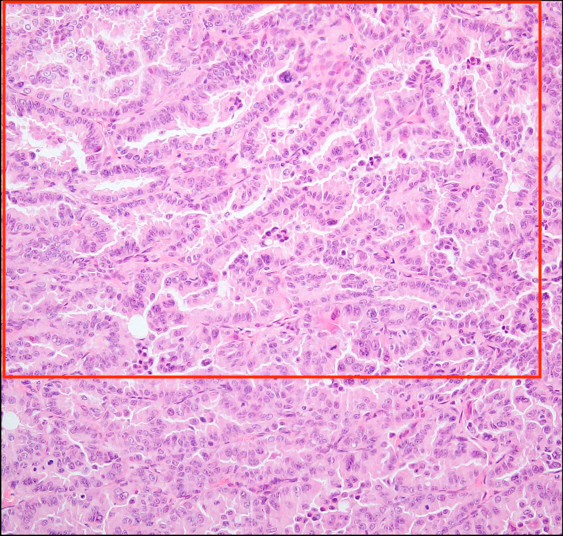

Supplement: Supplementary file 10 — Source data Fig. 4 [file 44321_2025_326_MOESM10_ESM.zip › Fig4/Fig4c/pCic.tif]

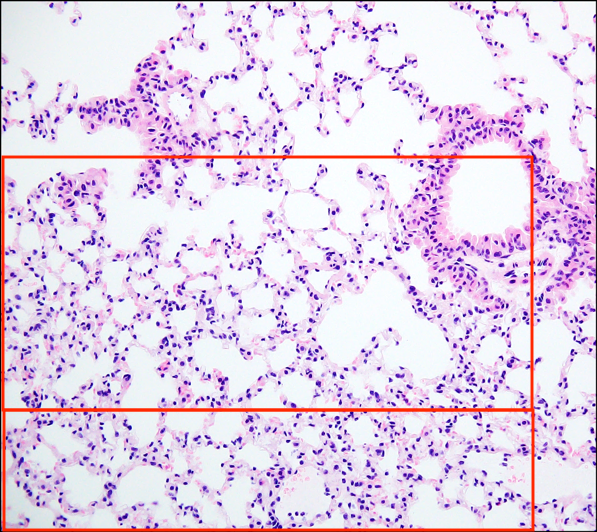

Supplement: Supplementary file 10 — Source data Fig. 4 [file 44321_2025_326_MOESM10_ESM.zip › Fig4/Fig4c/Cic.tif]

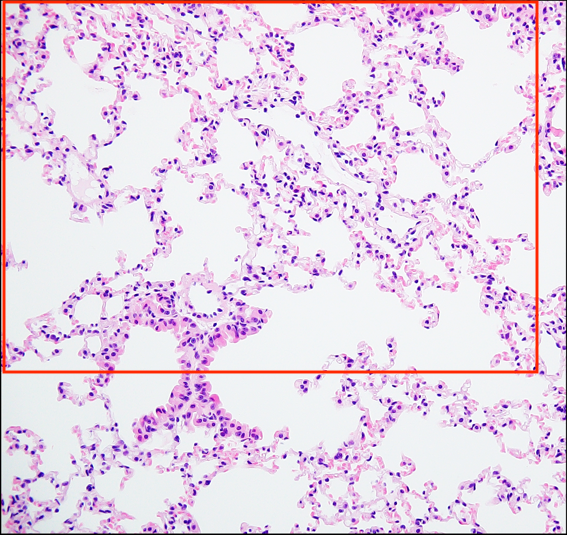

Supplement: Supplementary file 10 — Source data Fig. 4 [file 44321_2025_326_MOESM10_ESM.zip › Fig4/Fig4c/p53.tif]

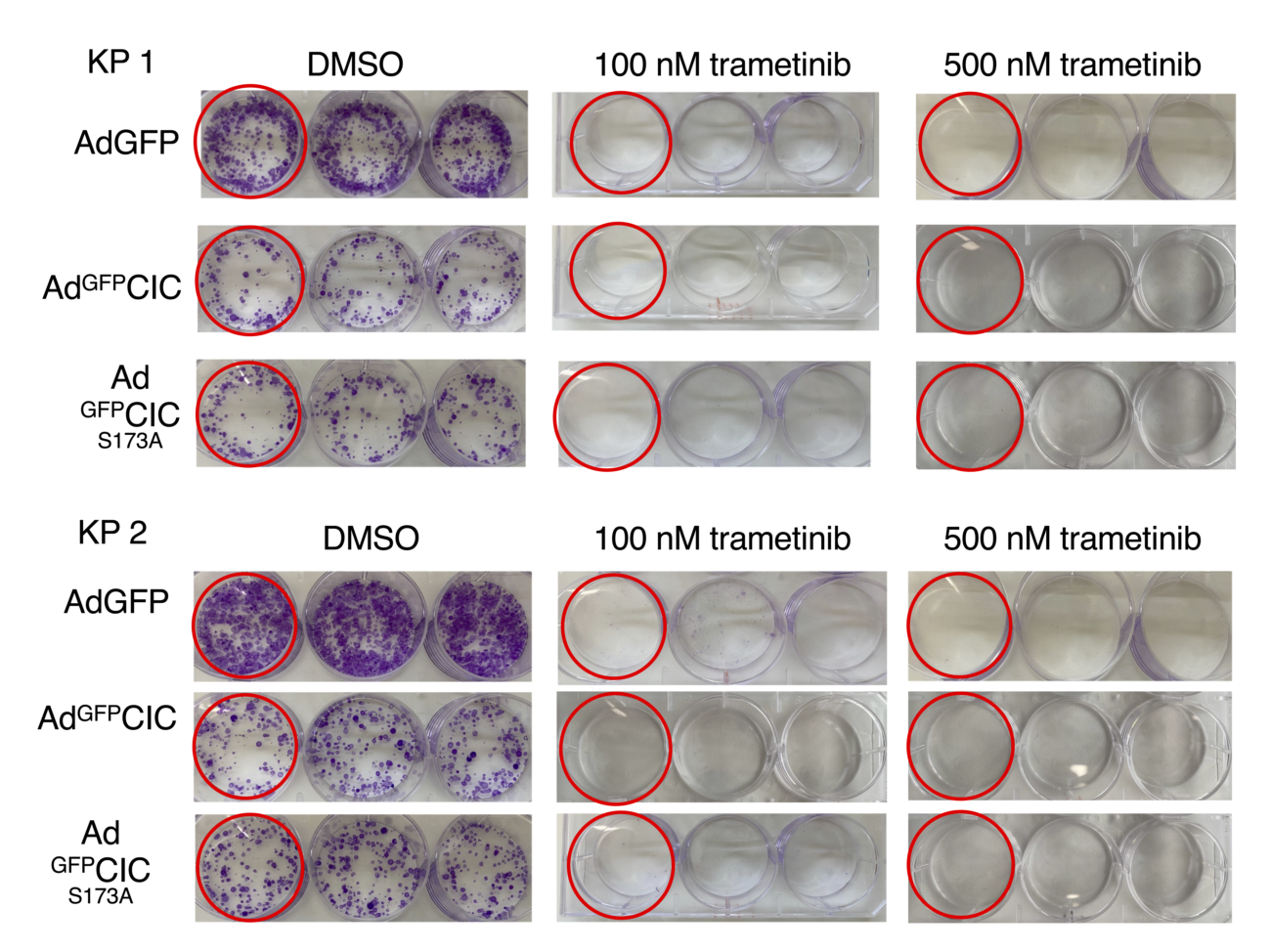

Supplement: Supplementary file 11 — Source data Fig. 5 [file 44321_2025_326_MOESM11_ESM.zip › Fig5/Fig5e/Fig5e-colonies.tiff]

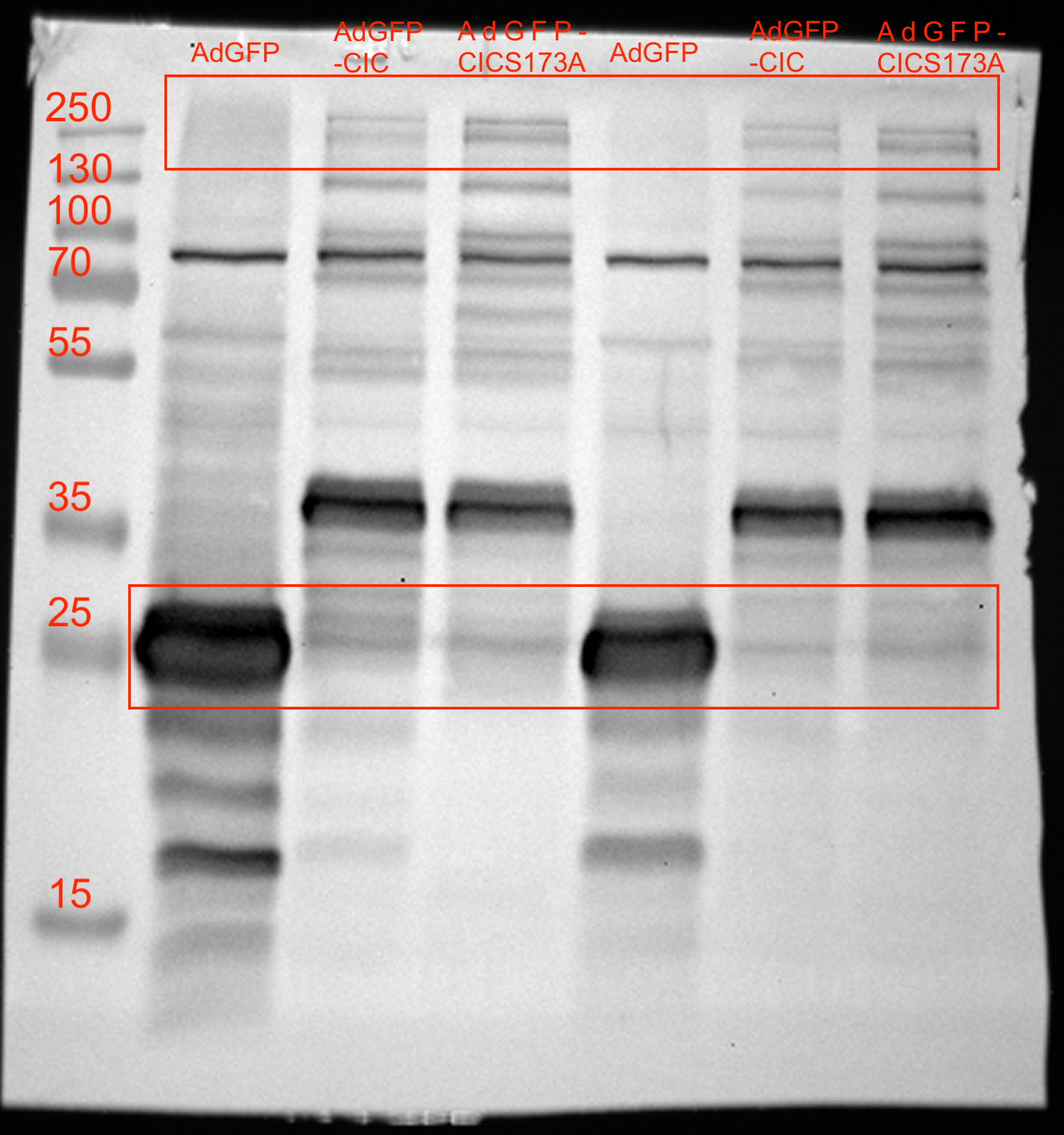

Supplement: Supplementary file 11 — Source data Fig. 5 [file 44321_2025_326_MOESM11_ESM.zip › Fig5/Fig5c/GFP and GFP-CIC (GFP).tif]

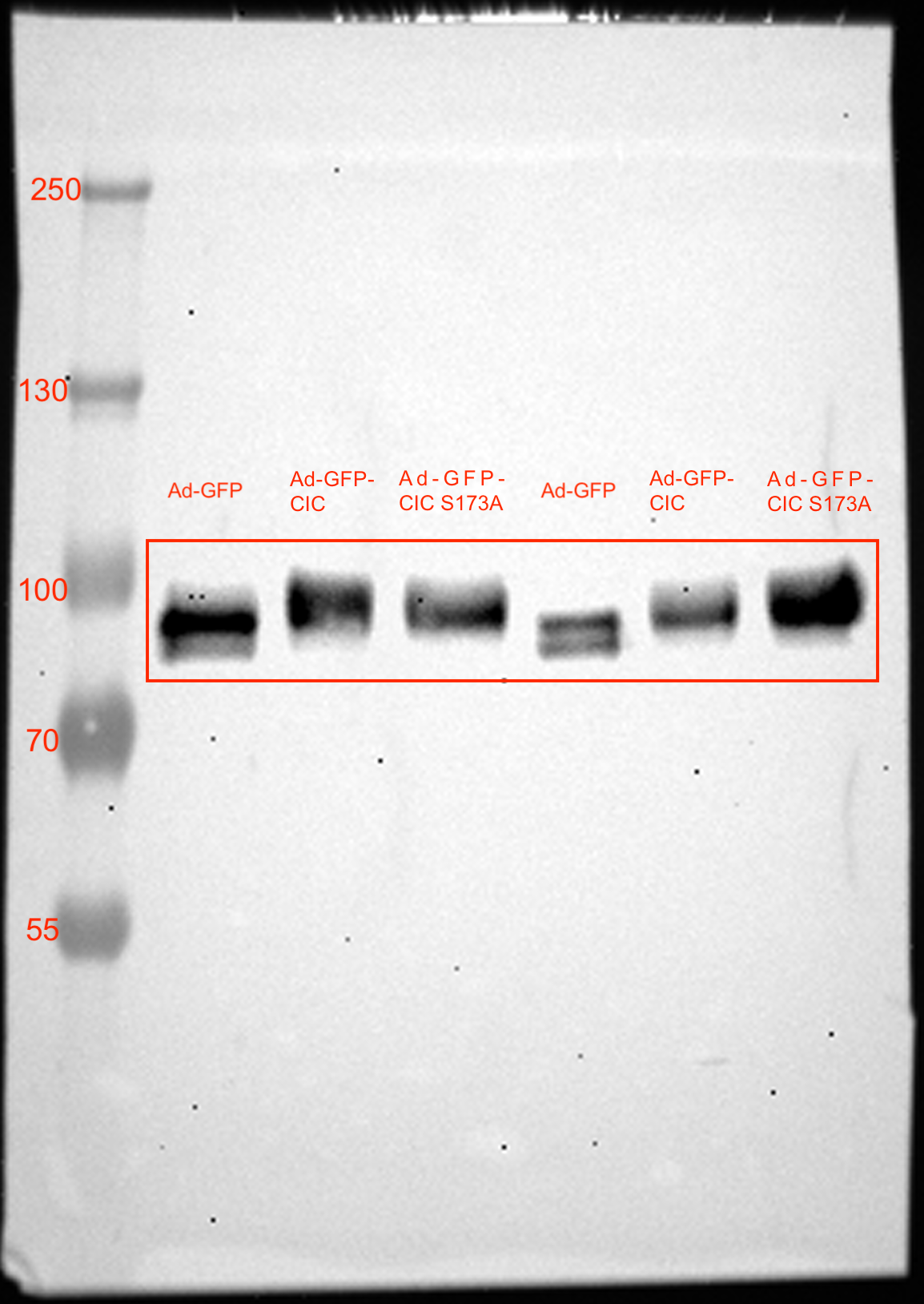

Supplement: Supplementary file 11 — Source data Fig. 5 [file 44321_2025_326_MOESM11_ESM.zip › Fig5/Fig5c/HA-ATXN1L (HA).tif]

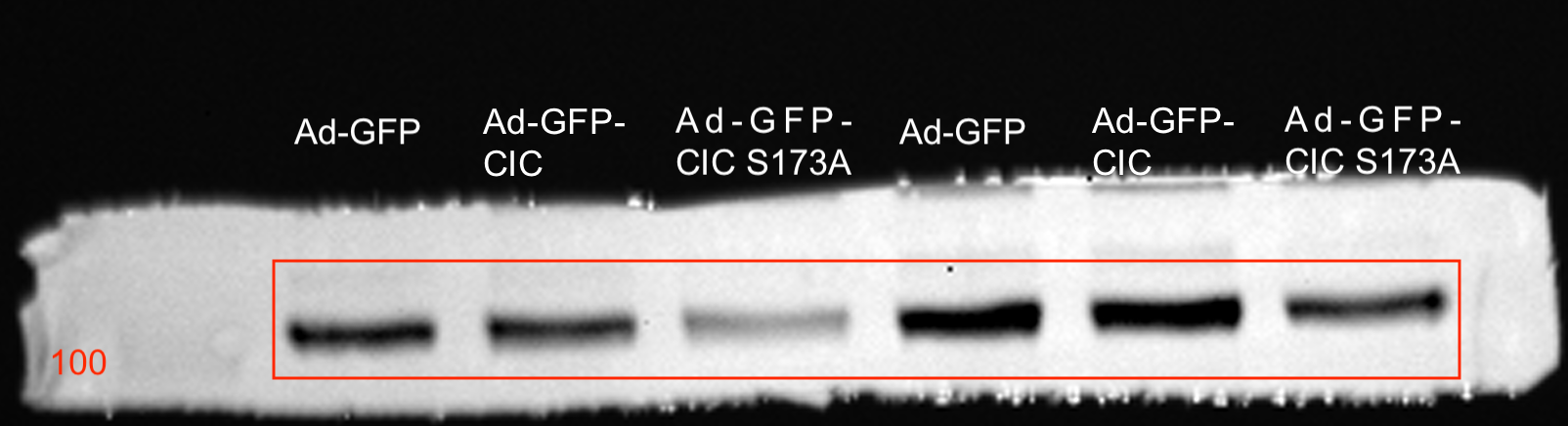

Supplement: Supplementary file 11 — Source data Fig. 5 [file 44321_2025_326_MOESM11_ESM.zip › Fig5/Fig5c/Vinculin.tif]

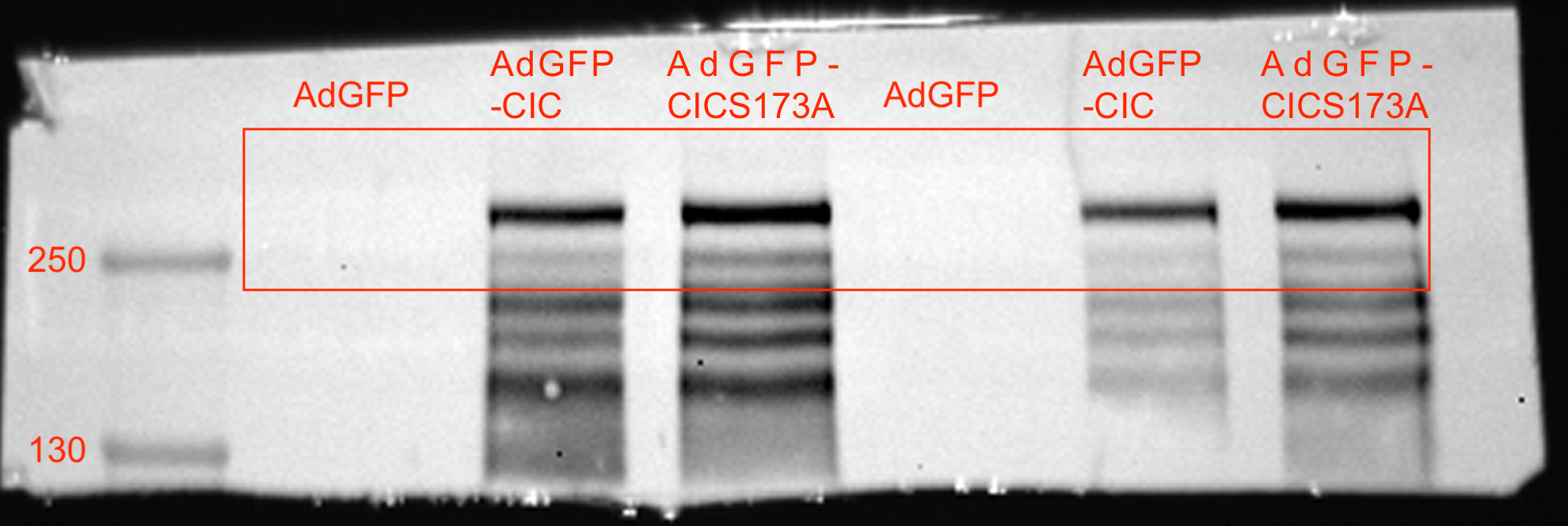

Supplement: Supplementary file 11 — Source data Fig. 5 [file 44321_2025_326_MOESM11_ESM.zip › Fig5/Fig5c/GFP-CIC (CIC).tif]

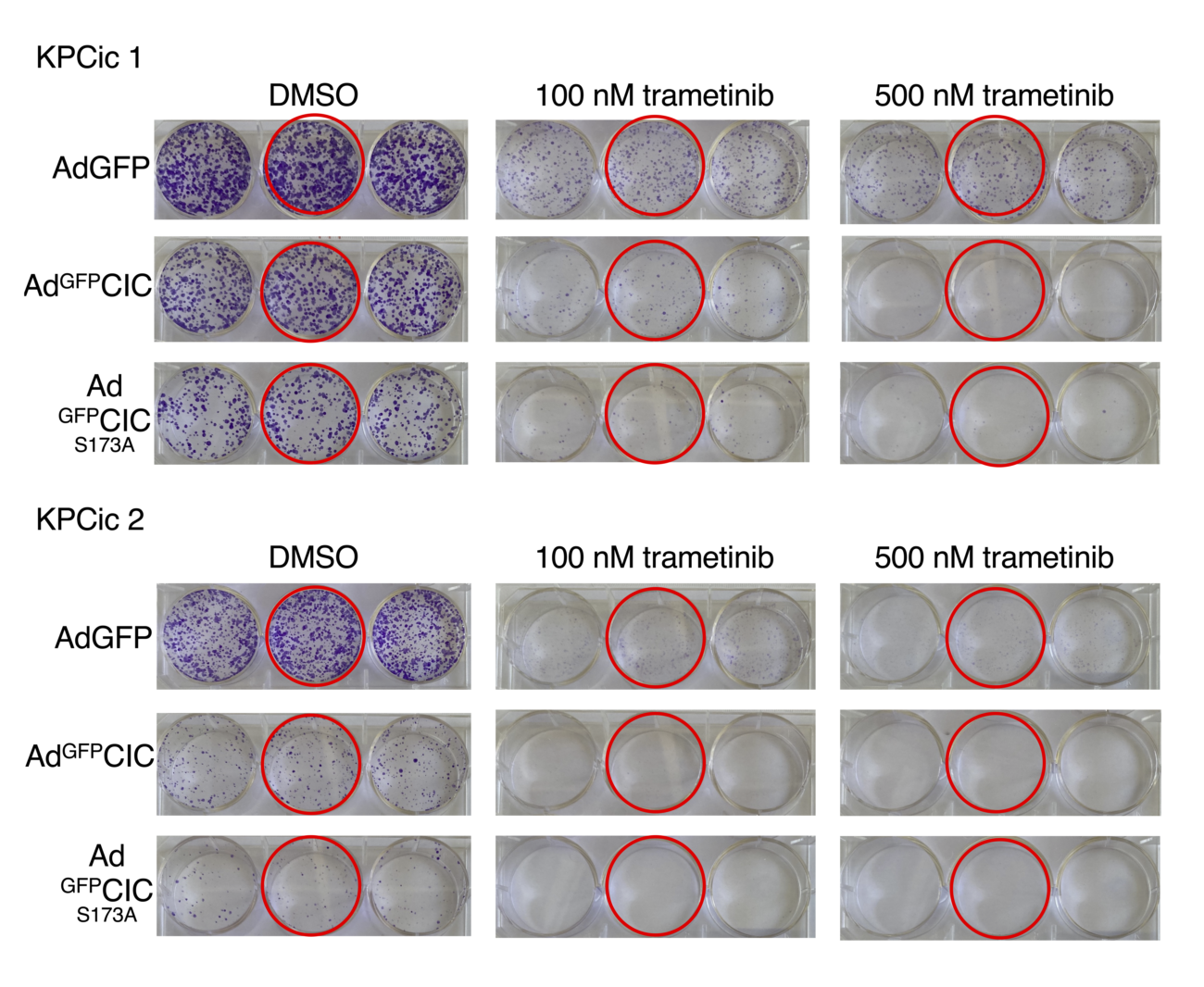

Supplement: Supplementary file 11 — Source data Fig. 5 [file 44321_2025_326_MOESM11_ESM.zip › Fig5/Fig5f/Fig5F-colonies.tiff]

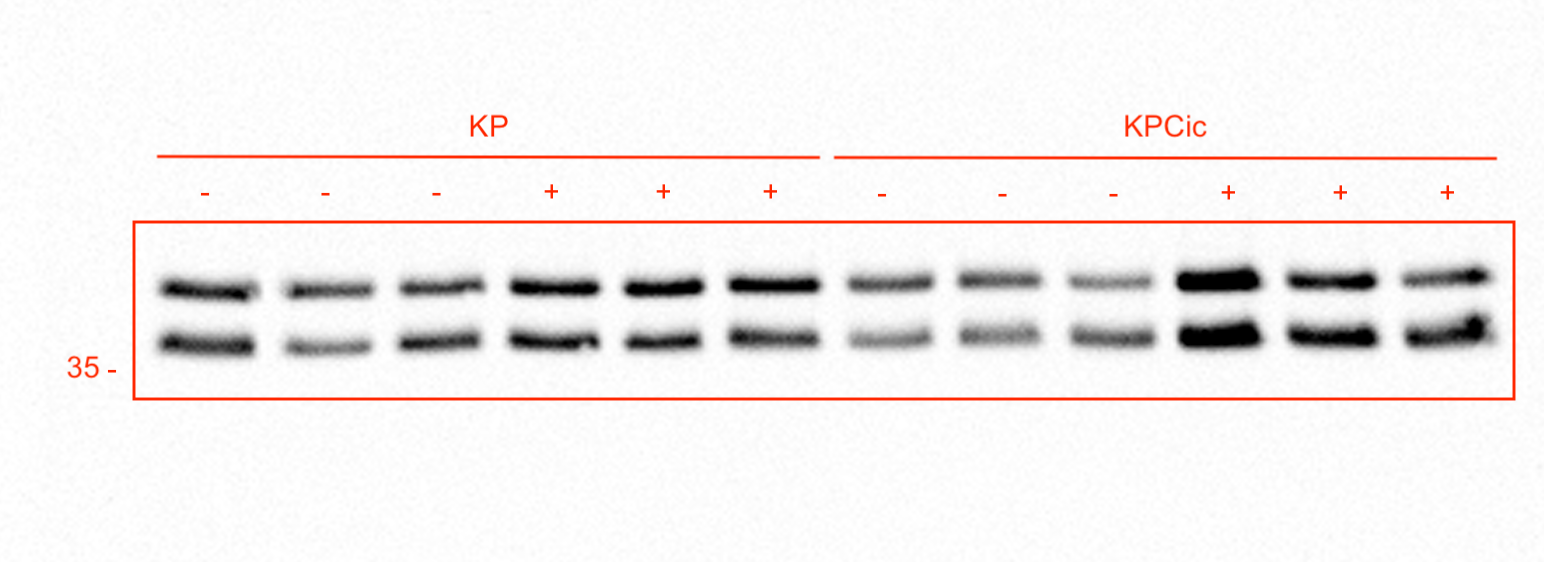

Supplement: Supplementary file 12 — Source data Fig. 6 [file 44321_2025_326_MOESM12_ESM.zip › Fig6/Fig6c/ERK.tif]

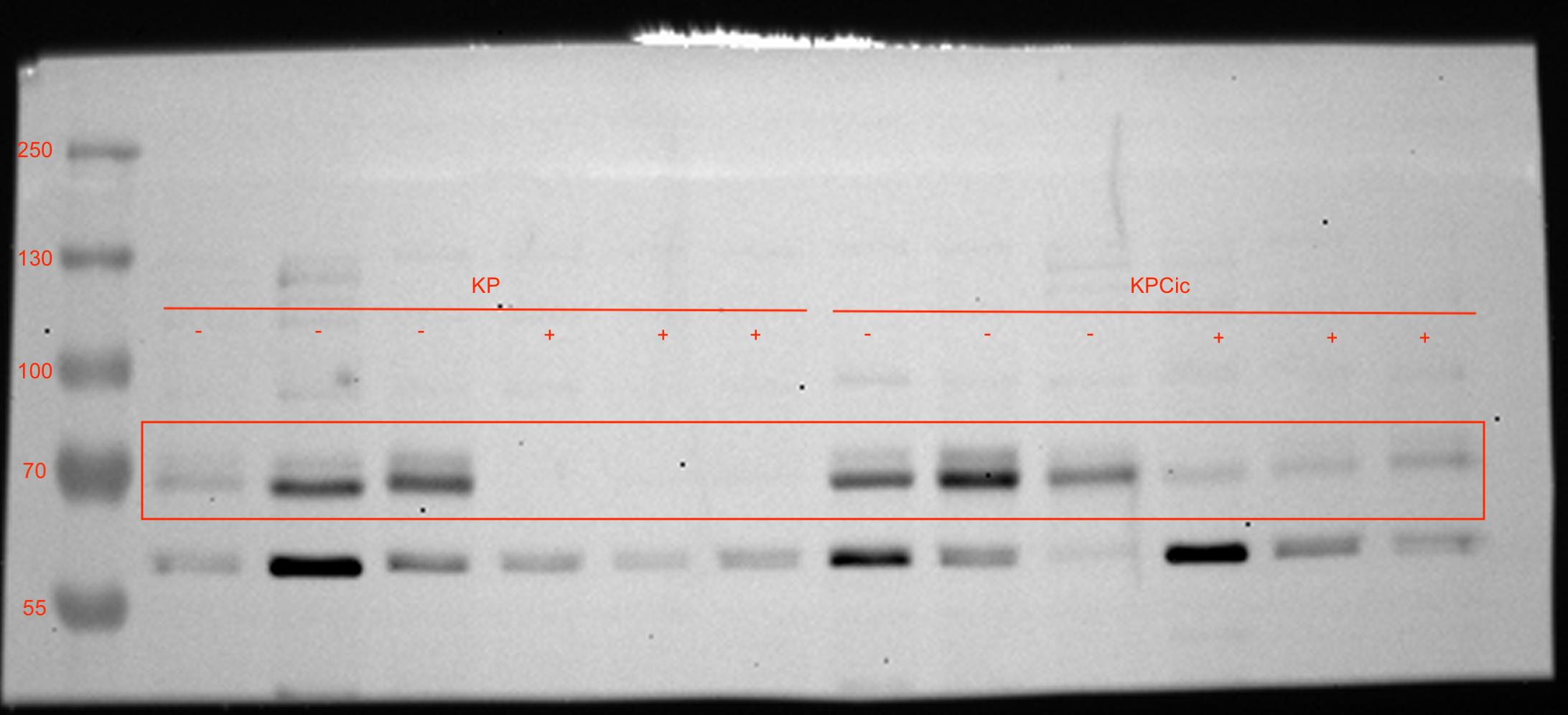

Supplement: Supplementary file 12 — Source data Fig. 6 [file 44321_2025_326_MOESM12_ESM.zip › Fig6/Fig6c/ETV5.tif]

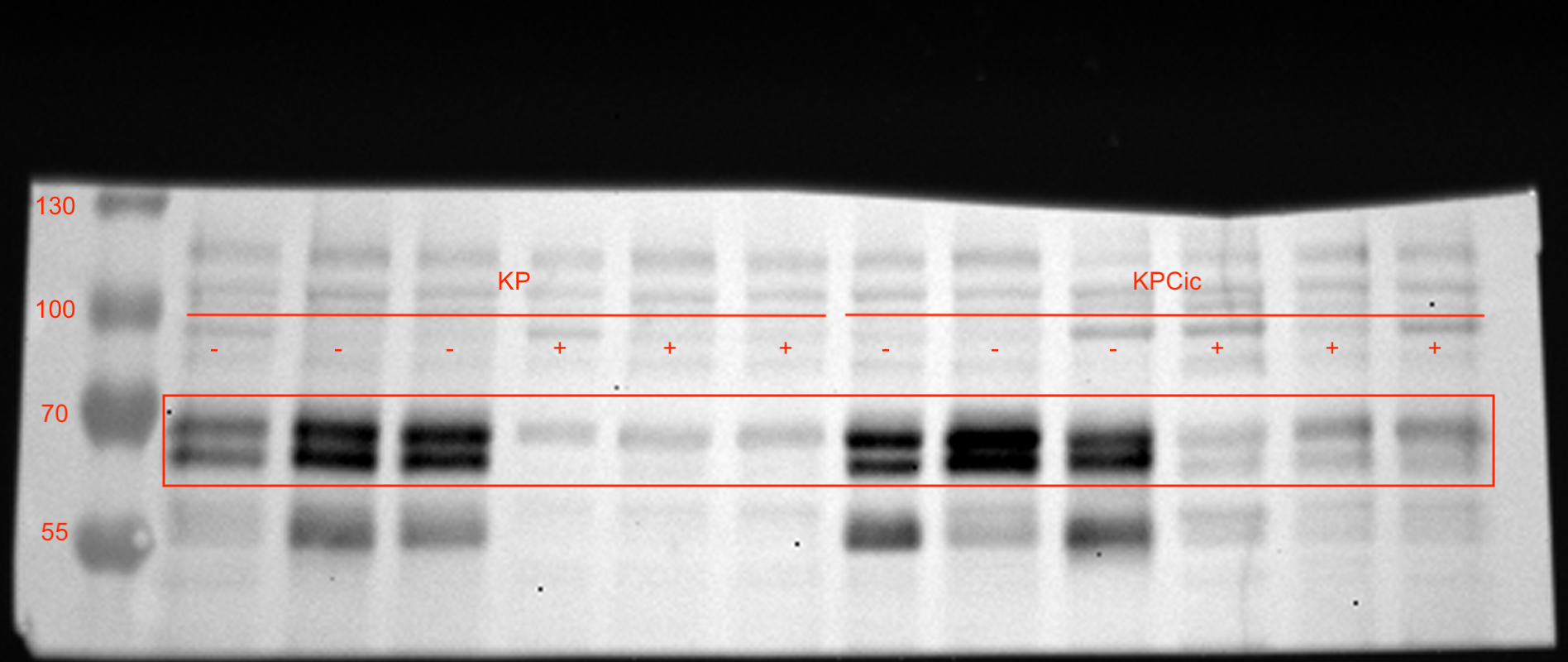

Supplement: Supplementary file 12 — Source data Fig. 6 [file 44321_2025_326_MOESM12_ESM.zip › Fig6/Fig6c/ETV4.tif]

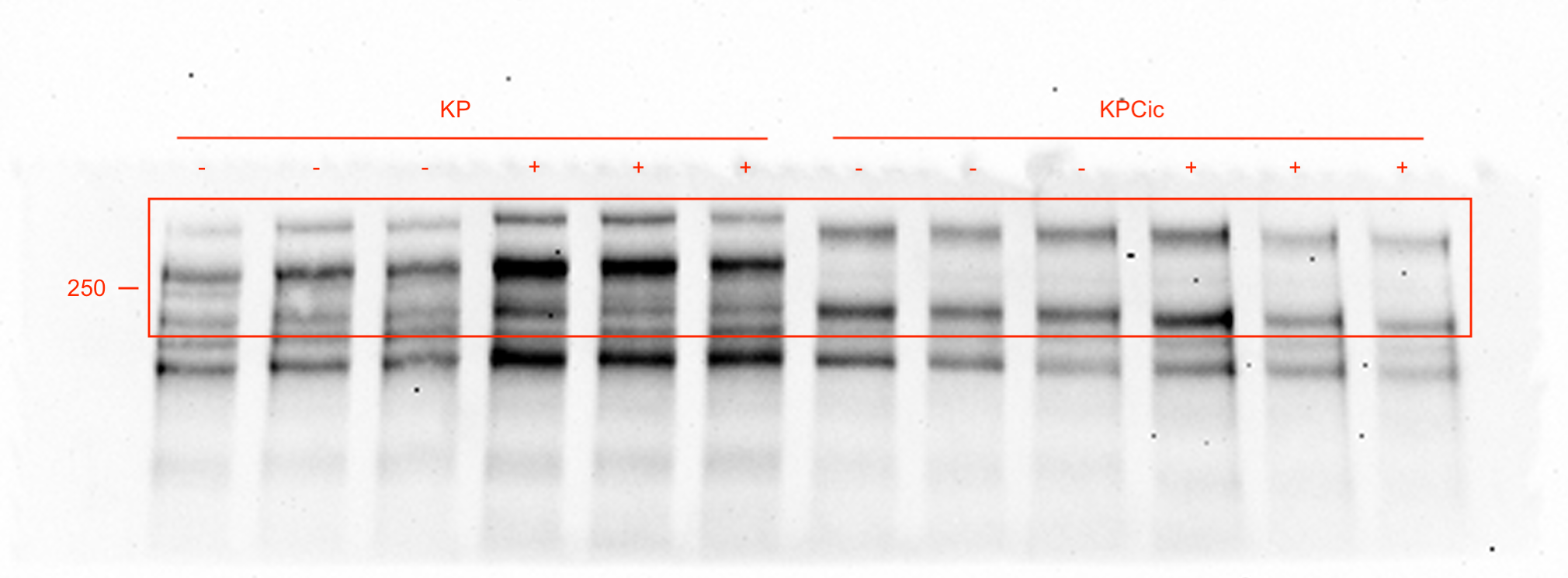

Supplement: Supplementary file 12 — Source data Fig. 6 [file 44321_2025_326_MOESM12_ESM.zip › Fig6/Fig6c/CIC.tif]

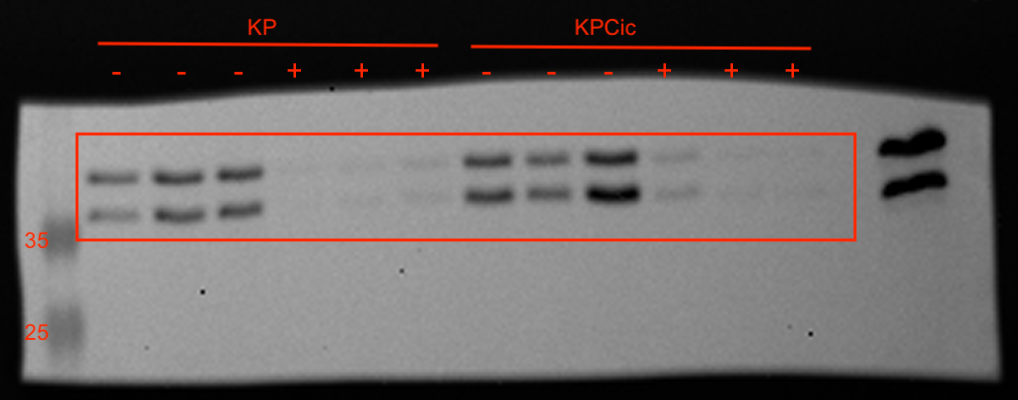

Supplement: Supplementary file 12 — Source data Fig. 6 [file 44321_2025_326_MOESM12_ESM.zip › Fig6/Fig6c/pERK.tif]

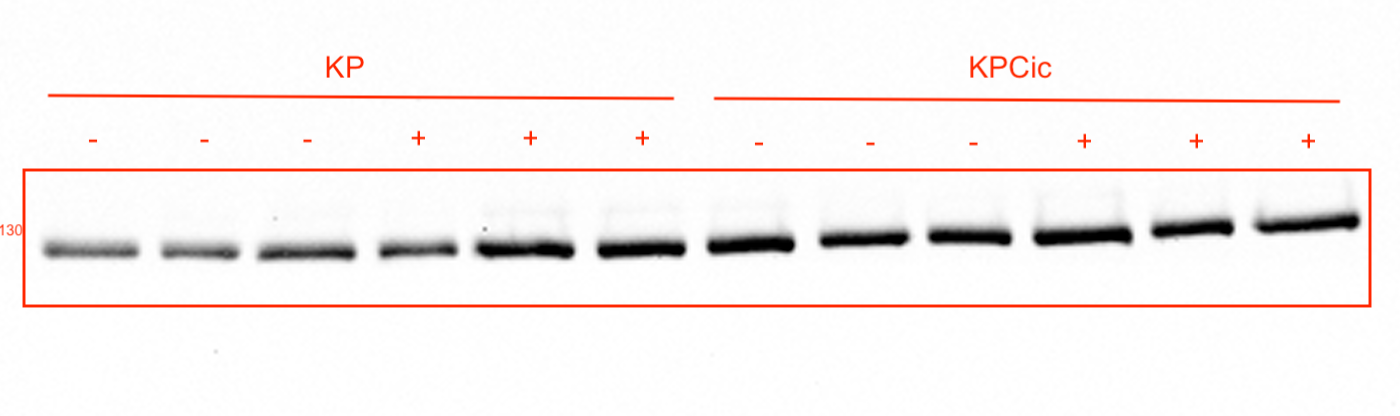

Supplement: Supplementary file 12 — Source data Fig. 6 [file 44321_2025_326_MOESM12_ESM.zip › Fig6/Fig6c/Vinculin.tif]

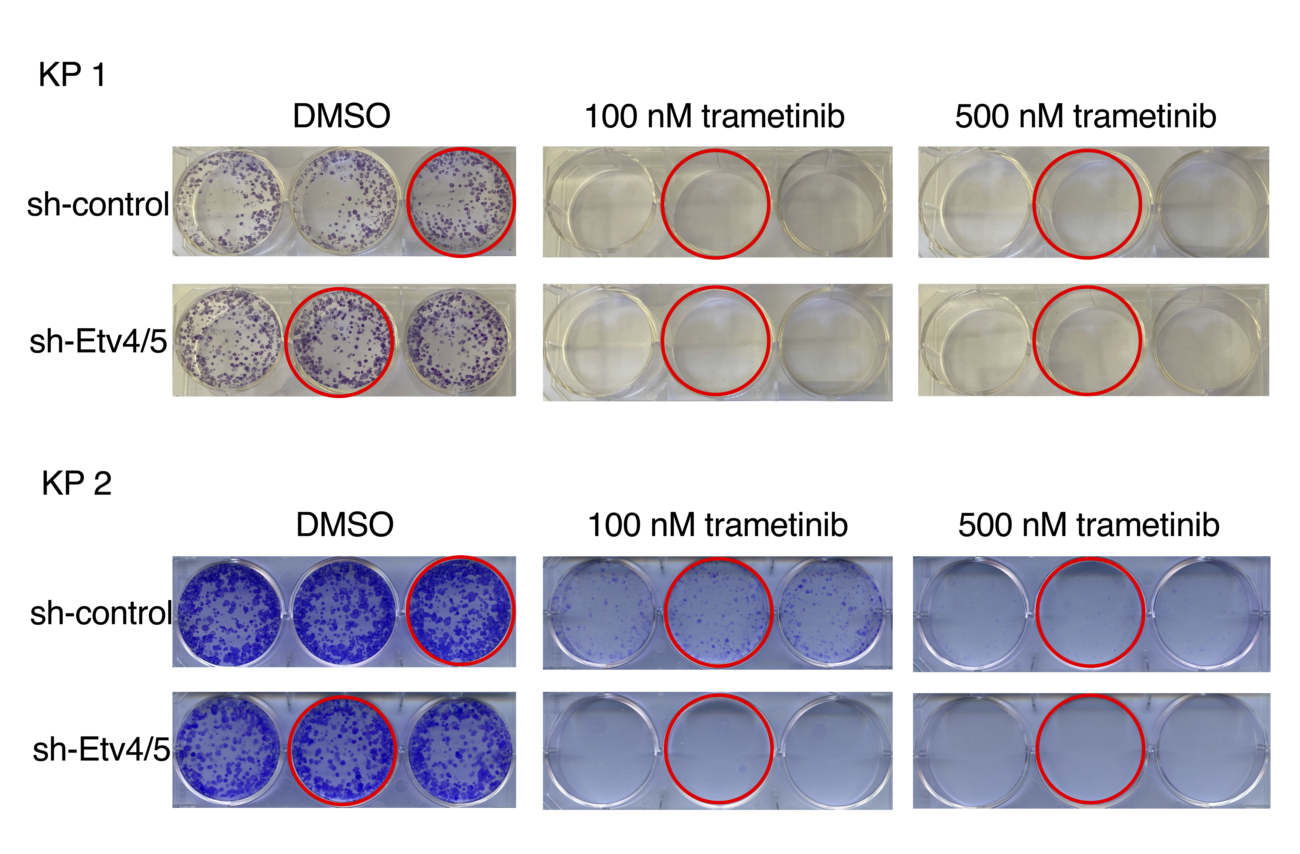

Supplement: Supplementary file 12 — Source data Fig. 6 [file 44321_2025_326_MOESM12_ESM.zip › Fig6/Fig6d/Fig6d-colonies.tiff]

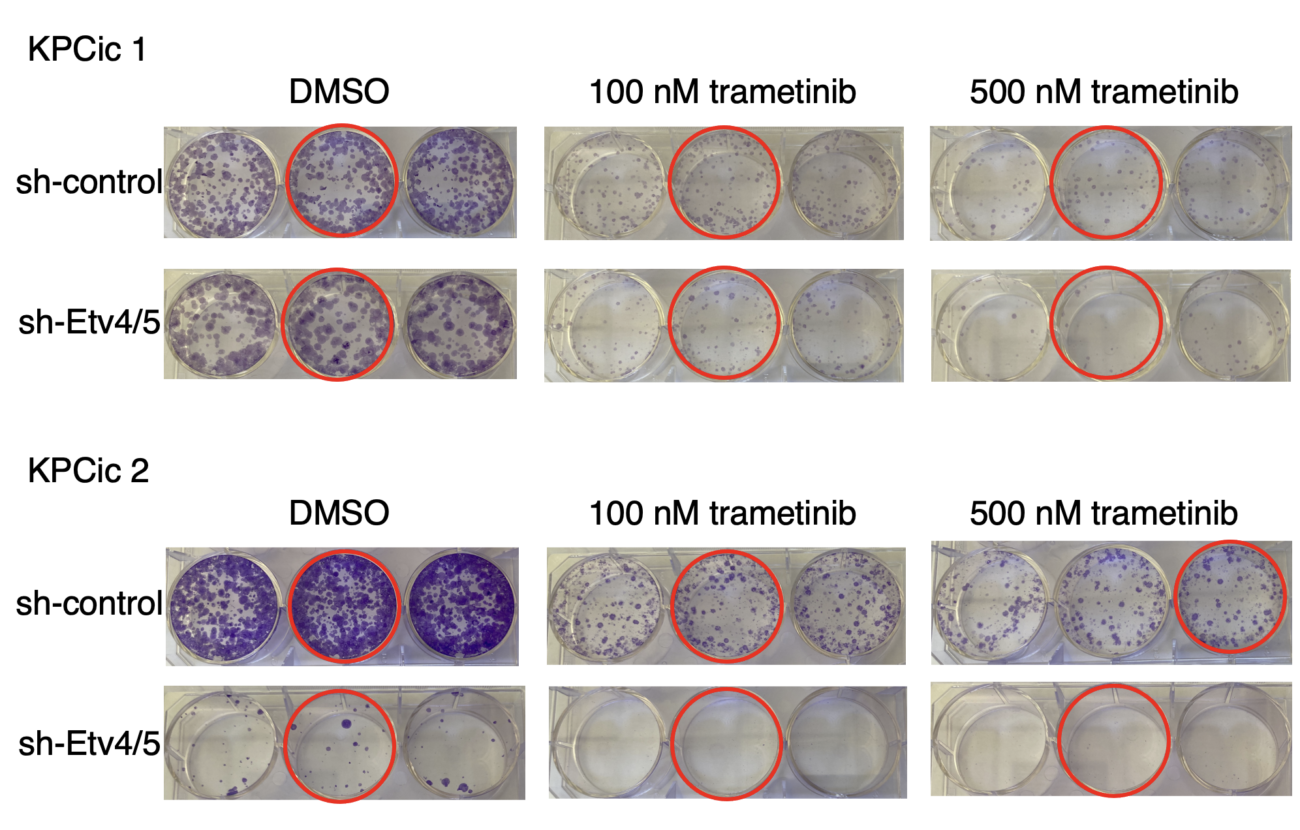

Supplement: Supplementary file 12 — Source data Fig. 6 [file 44321_2025_326_MOESM12_ESM.zip › Fig6/Fig6e/Fig6e-colonies.tif]

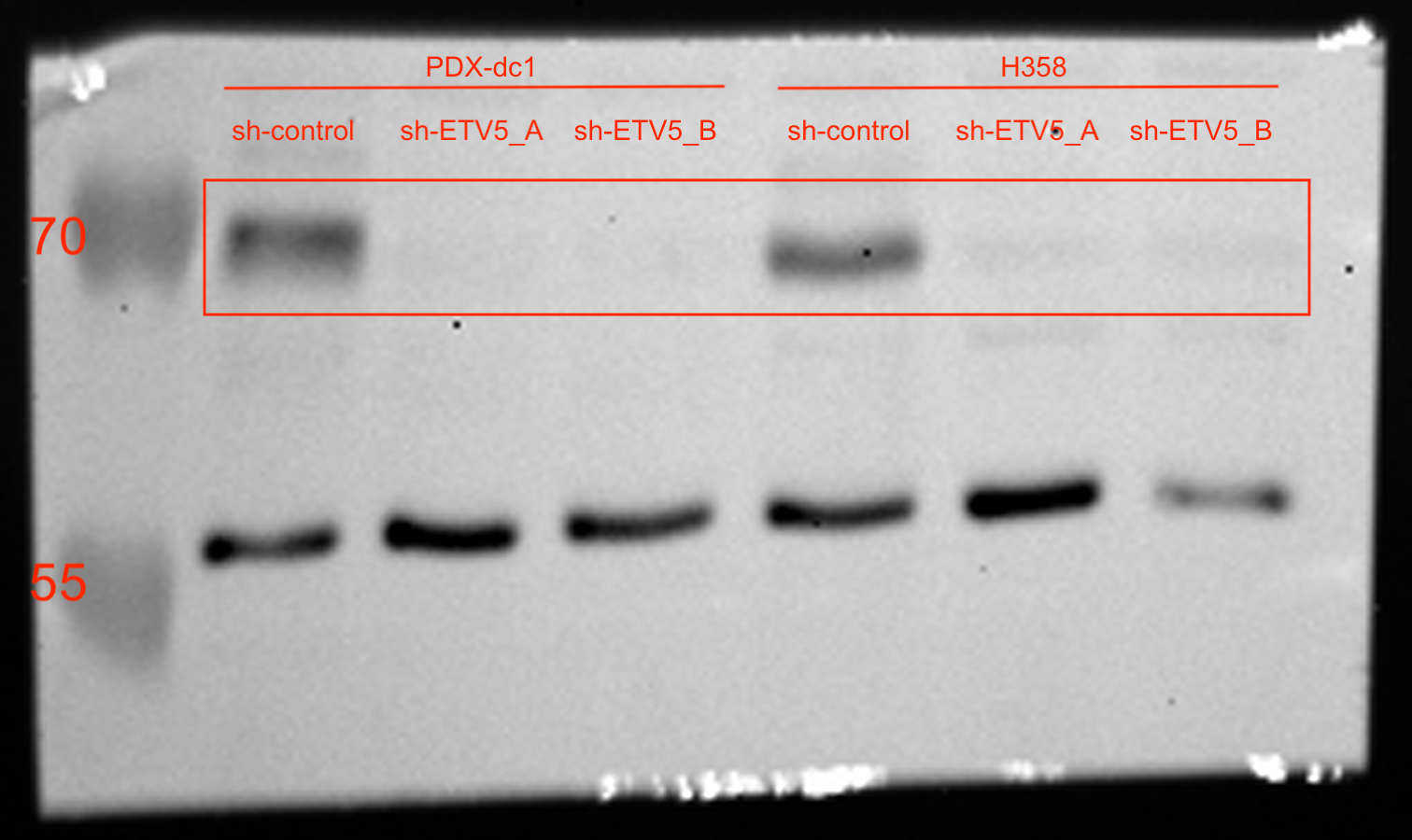

Supplement: Supplementary file 12 — Source data Fig. 6 [file 44321_2025_326_MOESM12_ESM.zip › Fig6/Fig6g/ETV5.tif]

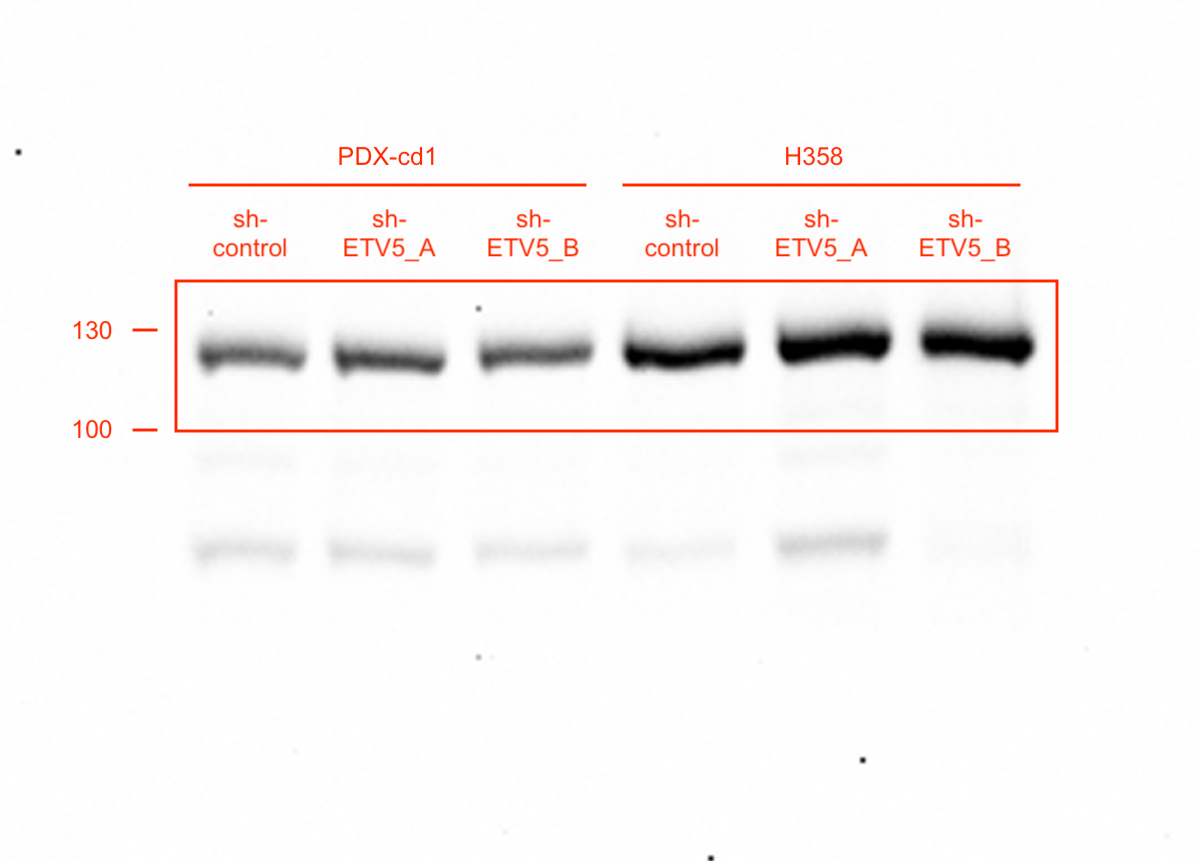

Supplement: Supplementary file 12 — Source data Fig. 6 [file 44321_2025_326_MOESM12_ESM.zip › Fig6/Fig6g/Vinculin ETV5 blot.tiff]

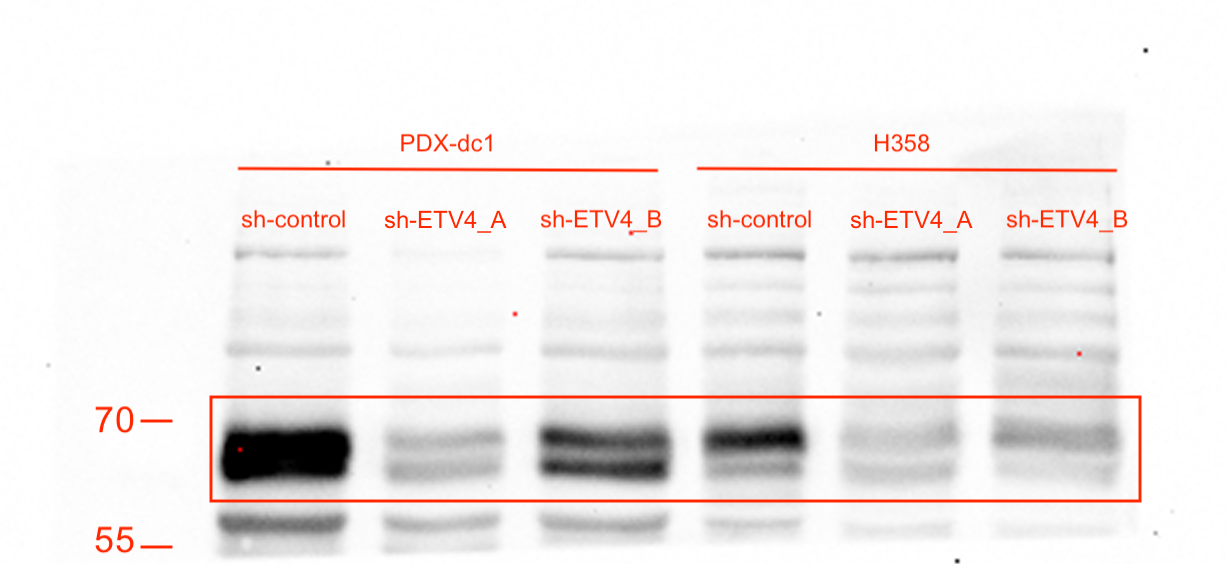

Supplement: Supplementary file 12 — Source data Fig. 6 [file 44321_2025_326_MOESM12_ESM.zip › Fig6/Fig6g/ETV4.tif]

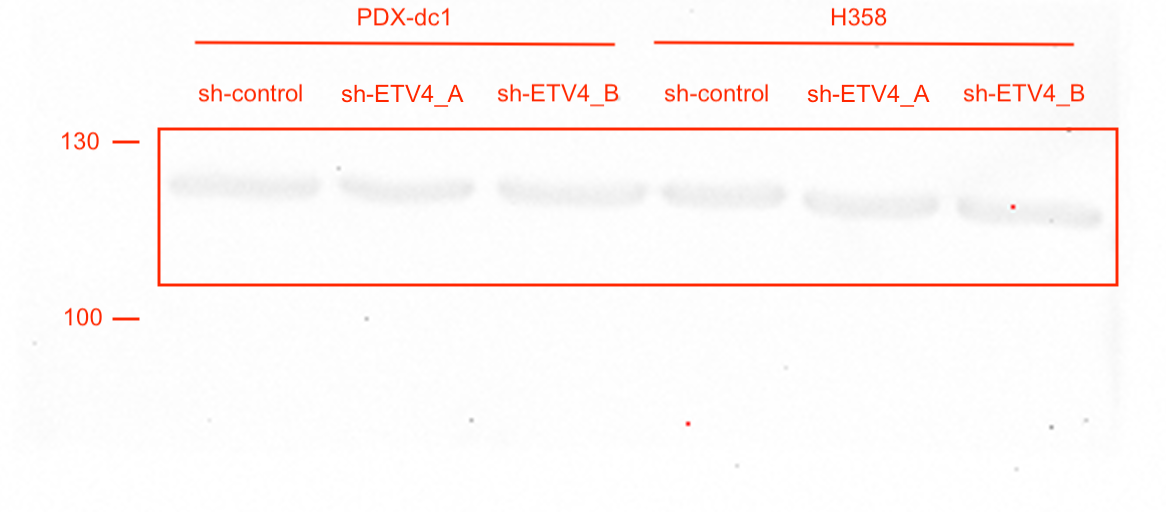

Supplement: Supplementary file 12 — Source data Fig. 6 [file 44321_2025_326_MOESM12_ESM.zip › Fig6/Fig6g/Vinculin ETV4 blot.tif]

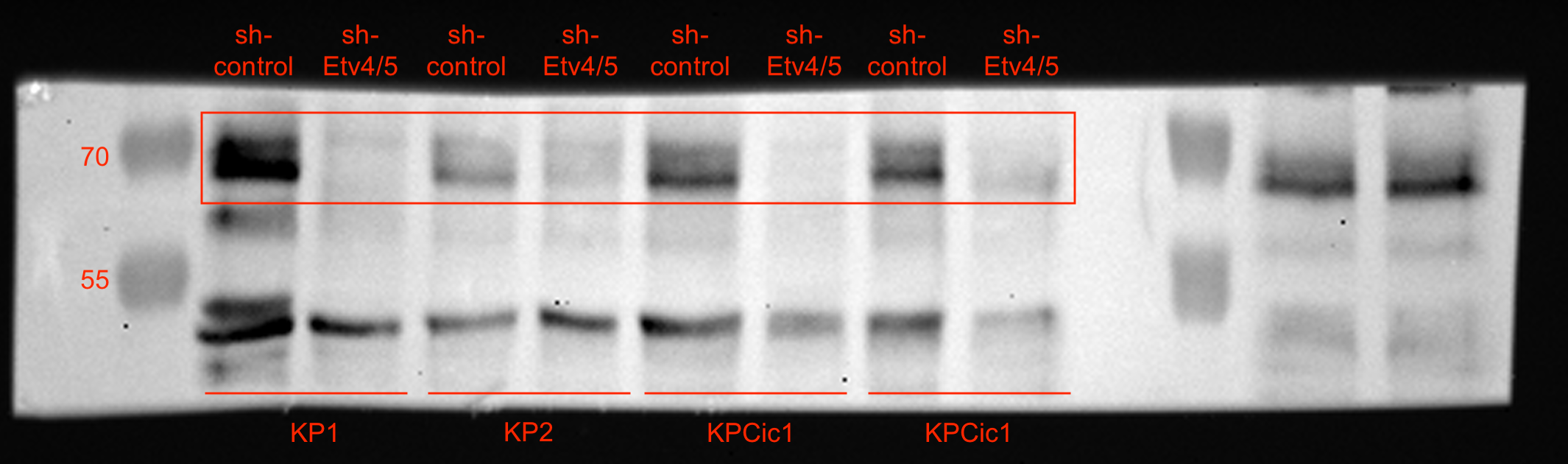

Supplement: Supplementary file 12 — Source data Fig. 6 [file 44321_2025_326_MOESM12_ESM.zip › Fig6/Fig6f/ETV5.tif]

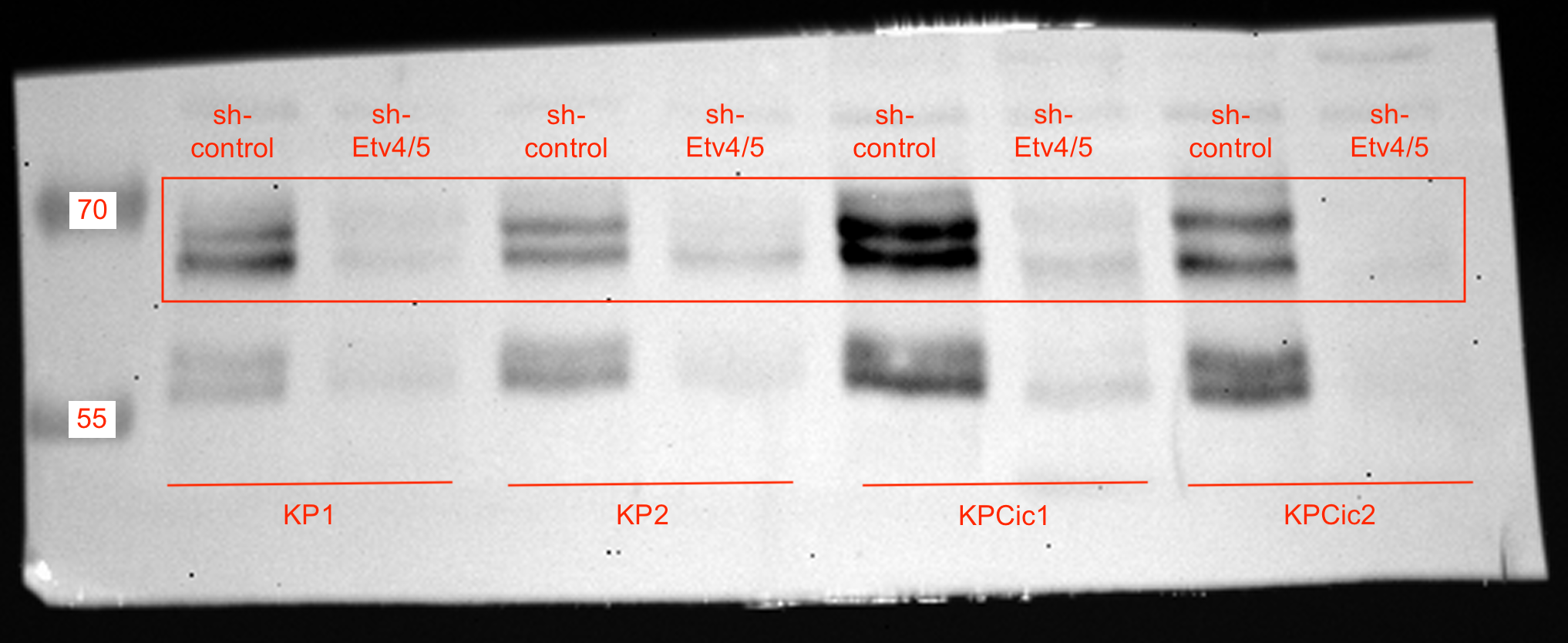

Supplement: Supplementary file 12 — Source data Fig. 6 [file 44321_2025_326_MOESM12_ESM.zip › Fig6/Fig6f/ETV4.tif]

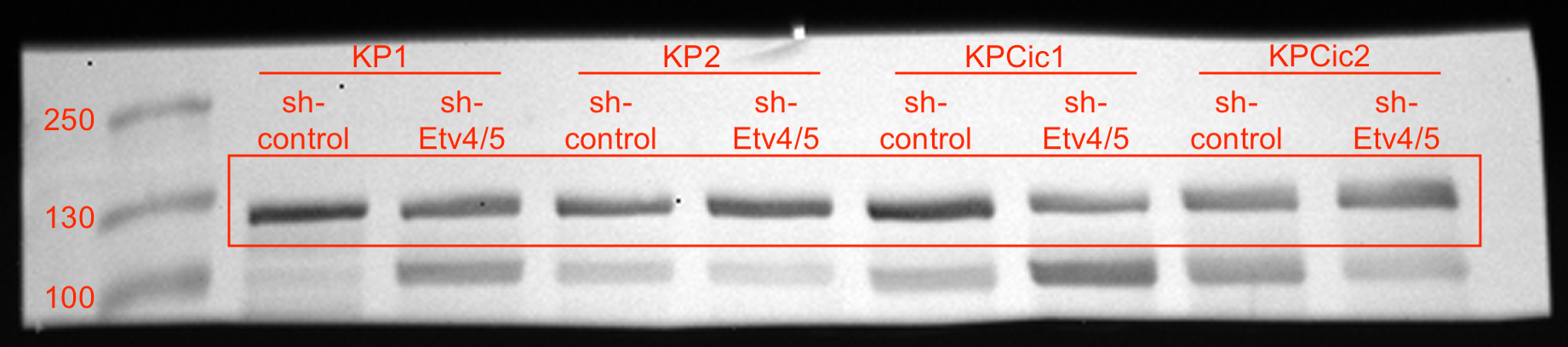

Supplement: Supplementary file 12 — Source data Fig. 6 [file 44321_2025_326_MOESM12_ESM.zip › Fig6/Fig6f/Vinculin.tif]

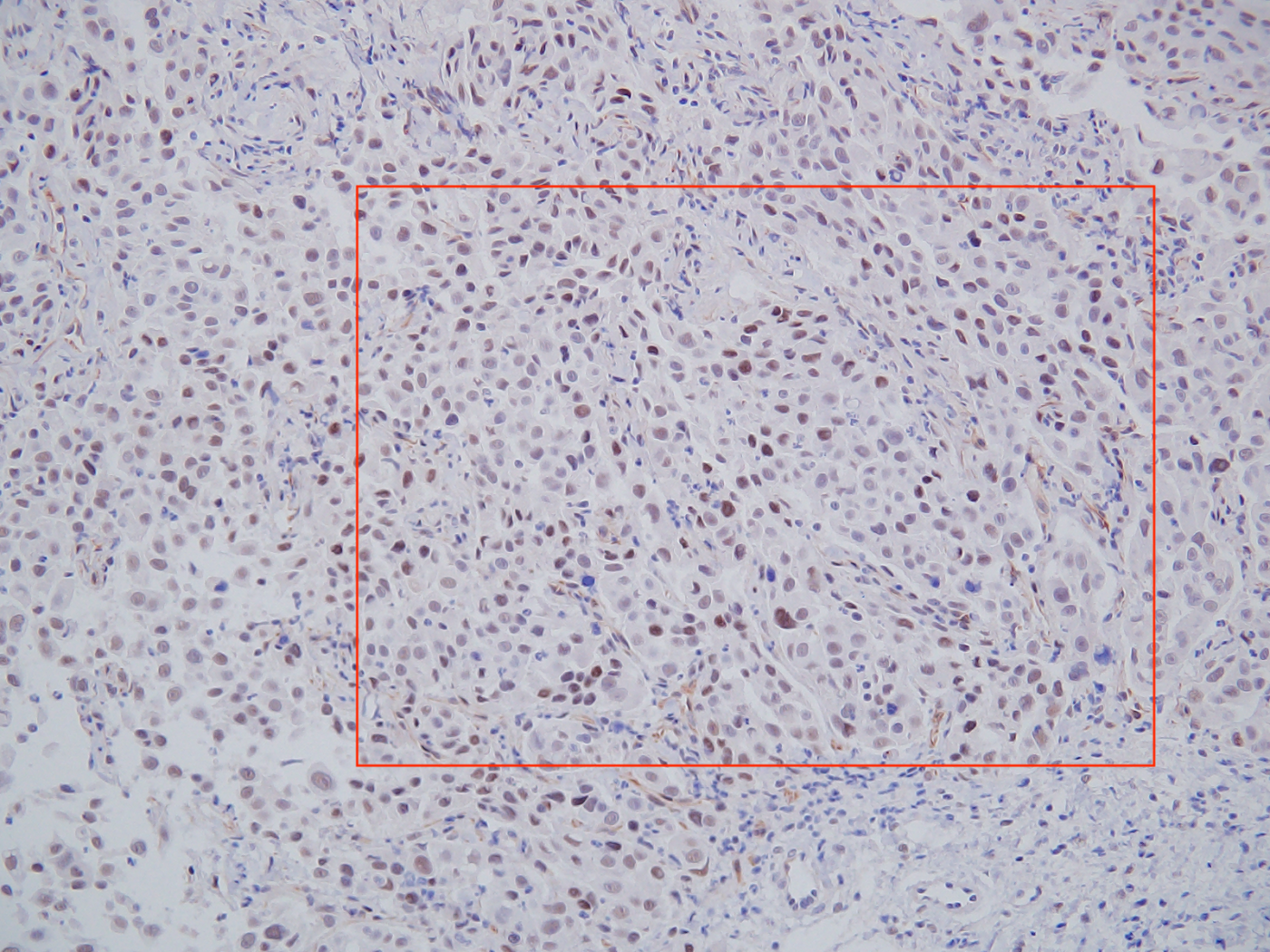

Supplement: Supplementary file 13 — Source data Fig. 7 [file 44321_2025_326_MOESM13_ESM.zip › Fig7/Fig7f/TP40 ETV5.tif]

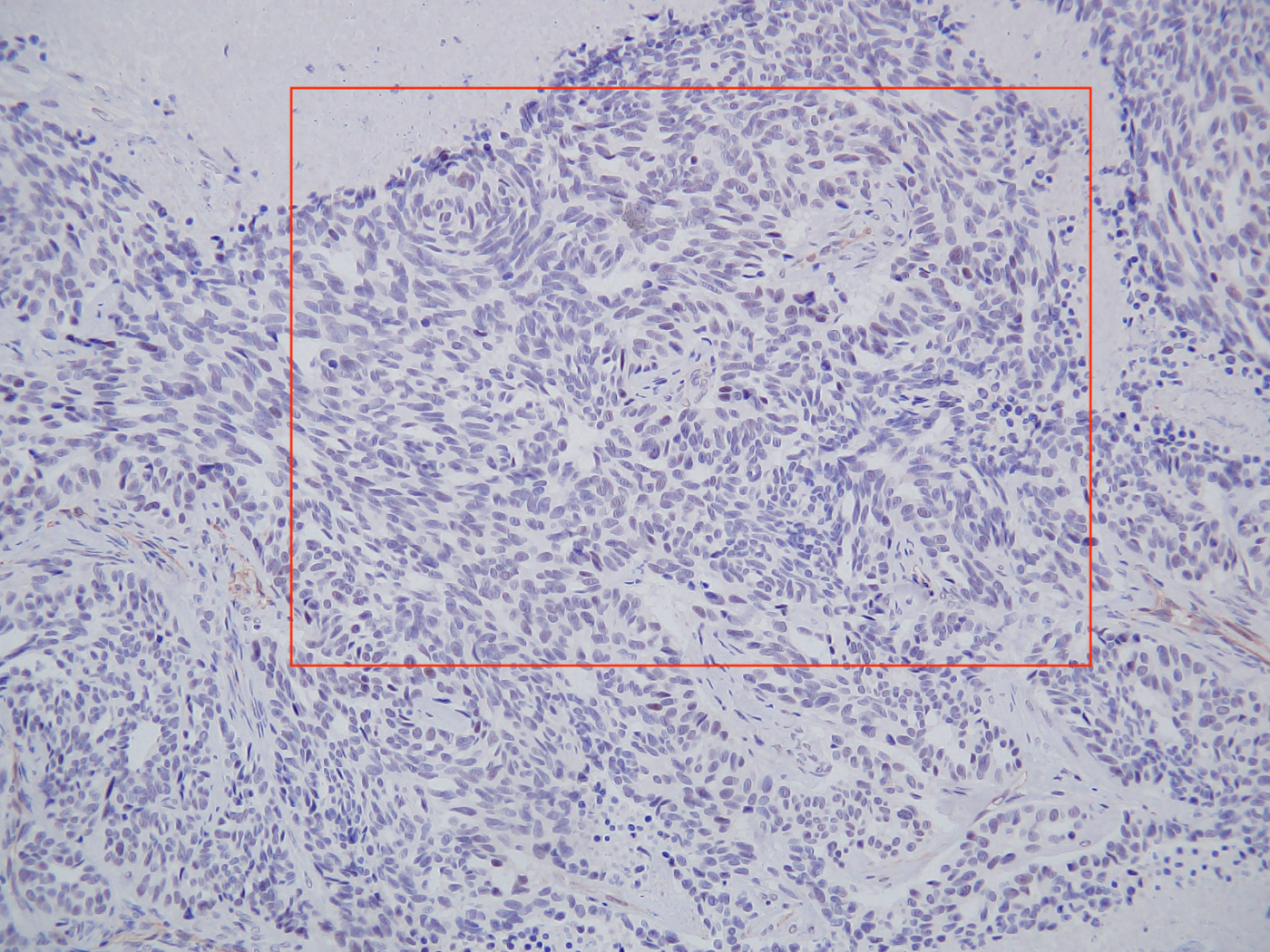

Supplement: Supplementary file 13 — Source data Fig. 7 [file 44321_2025_326_MOESM13_ESM.zip › Fig7/Fig7f/TP91 ETV5.tif]

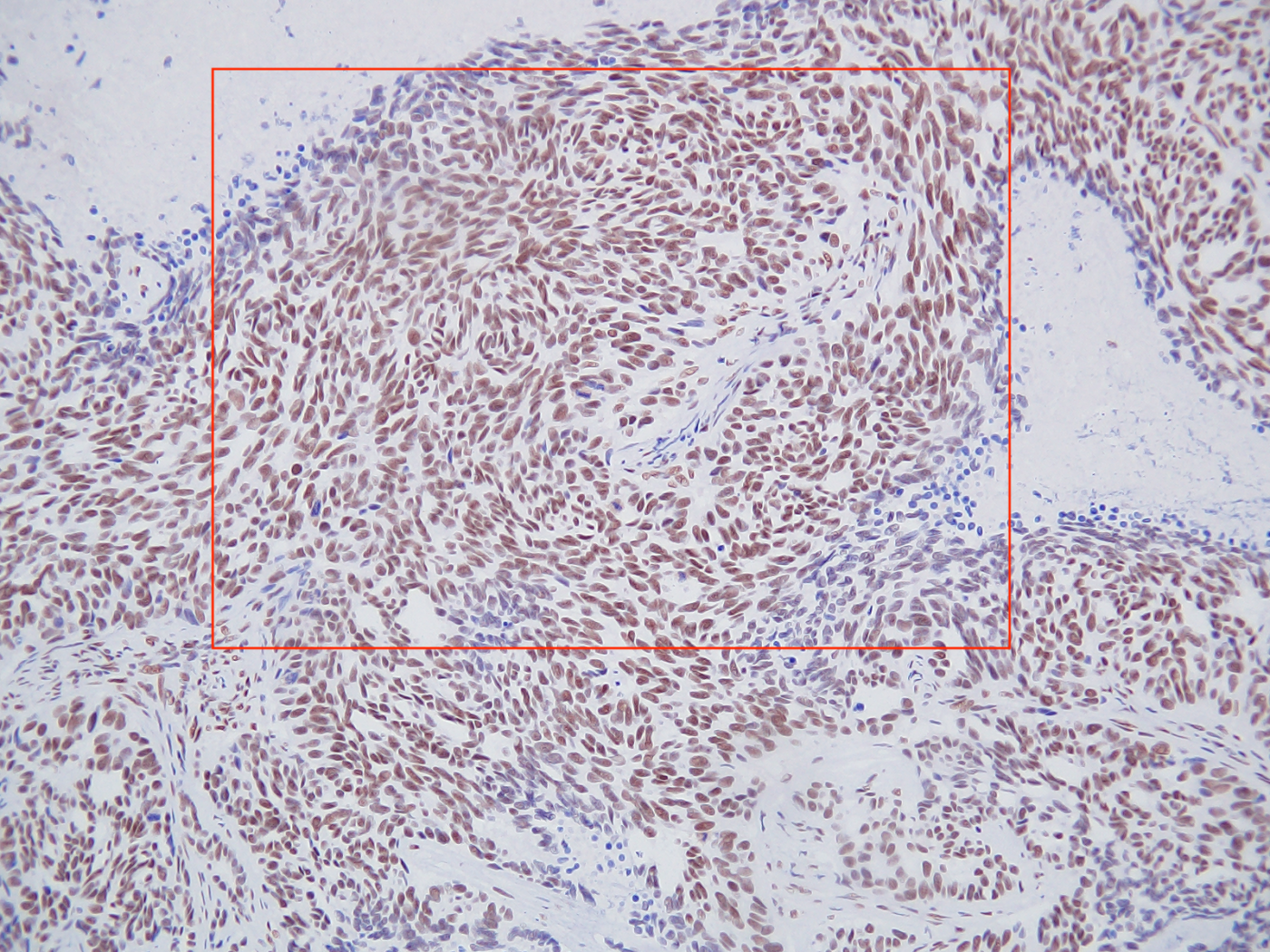

Supplement: Supplementary file 13 — Source data Fig. 7 [file 44321_2025_326_MOESM13_ESM.zip › Fig7/Fig7f/TP91 CIC.tif]

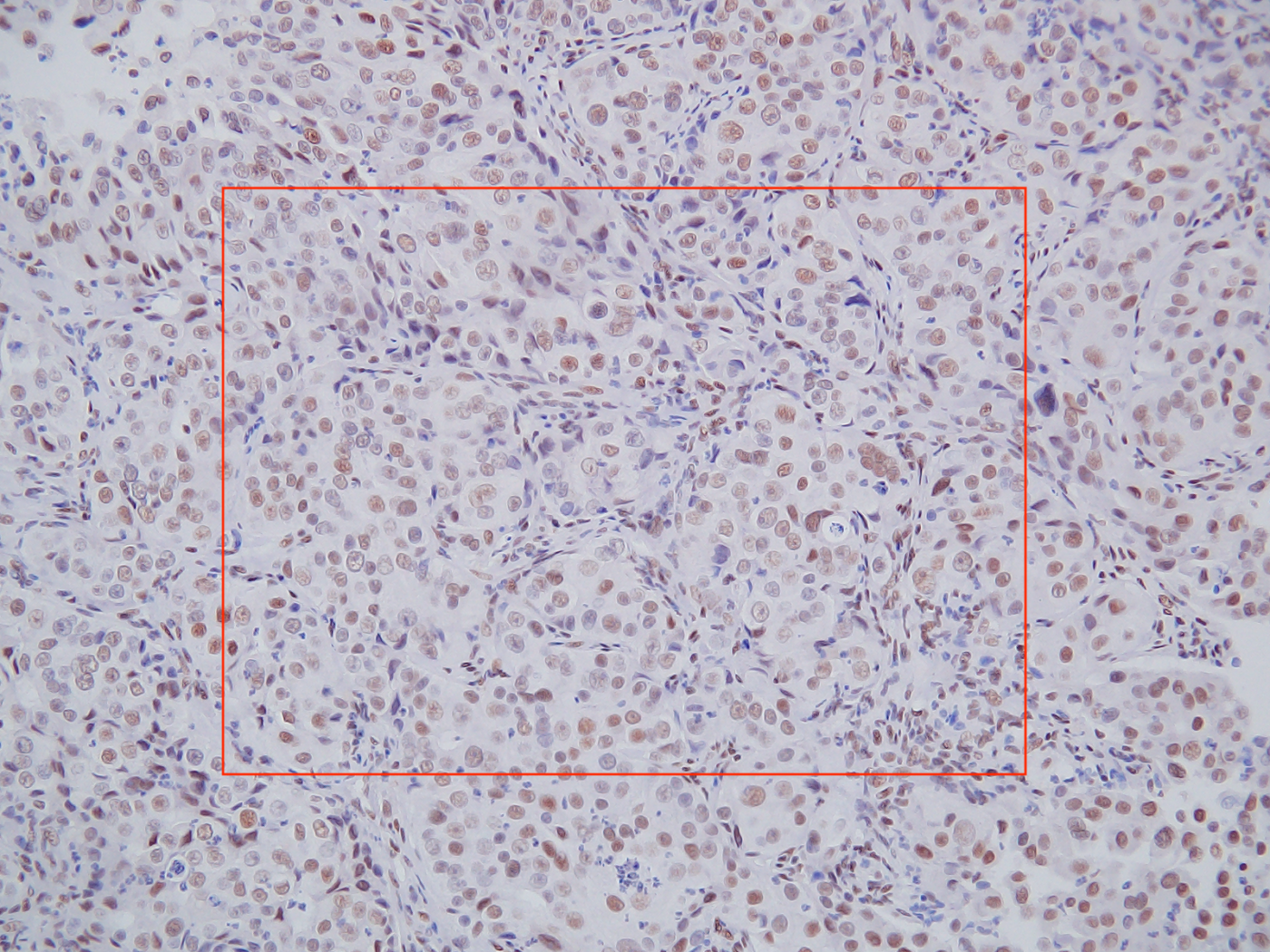

Supplement: Supplementary file 13 — Source data Fig. 7 [file 44321_2025_326_MOESM13_ESM.zip › Fig7/Fig7f/TP40 CIC.tif]

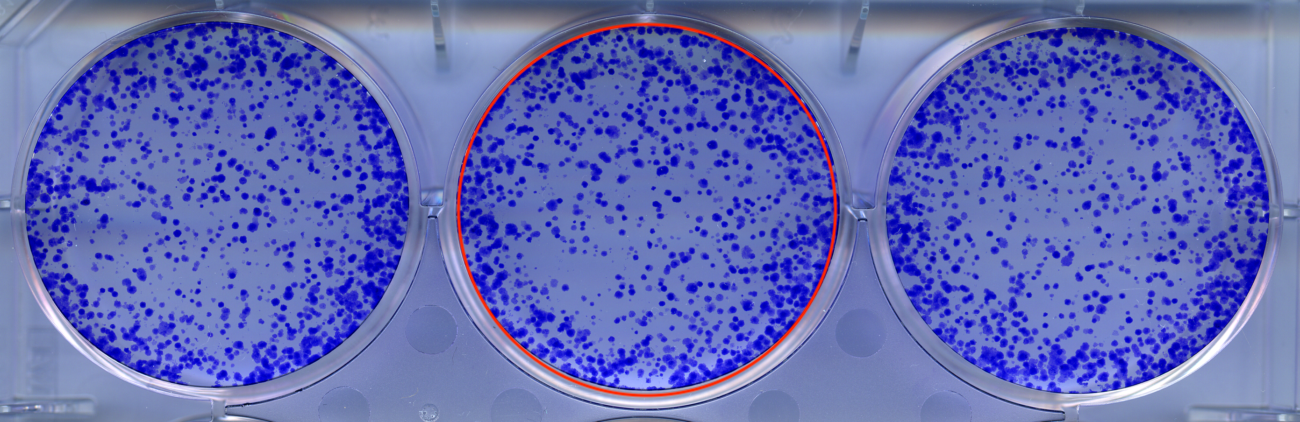

Supplement: Supplementary file 13 — Source data Fig. 7 [file 44321_2025_326_MOESM13_ESM.zip › Fig7/Fig7e/sg-control-PFK15.tif]

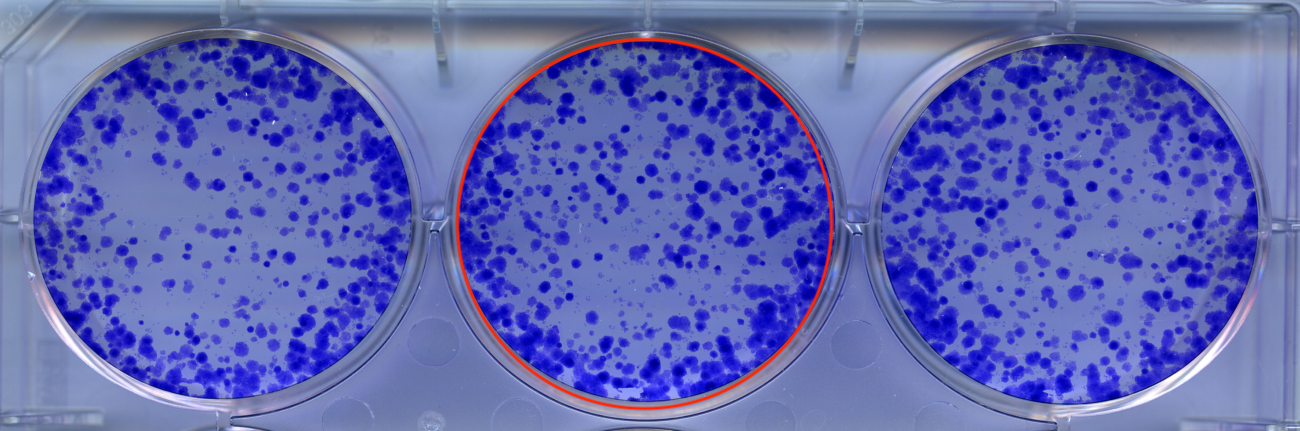

Supplement: Supplementary file 13 — Source data Fig. 7 [file 44321_2025_326_MOESM13_ESM.zip › Fig7/Fig7e/sg-CIC-PFK15.tif]

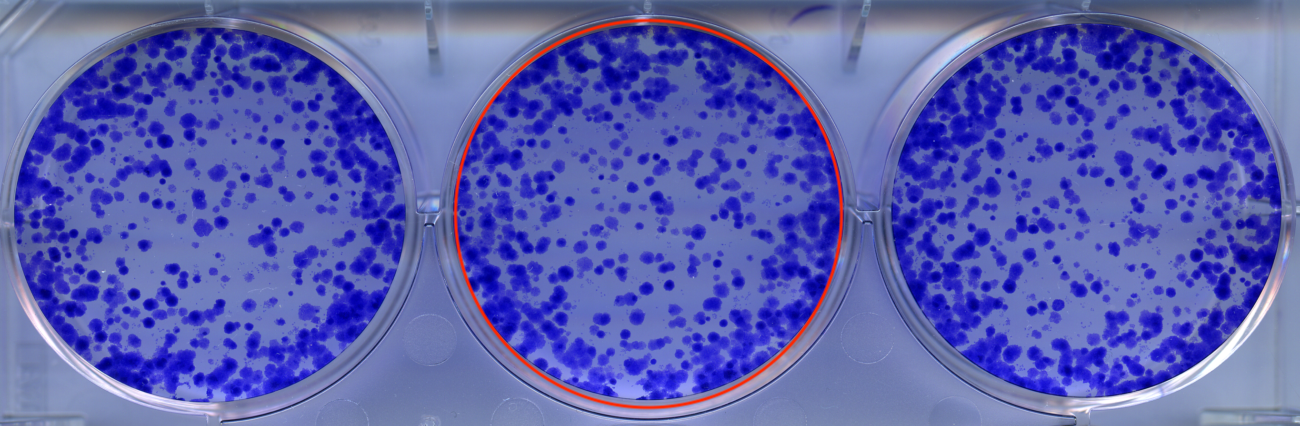

Supplement: Supplementary file 13 — Source data Fig. 7 [file 44321_2025_326_MOESM13_ESM.zip › Fig7/Fig7e/sg-CIC DMSO.tif]

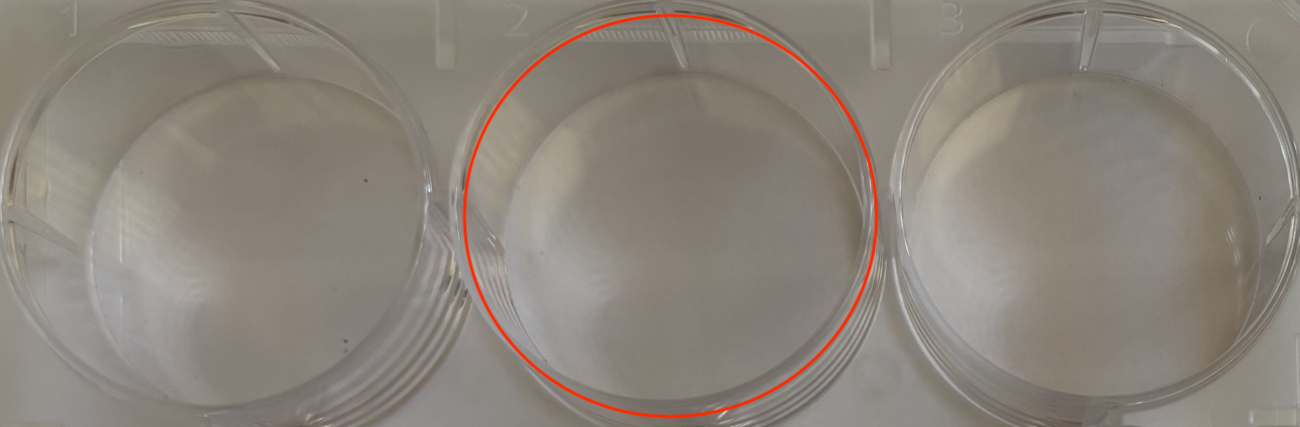

Supplement: Supplementary file 13 — Source data Fig. 7 [file 44321_2025_326_MOESM13_ESM.zip › Fig7/Fig7e/sg-control-trametinib+PFK.tif]

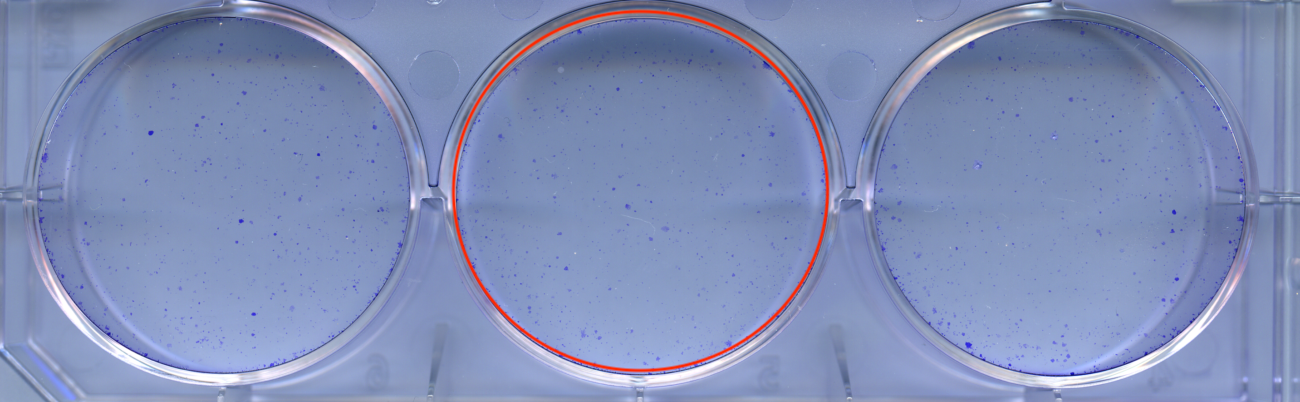

Supplement: Supplementary file 13 — Source data Fig. 7 [file 44321_2025_326_MOESM13_ESM.zip › Fig7/Fig7e/sg-control-trametinib.tif]

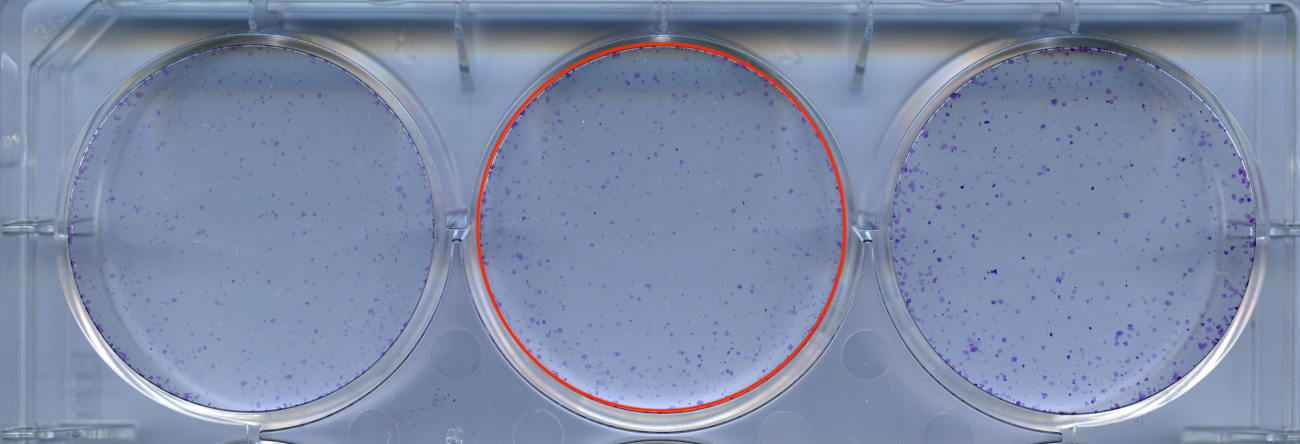

Supplement: Supplementary file 13 — Source data Fig. 7 [file 44321_2025_326_MOESM13_ESM.zip › Fig7/Fig7e/sg-CIC-trametinib+PFK15.tif]

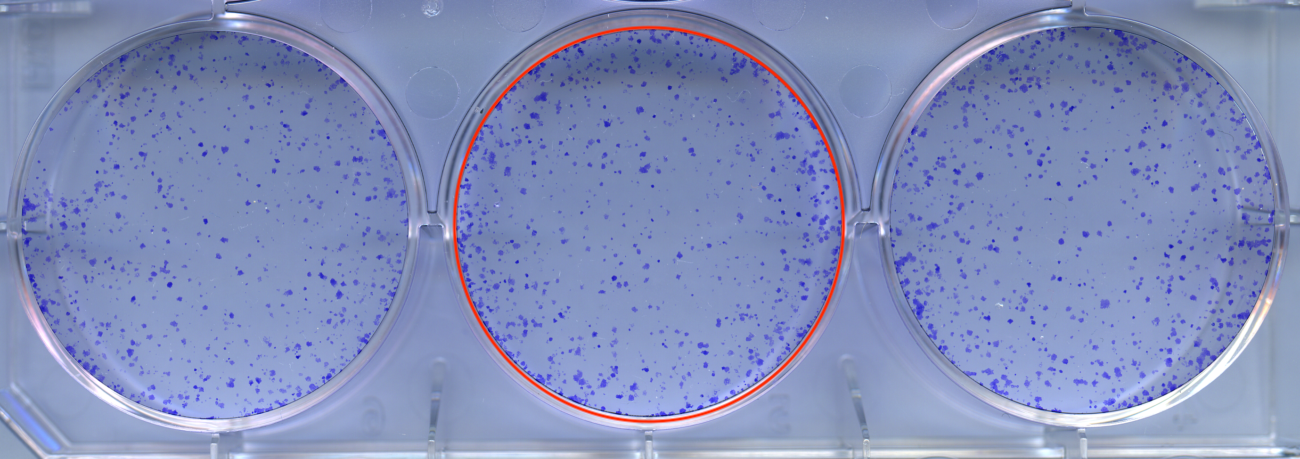

Supplement: Supplementary file 13 — Source data Fig. 7 [file 44321_2025_326_MOESM13_ESM.zip › Fig7/Fig7e/sg-CIC-trametinib.tif]

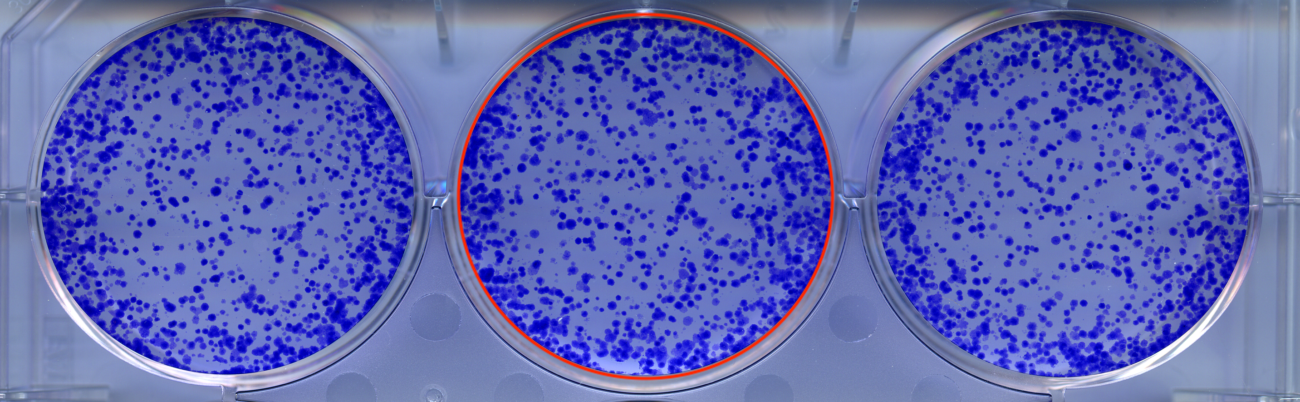

Supplement: Supplementary file 13 — Source data Fig. 7 [file 44321_2025_326_MOESM13_ESM.zip › Fig7/Fig7e/sg-control DMSO.tif]

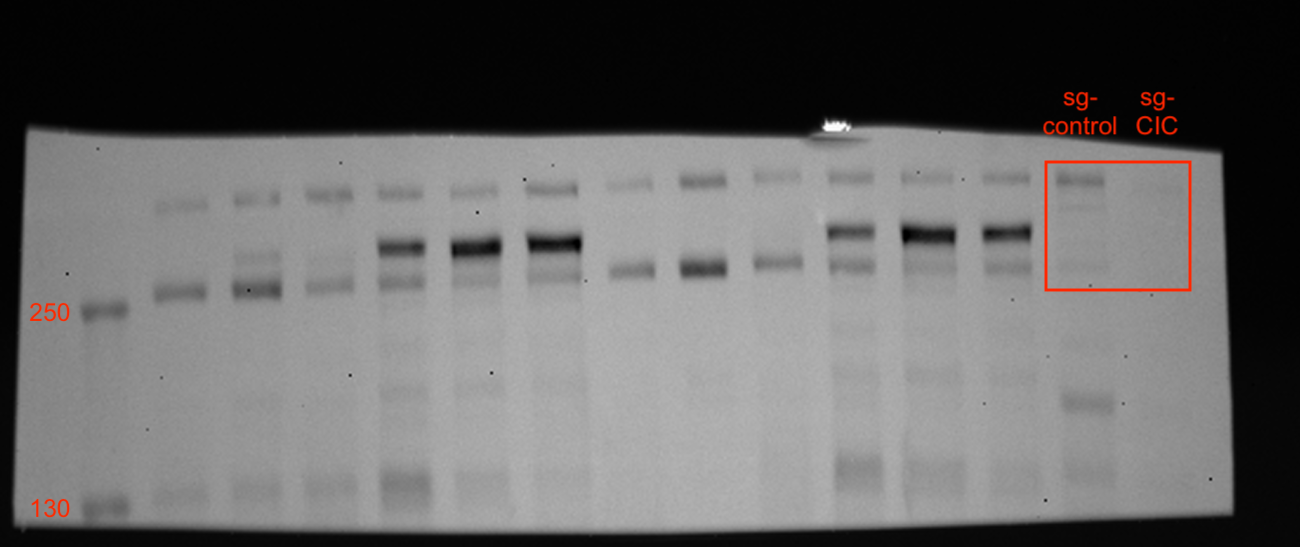

Supplement: Supplementary file 13 — Source data Fig. 7 [file 44321_2025_326_MOESM13_ESM.zip › Fig7/Fig7d/CIC.tif]

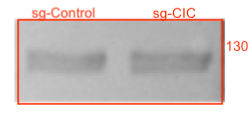

Supplement: Supplementary file 13 — Source data Fig. 7 [file 44321_2025_326_MOESM13_ESM.zip › Fig7/Fig7d/Vinculin.tiff]
