# Supplementary material for: The repressor Capicua is a barrier to lung tumor development driven by Kras/Trp53 mutations
Source: EMBO Mol Med. 2025 Nov 11;17(12):3377–406. doi: 10.1038/s44321-025-00326-z (PMC12686060; doi:10.1038/s44321-025-00326-z)
Supplement: Supplementary file 14 — Expanded View Figures [file 44321_2025_326_MOESM14_ESM.pdf]

# Expanded View Figures

**Figure EV1. Tumor burden in KP mice expressing WT CIC or CIC$^{\Delta 2\text{-}6}$.**

(**A**) Quantification of lesions in KP (blue bar, $n = 8$) or KPCic mice (red bar, $n = 8$) infected with $5 \times 10^5$ pfu Ad-Cre at a humane endpoint. Results are shown as mean ± SD. Statistics, unpaired *t*-test. (**B**) Quantification of tumor grades (G1–G5) in lesions from KP (blue bars, $n = 8$) or KPCic mice (red bars, $n = 8$) infected with $5 \times 10^5$ pfu Ad-Cre at a humane endpoint. Results are shown as mean ± SD. Statistics, multiple *t*-tests. (**C**) Quantification of HMGA2+ tumors in KP (blue bar, $n = 3$) or KPCic mice (red bar, $n = 4$) infected with $5 \times 10^5$ pfu Ad-Cre at a humane endpoint. Results are shown as mean ± SD. Statistics, unpaired *t*-test. (**D**) Representative images of HMGA2 immunostaining in lung sections from a KP and a KPCic mouse infected with $5 \times 10^5$ pfu Ad-Cre at a humane endpoint. Scale bar, 2 mm. (**E**) SPC, TTF-1, and HMGA2 immunostaining in consecutive sections from a lung tumor obtained from a KP mouse. Scale bar, 200 µm. (**F**) Quantification of tumor grades (G1–G5) in lesions from KP (blue bars, $n = 10$) or KPCic mice (red bars, $n = 11$) infected with $5 \times 10^7$ pfu Ad-Cre 5 months after infection. Results are shown as mean ± SD. Statistics, multiple *t*-tests. (**G**) Quantification of HMGA2+ tumors in KP (blue bar, $n = 3$) or KPCic mice (red bar, $n = 3$) infected with $5 \times 10^7$ pfu Ad-Cre 5 months after infection. Results are shown as mean ± SD. (**H**) Representative images of HMGA2 immunostaining in lung sections from a KP and a KPCic mouse infected with $5 \times 10^7$ pfu Ad-Cre 5 months after infection. Arrowheads show positive staining. Scale bar, 2 mm.


© The Author(s)

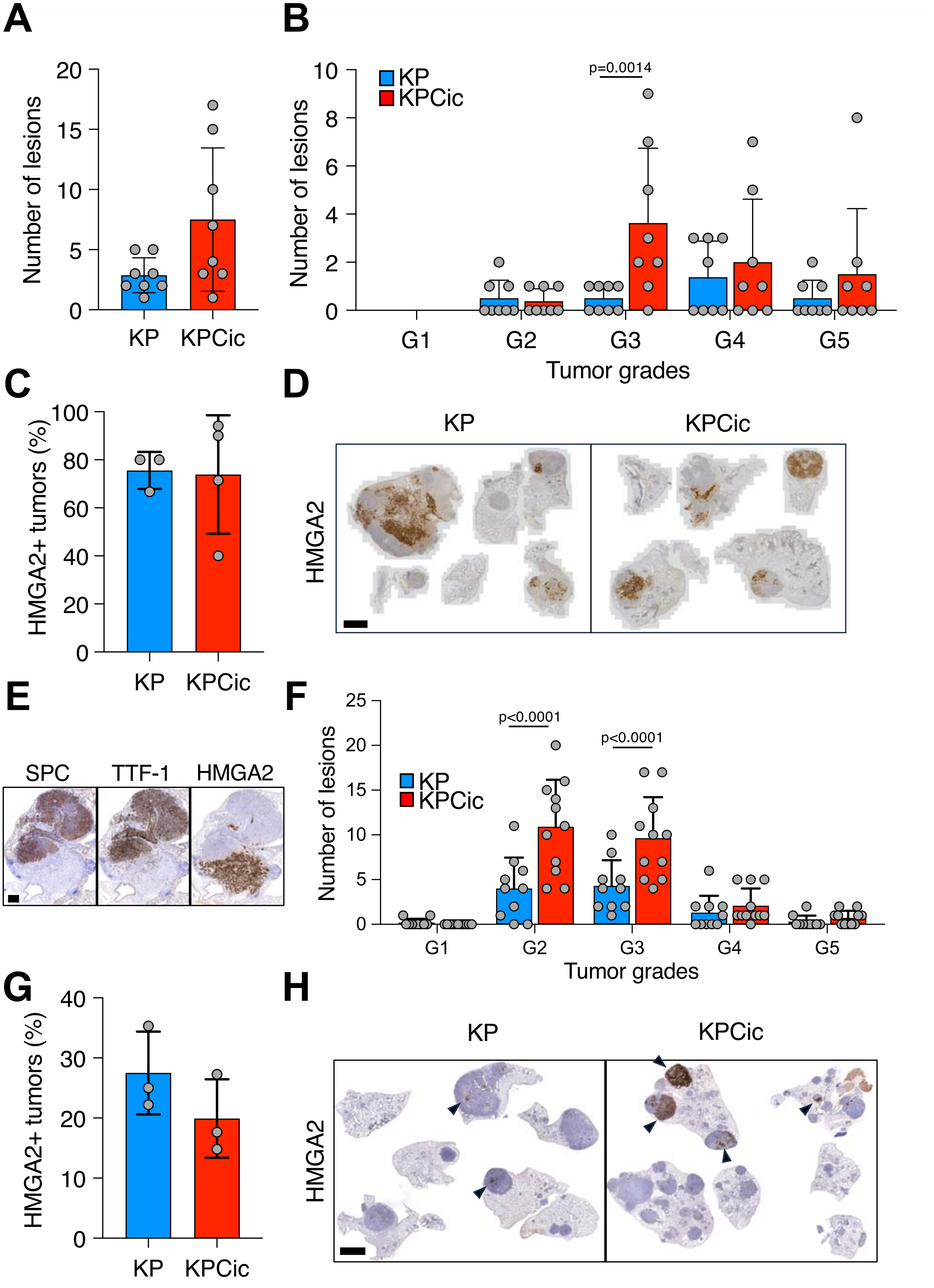
A
Number of lesions
20
15
10
5
0
KP
KPCic
B
Number of lesions
10
8
6
4
2
0
KP
KPCic
p=0.0014
G1
G2
G3
G4
G5
Tumor grades
C
HMGA2+ tumors (%)
100
80
60
40
20
0
KP
KPCic
D
KP
KPCic
HMGA2
E
SPC
TTF-1
HMGA2
F
Number of lesions
25
20
15
10
5
0
p<0.0001
p<0.0001
KP
KPCic
G1
G2
G3
G4
G5
Tumor grades
G
HMGA2+ tumors (%)
40
30
20
10
0
KP
KPCic
H
KP
KPCic
HMGA2

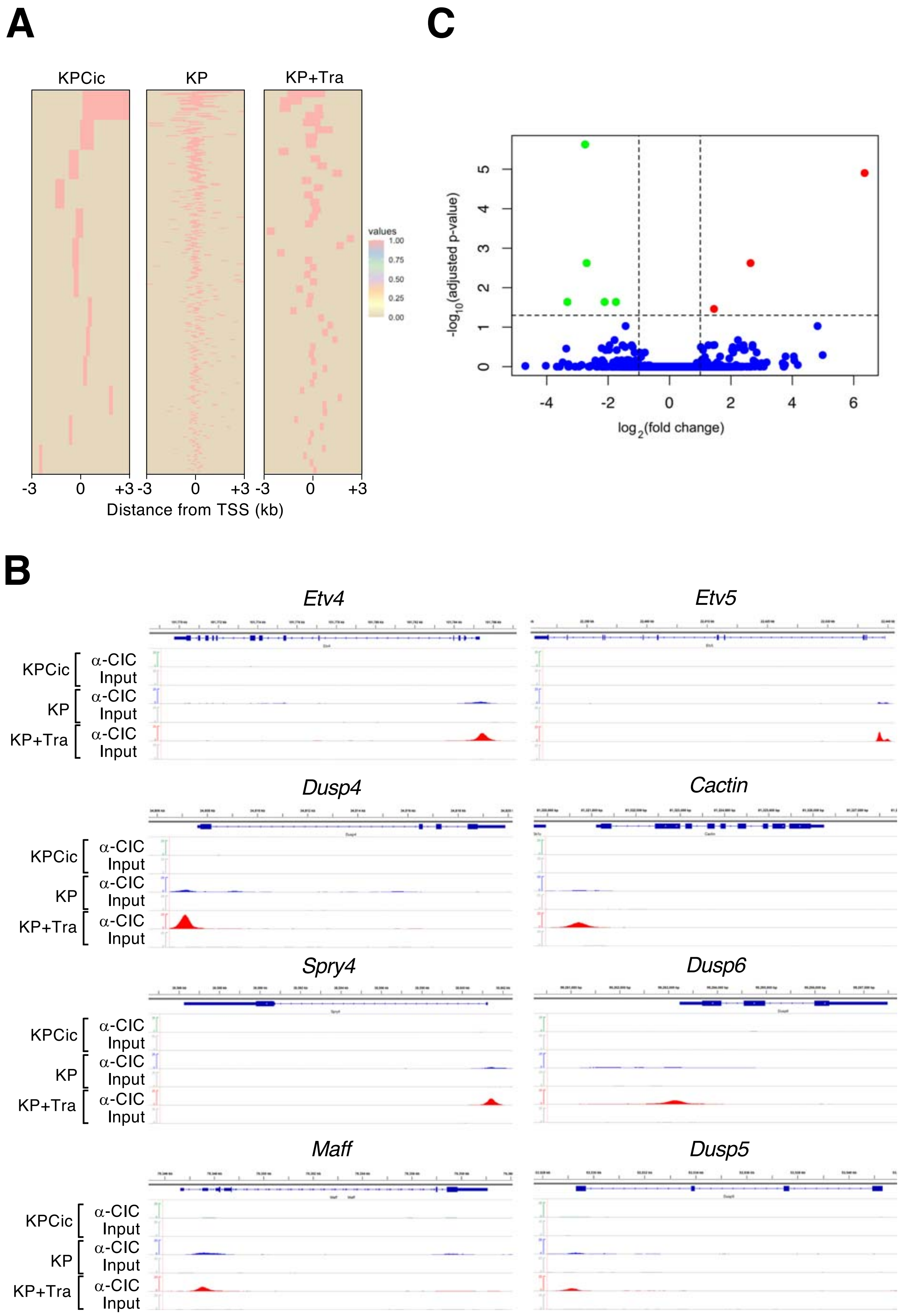
A
KPCic
KP
KP+Tra
values
1.00
0.75
0.50
0.25
0.00
-3
0
+3
Distance from TSS (kb)
C
-log10(adjusted p-value)
log2(fold change)
B
Etv4
Etv5
Dusp4
Cactin
Spry4
Dusp6
Maff
Dusp5
KPCic
KP
KP+Tra
α-CIC
Input

 © The Author(s)

**Figure EV2. ChIP- and RNA-seq analyses of KP and KPCic tumors.**

(**A**) Heatmap showing the distribution of CIC peak distances to the nearest transcriptional start site as determined by ChIPseeker in KPCic cells, KP cells and KP cells treated with 100 nM trametinib for 24 h. (**B**) ChIP-seq normalized coverage of CIC binding to representative promoters in KPCic cells, KP cells and KP cells treated with 100 nM trametinib for 24 h. The Y-axis values indicate the mean of normalized reads per 10 bp, using BPM with bamCoverage of deepTools. (**C**) Volcano plot of gene expression changes in KP ($n = 5$) vs. KPCic ($n = 5$) lung tumors obtained 5 months after infection with Ad-Cre. Statistical analysis was performed using the likelihood ratio test as implemented in the Bioconductor R package edgeR. To control the false discovery rate, $p$ values were adjusted using the Benjamini–Hochberg method.

© The Author(s)

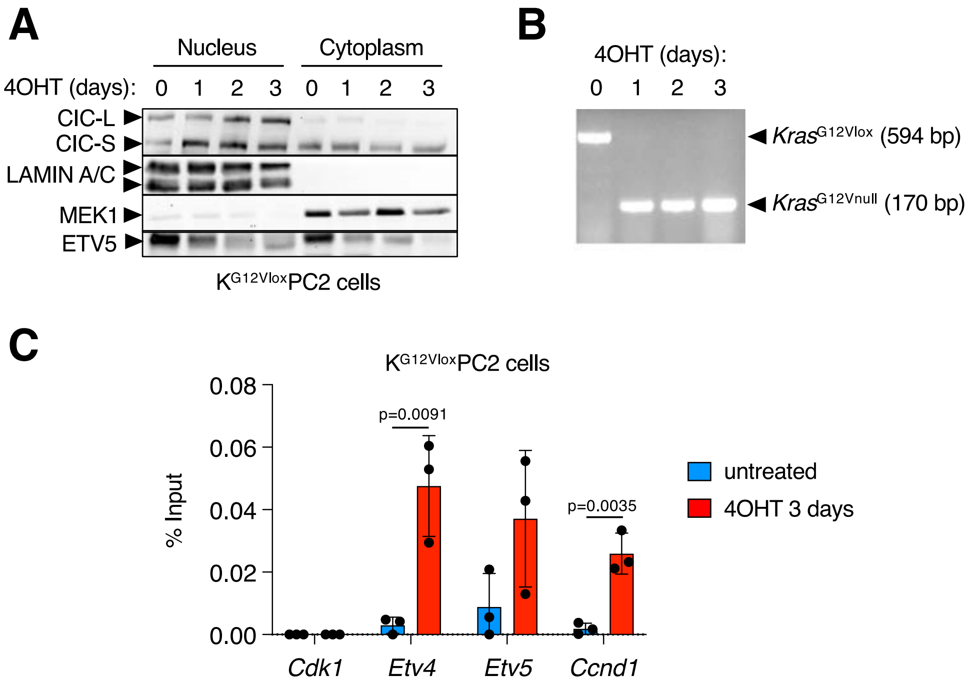


**Figure EV3. Control of CIC activity by oncogenic KRAS signaling in lung cancer cells**

(**A**) Western blot analysis of CIC and ETV5 expression in cytoplasmic and nuclear fractions of $K^{G12Vlox}$PC2 cells treated with 4-hydroxytamoxifen (4OHT) for the indicated time. MEK1 expression served as a marker for the cytoplasmic fraction and Lamin A/C expression for the nuclear fraction. (**B**) PCR analysis to confirm excision of the *Kras*$^{G12Vlox}$ allele in $K^{G12Vlox}$PC2 cells treated with 4OHT for the indicated time. *Kras*$^{G12Vlox}$ (594 bp) and *Kras*$^{G12Vnull}$ (170 bp) alleles are indicated. (**C**) Chromatin immunoprecipitation assay using CIC antibodies in untreated $K^{G12Vlox}$PC2 cells (blue bars) and $K^{G12Vlox}$PC2 treated with 4OHT for 3 days (red bars). Binding to CIC-binding sites (CBS) in the *Etv4*, *Etv5* and *Ccnd1* promoters as well as the *Cdk1* promoter which lacks CBS was analyzed by qRT-PCR and normalized to the amount of input DNA. Results are shown as mean ± SD. Statistics, unpaired *t*-test ($n = 3$ biological replicates).



© The Author(s)

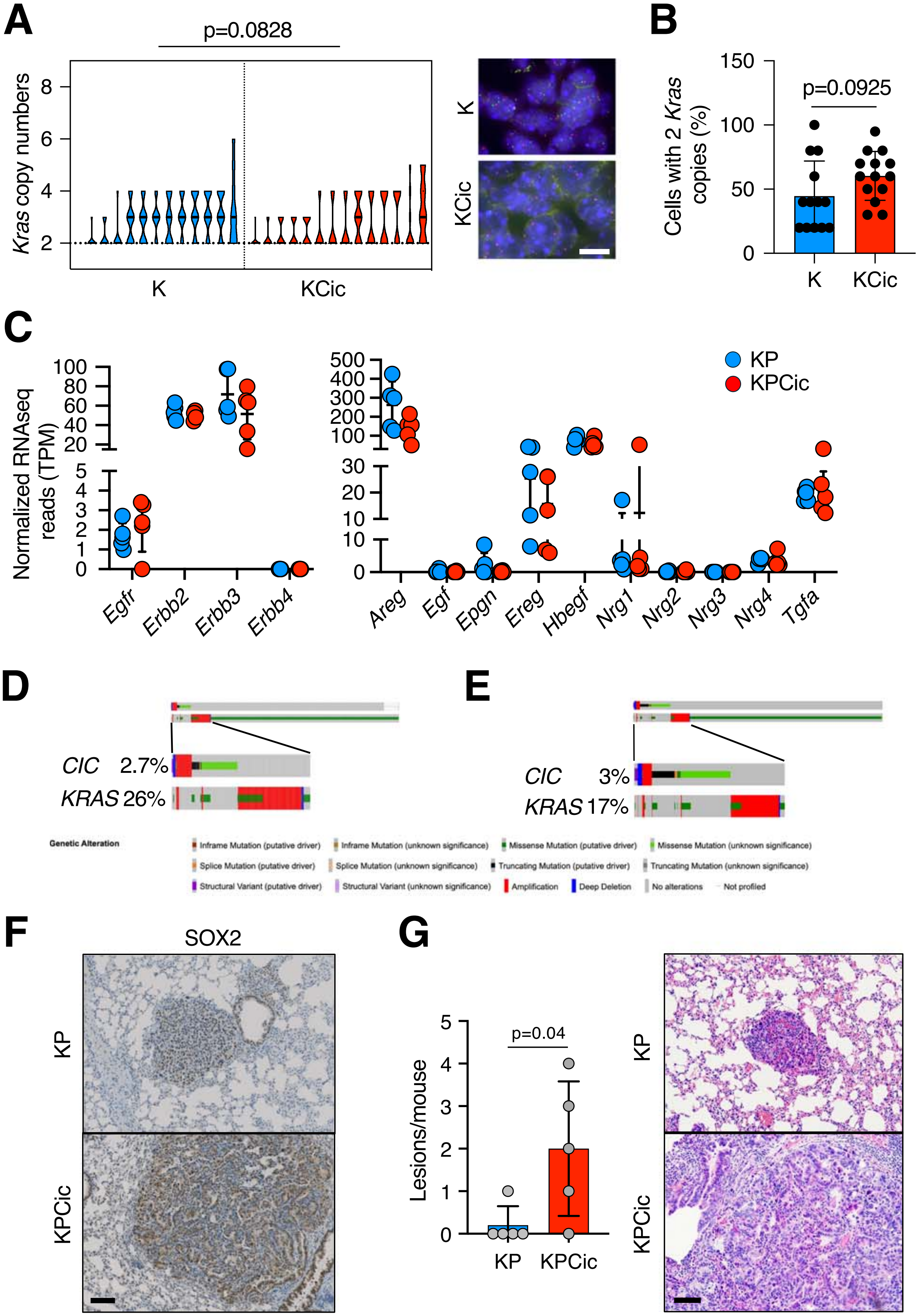
A
p=0.0828
Kras copy numbers
K
KCic
B
Cells with 2 Kras copies (%)
p=0.0925
K
KCic
C
Normalized RNAseq reads (TPM)
KP
KPCic
Egfr
Erbb2
Erbb3
Erbb4
Areg
Egf
Epgn
Ereg
Hbegf
Nrg1
Nrg2
Nrg3
Nrg4
Tgfa
D
CIC 2.7%
KRAS 26%
E
CIC 3%
KRAS 17%
Genetic Alteration
Inframe Mutation (putative driver)
Inframe Mutation (unknown significance)
Missense Mutation (putative driver)
Missense Mutation (unknown significance)
Splice Mutation (putative driver)
Splice Mutation (unknown significance)
Truncating Mutation (putative driver)
Truncating Mutation (unknown significance)
Structural Variant (putative driver)
Structural Variant (unknown significance)
Amplification
Deep Deletion
No alterations
Not profiled
F
SOX2
KP
KPCic
G
Lesions/mouse
p=0.04
KP
KPCic

**Figure EV4. Characterization of the impact of *Cic* deletion.**

(**A**) Left, quantification of *Kras* copy numbers by FISH staining in sections from lung tumors in K ($n = 12$ tumors, $n = 3$ mice; blue symbols) and KCic ($n = 14$ tumors, $n = 3$ mice, red symbols) mice obtained 10–12 months after infection with Ad-Cre. Statistics, $X^2$ test. Right, representative interphase FISH images of tumors obtained from K and KCic mice 10–12 months after infection with Ad-Cre. Red, *Kras* probe. Green, chromosome 6 probe. Scale bar, 5 µm. (**B**) Quantification of tumor cells retaining *Kras* 2 N in samples from (**A**). Statistics, unpaired *t*-test. (**C**) Normalized RNA-seq reads of the indicated genes from five independent KP as well as KPCic tumors obtained 5 months after infection with Ad-Cre. Results are shown as mean ± SD. Statistics, multiple *t*- tests. (**D**) Mutations in *CIC* and *KRAS* in human LUAD samples obtained from the TCGA database. (**E**) Mutations in *CIC* and *KRAS* in human Pan-Cancer samples obtained from the TCGA database. (**F**) Representative SOX2 IHC stainings in tumors from KP or KPCic mice 5 months after infection with Ad-CC10-Cre. Scale bar, 100 µM. (**G**) Left: Quantification of tumors in KP ($n = 5$) and KPCic ($n = 5$) mice 4 months after infection with Ad-CC10-Cre. Results are shown as mean ± SD. Statistics, unpaired *t*-test. Right, representative H&E images of tumors from KP or KPCic mice 4 months after infection with Ad-CC10-Cre. Scale bar, 100 µM.

 © The Author(s)

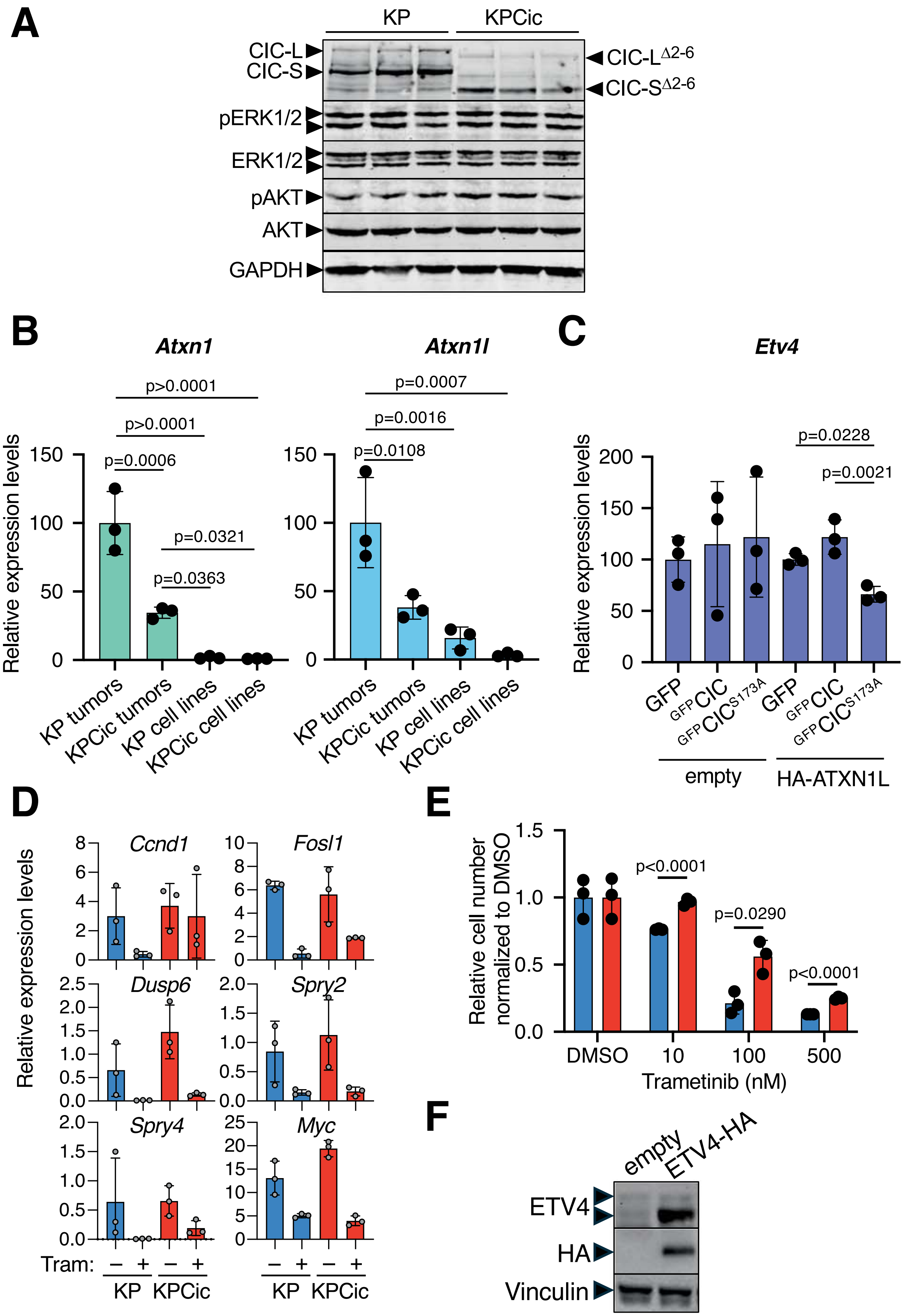
A
KP
KPCic
CIC-L
CIC-S
CIC-L$^{\Delta 2-6}$
CIC-S$^{\Delta 2-6}$
pERK1/2
ERK1/2
pAKT
AKT
GAPDH
B
Atxn1
Atxn1l
Relative expression levels
p>0.0001
p>0.0001
p=0.0006
p=0.0321
p=0.0363
p=0.0007
p=0.0016
p=0.0108
KP tumors
KPCic tumors
KP cell lines
KPCic cell lines
C
Etv4
p=0.0228
p=0.0021
GFP
GFPCIC
GFPCIC S173A
empty
HA-ATXN1L
D
Ccnd1
Fosl1
Dusp6
Spry2
Spry4
Myc
Tram:
KP
KPCic
E
Relative cell number normalized to DMSO
p<0.0001
p=0.0290
p<0.0001
DMSO
10
100
500
Trametinib (nM)
F
empty
ETV4-HA
ETV4
HA
Vinculin

**Figure EV5. Analysis of signaling pathways in KP and KPCic cell lines.**

(**A**) Western blot analysis of CIC, pERK1/2, ERK1/2, pAKT and AKT expression in KP and KPCic cell lines. GAPDH expression served as a loading control. (**B**) qRT-PCR of *Atxn1* and *Atxn1l* expression in KP tumors ($n = 3$), KPCic tumors ($n = 3$), KP cell lines ($n = 3$), and KPCic cell lines ($n = 3$). Results are shown as mean ± SD. Statistics, one-way ANOVA ($n = 3$ technical replicates). (**C**) qRT-PCR of *Etv4* expression in a KPCic cell line either infected with empty lentiviruses or lentiviruses stably expressing HA-ATXN1L 72 h after infection with Ad-GFP, $Ad^{GFP}CIC$ or $Ad^{GFP}CIC^{S173A}$. Results are shown as mean ± SD. Statistics, one-way ANOVA ($n = 3$ technical replicates). (**D**) qRT-PCR of the indicated genes in KP (blue bars, $n = 3$) and KPCic cell lines (red bars, $n = 3$) treated with DMSO (–) or 20 nM trametinib (Tram) for 24 h (+). Results are shown as mean ± SD. (**E**) Proliferation of a KP cell line infected with empty retroviruses (blue bars) or pLPC-ETV4-HA (red bars), 4 days after treatment with DMSO, 10 nM trametinib, 100 nM trametinib or 500 nM trametinib. Results are shown as mean ± SD. Statistics, multiple *t*-tests ($n = 3$ technical replicates). (**F**) Western blot analysis of ETV4-HA expressing using ETV4 and HA antibodies in KP cells a KP cell line infected with empty retroviruses or pLPC-ETV4-HA. Vinculin expression served as a loading control.

 © The Author(s)
